# Type-1 and type-2 decisions feature computational noise of similar magnitude

**DOI:** 10.1038/s44271-026-00454-3

**Published:** 2026-04-16

**Authors:** Yunxuan Zheng, Kai Xue, Medha Shekhar, Dobromir Rahnev

**Affiliations:** 1 School of Psychology, Georgia Institute of Technology, Atlanta, GA USA; 2 Center for Research in Cognition & Neurosciences, ULB Neuroscience Institute, Université Libre de Bruxelles, Brussels, Belgium

**Keywords:** Human behaviour, Computational models

## Abstract

Mounting evidence supports the existence of Type-2, metacognitive noise that affects human confidence judgments. The existence of this noise has led to the hypothesis that metacognitive judgments arise from a metacognitive system that is separate from the decision-making system responsible for Type-1 decisions. However, Type-2 decisions are different than standard Type-1 decisions in that they require evaluating the strength of sensory evidence rather than just making a simple categorical judgment. Here, we investigated whether Type-2 computational noise still exceeds Type-1 computational noise when both judgments require evaluation of the strength of sensory evidence. Participants (*N* = 319) performed a perceptual discrimination task and in different conditions provided Type-2 confidence judgments or Type-1 judgments based on biased expectations or unequal rewards. All these judgments require a similar fine-grained evaluation of the strength of sensory evidence (e.g., is the evidence high enough to justify a high-confidence rating or choosing the alternative with smaller probability or reward). We first confirmed that the confidence and expectation conditions resulted in similarly biased criteria and perceptual sensitivity drop, whereas the reward conditions exhibited less biased criteria and smaller perceptual sensitivity drop. Critically, formal computational modeling demonstrated that all conditions exhibited comparable levels of computational noise. These findings demonstrate that Type-1 and Type-2 decisions exhibit computational noise of similar magnitude when they require similar fine-grained evaluation of sensory evidence strength. More generally, our results have implications for the debate on whether Type-1 and Type-2 decisions are made by separate systems.

## Abstract (web summary)

Zheng et al. show that confidence (Type-2) judgments feature the same magnitude of computational noise as perceptual (Type-1) judgments, with important implications for the debate on whether Type-1 and Type-2 decisions are made by separate systems.

## Introduction

Humans can both judge the identity of sensory stimuli (type-1 perceptual decision making) and evaluate the likelihood that their judgments are correct (type-2 metacognitive decision making)^1–3^. Although people’s type-2 confidence ratings generally track their type-1 accuracy well, ample evidence has also indicated that type-2 ratings carry less information than the type-1 decision itself^4–6^. Computationally, such inefficiency of confidence ratings has been modeled as metacognitive noise, a type of computational noise that is assumed to only affect confidence ratings but not the perceptual decision^5,7–9^.

The existence of type-2 metacognitive noise has been argued to support the two-system view of cognition and metacognition^8,10^. The two-system view proposes that type-2 judgments arise from a distinct metacognitive system that evaluates outputs from primary cognitive processes (type-1 judgments), contrasting with the one-system view, which posits a single cognitive system underlying both types of judgments^2,10–12^. The presence of computational noise specific to type-2 judgments has thus been interpreted as evidence for the two-system view^10,13^.

However, this interpretation may overlook a key factor in the underlying computation. That is, typical type-1 decisions only require participants to determine which category the stimulus belongs to, type-2 decisions demand a fine-grained evaluation of whether the strength of that evidence is sufficiently high to justify high confidence^14–18^. Thus, it remains plausible that a single decision-making system generates both judgment types, but the fine-grained evaluation of the strength of sensory evidence accounts for increased noise in type-2 judgments. A critical test would involve comparing computational noise levels in type-1 and type-2 judgments that both require similar fine-grained decisions.

One way to transform type-1 judgments from category judgments to judgments that require fine-grained evaluation of the strength of sensory evidence is by introducing expectation and reward information. Indeed, information about prior stimulus probability or reward compels individuals to judge whether the sensory evidence is sufficient to accept or override the information provided by the expectation or reward cues^19–28^.

**Fig. 1:**
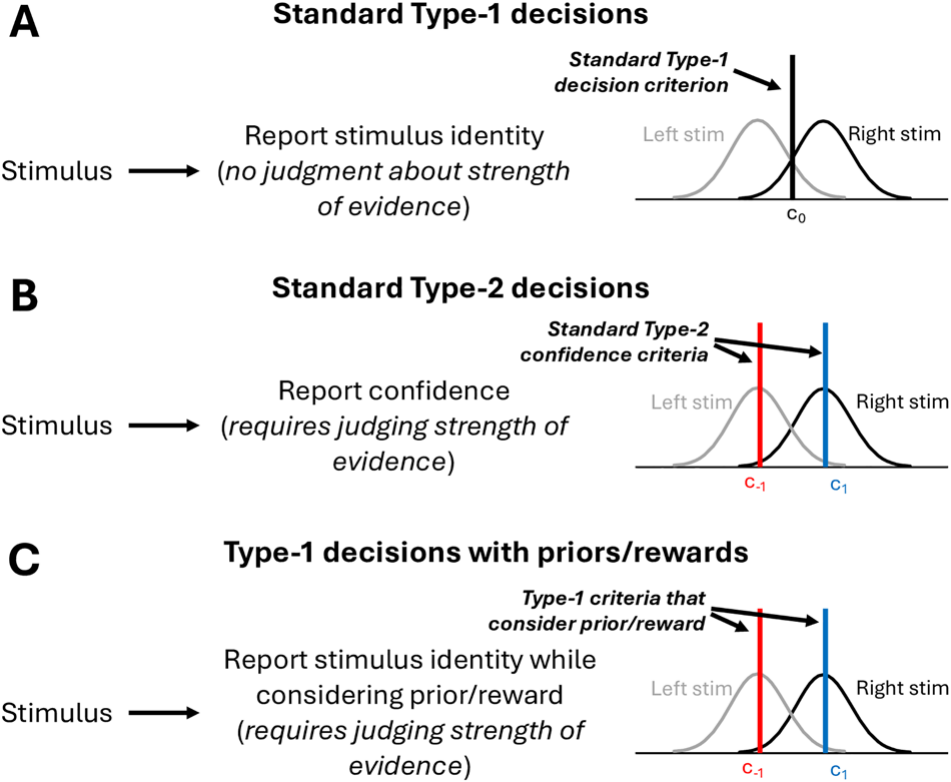
Evaluation of the strength of sensory evidence in different kinds of type-1 and type-2 decisions. **A** Standard type-1 decisions only require the individual to determine which category the stimulus belongs to. In practice, this usually means that a participant simply needs to select the category with higher evidence. Under the signal detection theory (SDT) framework, this is equivalent to placing a decision criterion at the point where the two stimulus categories are equally likely (*c*_0_). **B** In contrast, type-2 confidence judgments require a fine-grained evaluation of whether the strength of evidence is sufficient to justify high confidence. In the SDT framework, this is equivalent to placing confidence criteria (*c*_−1_ and *c*_1_) in locations that are sufficiently shifted compared to *c*_0_ as to justify giving a high confidence rating. **C** Type-1 decisions with expectation or reward cues also involve fine-grained evaluation of the sensory evidence strength, as they require participants to judge whether the strength of evidence is sufficient to justify overriding the bias induced by the expectation or reward cues. For example, overriding a cue that favors the “left” option is analogous to having high confidence that the “right” option is correct. In the SDT framework, this is equivalent to placing shifted decision criteria (*c*_−1_ and *c*_1_) in potentially the same locations as the confidence criteria from (**B**).

In fact, perceptual decision making with type-2 confidence judgment could be considered as equivalent to type-1 decision making biased by expectations about the stimulus or unequal rewards for each stimulus category. According to signal detection theory (SDT), expectation and reward cues induce a shift in the decision criterion, such that people are more liberal to report the choice favored by the cues^28–32^. Similarly, confidence judgments are also produced by placing confidence criteria that are shifted with respect to the decision criterion^17^. The criterion placement for decision making under expectation and rewards, and that for confidence judgments, can be similar when the expected payoff ratio indicated by the cue matches the subjective likelihood ratio of being correct (Fig. 1). For instance, an ideal observer would shift their decision criterion by a comparable magnitude when facing a cue that offers three times the payoff for correctly identifying the left versus right stimulus and when reporting “right” with over 75% confidence. In both cases, reporting “right” requires 75% or greater certainty that the stimulus was indeed on the right (to either override the greater payoff for left or to motivate the high confidence rating). Such comparable shifts in type-1 decisions biased by instruction cues and type-2 confidence judgments suggest that similar fine-grained evaluations of the strength of sensory evidence may underlie both types of decisions. This parallel, in turn, allows us to examine whether the computational noise associated with these criterion placements is also of similar magnitude.

Several previous studies have examined how confidence is given in the presence of expectation or reward cues^33–40^. Normatively, confidence should be influenced by expectation cues (which carry information about the stimulus) but not by reward cues (which do not)^36^. Yet, several recent studies demonstrated that both types of cues influence confidence for most participants^35,36,38^. A related line of research has demonstrated that expectation cues have a larger effect on confidence than on the initial perceptual judgment^33,34^. However, these studies examined how expectations and rewards influence confidence and did not explore whether computational noise is similar for type-1 judgments biased by expectation or reward cues vs. standard type-2 judgments (where confidence is given in the absence of biasing cues).

One important consideration related to the computational noise in type-1 judgments with expectation and reward cues is the timing of the cues. Specifically, such instruction cues are typically given before the sensory stimulus and are usually modeled as affecting either the starting point^31,41,42^ or the drift rate^30,31,43–45^ of evidence accumulation. However, when a cue is presented after the sensory evidence (i.e., once evidence has already been accumulating for a while), it may have limited impact on the starting point or drift rate but instead affect the decision threshold or post-decisional evidence accumulation. In other words, because the cue’s influence on evidence accumulation depends on its timing, the magnitude of computational noise may also vary with the temporal order between the cue and the sensory evidence. Type-2 confidence judgments are typically provided after the sensory decision and are sometimes modeled as being based on a post-decisional evidence accumulation^46–49^. Therefore, to comprehensively compare computational noise associated with expectation cues, reward cues, and confidence ratings, it is important to separately examine the effects of expectation and reward cues that appear before vs. after the stimulus^29^.

Here, we investigate whether type-1 and type-2 decision making feature computational noise of similar magnitude when both types of judgments require similar fine-grained evaluation of the sensory evidence strength. Participants performed a perceptual task that incorporated in different conditions, either (1) a pre-stimulus expectation cue, (2) a post-stimulus expectation cue, (3) a pre-stimulus reward cue, (4) a post-stimulus reward cue, or (5) confidence ratings. We hypothesized that the computational noise affecting type-2 metacognitive judgments is of similar magnitude as the computational noise affecting type-1 perceptual judgments when two types of judgments involve similar fine-grained evaluation on sensory evidence strength.

## Methods

### Preregistration

We preregistered the sample size, exclusion criteria, study design, analysis plan, and hypotheses (https://osf.io/ryjq8/) on March 8, 2022.

### Participants

We recruited 319 participants (189 females, 130 males; self-reported) via the Prolific platform. Participants were aged 18–75 (mean = 35.06, SD = 12.98). Ethnicity was self-reported, including 263 Hispanic or Latino, 39 not Hispanic or Latino, and 17 preferred not to answer. Race was also self-reported: 3 American Indian, 21 Asian, 45 black, 1 Hawaiian, 216 white, 24 chose “other”, and 9 chose “prefer not to answer”.

The experiment lasted around an hour, and each participant was paid $7.5 as basic participation fee. Participants were also additionally awarded a bonus based on their task performance. Experimental procedures were approved by the Georgia Institute of Technology Institutional Review Board, and participants signed informed consent at the beginning of the experiment.

To ensure data quality, we pre-registered four exclusion criteria. Specifically, we planned to exclude participants who: (1) exhibited overall accuracy < 55% correct; (2) reported a decision option in more than 80% of trials across the whole experiment; (3) reported the same confidence level in more than 95% of trials; (4) failed three out of five attention checks; or (5) exhibited reaction times (RTs) longer than 3 s on more than 20% of trials. However, these exclusion criteria resulted in retaining several negative $d\hbox{'}$ values for individual conditions, which could lead to an overestimation of computational noise in the model fitting process. Therefore, we instead applied these same criteria separately for each condition (except exclusion criterion 4 related to attention checks, which was still applied in the same way as pre-registered), thus ensuring that no negative $d\hbox{'}$ values remained. In addition to participant exclusions, as pre-registered, we also removed individual trials with RT longer than 3 s. After applying the exclusion criteria, the final number of participants for each condition was: confidence (*N* = 288), pre-expectation (*N* = 301), post-expectation (*N* = 296), pre-reward (*N* = 273), and post-reward (*N* = 257). We confirmed that all our conclusions remain the same when applying the original exclusion criteria (Fig. S1) or stricter exclusion criteria where a participant’s data was excluded from all conditions as long as it had to be excluded from even a single condition (Fig. S2).

### Procedure

**Fig. 2:**
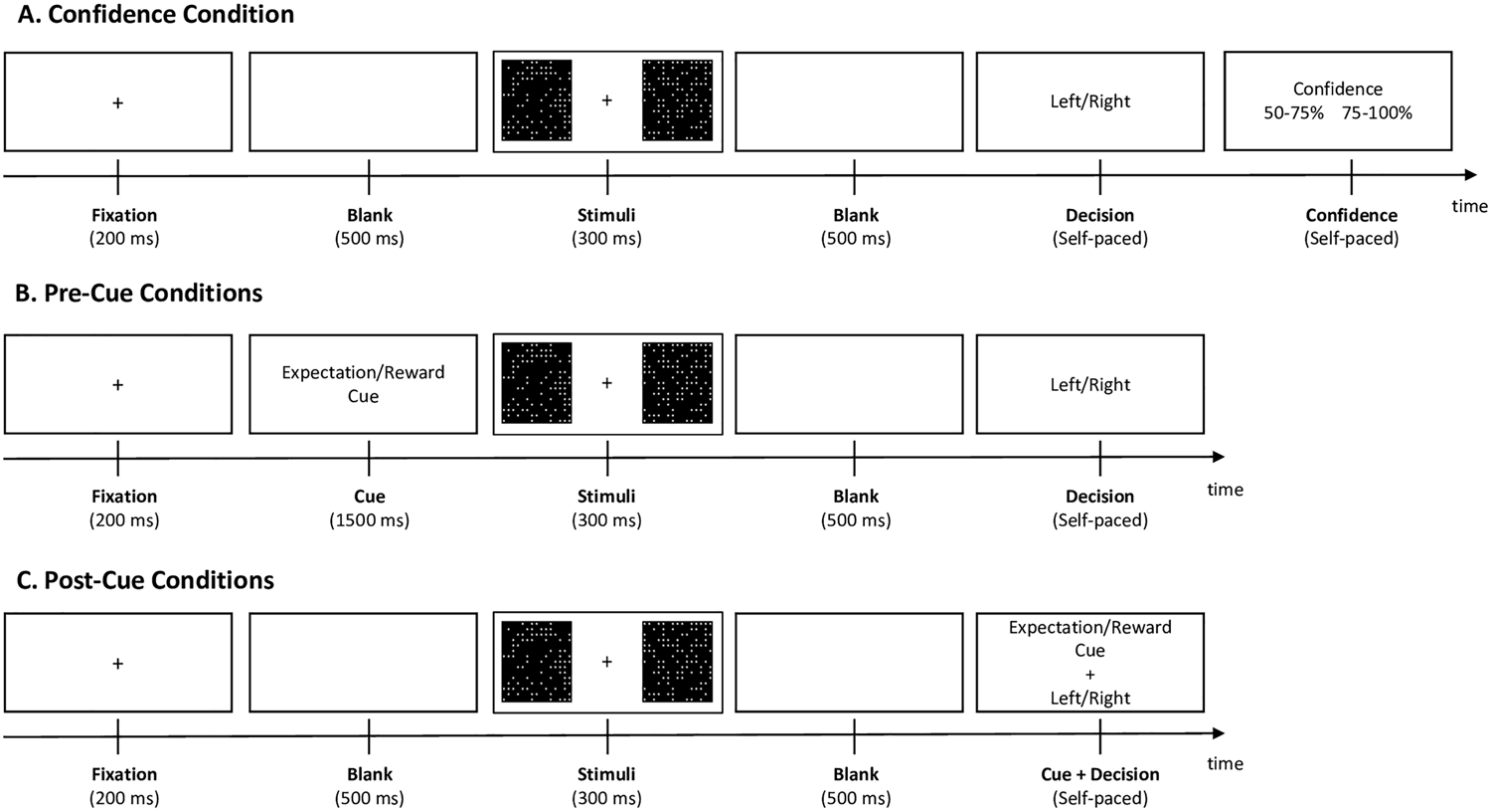
Task paradigm*.* **A** Confidence condition. Participants judged which of the two squares contains more dots and reported their decision confidence. **B** Pre-cue conditions. Participants completed the same dots task but saw an expectation or reward cue prior to the stimulus presentation. **C** Post-cue conditions. Participants completed the same dots task but saw an expectation or reward cue after the stimulus presentation. In all conditions, responses were self-paced.

Participants completed a two-alternative forced choice (2AFC) task. On each trial, two black squares were presented on the left and right sides of the screen in an imaginary 15 × 15 grid. One square contained 100 white dots randomly assigned to the grid, while the other square contained 85 dots (Fig. 2). Each trial began with a 200-ms fixation period. The stimuli were presented for 300 ms and followed immediately by a 500-ms blank screen. Participants indicated which square contained more dots. The position of the square with more dots (i.e., left or right) was randomized on each trial. We included five different conditions: confidence, pre-expectation, post-expectation, pre-reward, and post-reward.

In the confidence condition, we included a 500-ms blank screen between the fixation and stimulus periods. Participants first made their perceptual decision using an untimed button press. Immediately after, they reported their confidence level on a binary scale: low confidence (i.e., 50–75% probability of being correct) or high confidence (75–100% probability of being correct). These confidence ranges were displayed on the screen during the confidence report to remind participants of the intended mapping. Participants completed a total of 100 trials in the confidence condition.

In the expectation and reward conditions, we manipulated the temporal order between the cue and stimuli. In the pre-cue conditions, the expectation or reward cue (details regarding the cues are described below) was presented for 1500 ms immediately before the visual stimuli. The cue presentation time is longer than the blank screen prior to the stimulus in the post-cue and confidence conditions to allow participants sufficient time to fully process the cue information. In the post-cue conditions, the expectation or reward cue was presented after the visual stimuli and simultaneously with the response prompt (“left/right”). We presented cues with response prompt simultaneously because presenting separate post-cue and decision screens may lead to the post-cue not getting properly processed (if the post-cue duration is too short) or unnecessarily slow down the decision (if the post-cue duration is too long). Participants did not provide a confidence rating in any of the cue conditions.

The reward conditions contained biased and unbiased reward cues. The unbiased reward cues indicated that correct left and right choices would both result in winning 1 point, whereas the biased reward cues indicated that one of the choices would win 3 points. The cue was presented by indicating the number of points associated with each stimulus (either “1 point” or “3 points”) above each stimulus location. The expectation conditions similarly contained biased and unbiased expectation cues. The unbiased expectation cue indicated that the left and right options were equally likely (“two sides equally likely” on the screen), whereas the biased expectation cues indicated that one of the choices was 3 times more likely than the other (e.g., “left is 3 times more likely” on the screen). In reality, the biased expectation cues were valid on 66% of the trials to ensure that we could collect sufficient number of trials with invalid cues. Participants did not seem to be affected by this slight mismatch between the instruction and true validity rate, as their criterion placement was identical between the first and second halves of the experiment (Fig. S3). Each reward and expectation conditions had 150 trials (for a total of 600 trials across all four cue conditions), equally split between cues favoring the left choice, cues favoring the right choice, and unbiased cues. Except in the reward conditions with biased cues, all correct answers resulted in winning 1 point. Incorrect answers always resulted in not winning or losing any points.

Notably, in all conditions, participants were told that correct responses would allow them to accumulate points across the experiment; however, type-1 decision feedback was not provided at the end of each trial in any of the conditions. In the end of the experiment, participants were given a bonus based on the total number of points earned.

Participants underwent the experimental conditions sequentially, with the sequence of task conditions randomized across participants. The expectation and reward conditions consisted of three blocks of 50 trials. Within these conditions, the 150 total trials per condition were evenly divided into left-biased, right-biased, and neutral cues, and were shown in three blocks of 50 trials where the three cue types were randomly interleaved. The confidence condition then consisted of two blocks of 50 trials, with a total of 100 trials. The trial number in the confidence condition was lower than each of the four cue conditions. This is because in the cue conditions, each type of cue only contributed to the estimation of a single criterion (the unbiased decision criterion and the two biased criteria), while 100 trials in the confidence condition contributed to the estimation of all 3 criteria. At the start of each condition, participants were informed about which condition they were about to complete. Each condition started with 20 practice trials to aid familiarization. The practice trials were not included in the analyses and are not counted in the trial numbers presented above.

To screen inattentive participants, we included an attention check at the end of each condition. The attention checks were regular trials, except that participants were instructed to choose the square on the right side instead of picking the one with higher number of dots. The attention checks were not randomly added in the middle of a block to minimize any undue influence on subsequent trials via serial dependence or other mechanisms. Given the high number of trials per condition and the fact that participants were not informed when a condition would end before an attention check, inattentive participants would still be likely to miss the attention check if they just made random responses. The attention check trials were not included in the analyses and are not counted in the trial numbers presented above.

The experiment was programmed using the jsPsych library (version 5.0.3)^50^. To ensure that the stimulus size was consistent for all participants, we employed an established calibration procedure^7^ where participants positioned the computer monitor at an arm’s distance (approximately 60 cm) and adjusted the size of a credit card displayed on the computer screen to match the actual object’s dimensions in real life.

### Statistical analysis

The experiment used a 2AFC design. SDT generalizes straightforwardly to 2AFC by modeling the decision as a comparison of two internal responses, with the higher response determining the choice. Accordingly, we computed the SDT parameter decision sensitivity (*d’*)^15,17^:

1 $${d}^{{\prime} }={\varPhi }^{-1}\left({\mathrm{hit}}\,{\mathrm{rate}}\right)-{\varPhi }^{-1}\left({\mathrm{false}}\,{\mathrm{alarm}}\,{\mathrm{rate}}\right)$$

where Φ^-1^ is the inverse of the cumulative standard normal distribution that transforms hit rate and false-alarm rate into z scores.

We also computed the SDT parameter decision criterion (*c*) to quantify the bias in reporting one choice alternative over the other:

2 $$c=-\frac{1}{2}\left({\varPhi }^{-1}\left({\mathrm{hit}}\,{\mathrm{rate}}\right)+{\varPhi }^{-1}\left({\mathrm{false}}\,{\mathrm{alarm}}\,{\mathrm{rate}}\right)\right)$$

In the cue conditions, we computed $d\hbox{'}$ and c separately for trials with cues favoring the left choice, cues favoring the right choice, and unbiased cues. To match the $d\hbox{'}$ and c estimation procedure across all conditions, we transformed the responses from the confidence condition in three different ways to match the three types of cues in the cue conditions. For instance, a left-biased instruction cue makes participants more likely to make a left choice, which means that the criterion has shifted to the right (Fig. 1C).

To allow for a direct comparison between type-1 and type-2 decisions, data from the confidence condition were subsequently re-coded to match the structure of the cue conditions. Specifically, in the confidence reports, the confidence criterion separating low and high confidence for right responses acts similarly to a biased decision criterion obtained when using a left-biased instruction cue (Fig. 1B). Indeed, in both cases participants report right (for the left-biased instruction cue) or right with high confidence (for confidence reports) only when there is very strong evidence for a right choice. Therefore, to match the left-biased instruction cues, “right” choices with high confidence were then treated as “right” choices, while all other responses were treated as “left” choices. Similarly, to match the right-biased instruction cues, “left” choices with high confidence were treated as “left” choices, while all other responses were treated as “right” choices (Fig. 1B, C). Finally, to match the unbiased instruction cues, we retained the original choices, disregarding the confidence ratings (Fig. 1A, B). All trials in the confidence condition were used for each recoding process.

We calculated the degree of $d\hbox{'}$ drop as:

3 $${d}_{{\mathrm{drop}}}^{{\prime} }={{d}^{{\prime} }}_{0}-\frac{{d}_{-1}^{{\prime} }+{d}_{1}^{{\prime} }}{2}$$

where ${{d}^{{\prime} }}_{1}$, ${{d}^{{\prime} }}_{-1}$, and ${{d}^{{\prime} }}_{0}$ are the $d\hbox{'}$ values for left-biased, right-biased, and unbiased instruction cues.

The degree of criterion shift (${c}_{{\mathrm{shift}}}$) was calculated as:

4 $${c}_{{\mathrm{shift}}}={c}_{1}-{c}_{-1}$$

where ${c}_{1}$ and ${c}_{-1}$ are the criterion values associated with the left and right-biased instruction cues.

It should be noted that there were circumstances where the hit rate or false alarm rate was one or zero, leading to NaN values for c and *d’*. One common method of addressing this issue is by applying Hautus’s correction^51^ to avoid the zero hit rare and false alarm rate^15,17^. Nevertheless, these types of corrections are imperfect and can lead to systematic misestimation of $d\hbox{'}$ ^5^. Therefore, here we chose not to apply any corrections and instead exclude all NaN values. When such exclusions occurred, we computed $d\hbox{'}$ drop and criterion shift using modified formulas. Specifically, for the $d\hbox{'}$ drop calculation, if ${{d}^{{\prime} }}_{1}={\mathrm{NaN}}$, then we used the formula ${d}_{{\mathrm{drop}}}^{{\prime} }={{d}^{{\prime} }}_{0}-{d}_{-1}^{{\prime} }$, whereas if ${{d}^{{\prime} }}_{-1}={\mathrm{NaN}}$, then we used the formula ${d}_{{\mathrm{drop}}}^{{\prime} }={{d}^{{\prime} }}_{0}-{d}_{1}^{{\prime} }$. However, if both ${{d}^{{\prime} }}_{1}$ and ${{d}^{{\prime} }}_{-1}$ were NaN, or ${{d}^{{\prime} }}_{0}$ was NaN, then the $d\hbox{'}$ drop in this condition was excluded from further analyses. Similarly, for the criterion shift calculation, if ${c}_{1}={\mathrm{NaN}}$, then we used the formula ${c}_{{\mathrm{shift}}}=2\left({c}_{0}-{c}_{-1}\right)$, whereas if ${c}_{-1}={\mathrm{NaN}}$, then we used the formula ${c}_{{\mathrm{shift}}}=2\left({c}_{1}-{c}_{0}\right)$. However, if both ${c}_{1}$ and ${c}_{-1}$ were NaN, or ${c}_{0}$ was NaN, then the c shift in this condition was excluded from further analyses. We used the modified formulas for 70, 68, 54, 57, and 52 participants in the confidence, pre-expectation, post-expectation, pre-reward, and post-reward conditions, respectively (note that if $c={\mathrm{NaN}}$, then ${d}^{{\prime} }={\mathrm{NaN}}$, and vice versa, so the number of modified formulas is the same for ${c}_{{\mathrm{shift}}}$ and ${d}_{{\mathrm{drop}}}^{{\prime} }$). The final sample sizes for the analyses of criterion shift and $d\hbox{'}$ drop were 229, 207, 197, 210, and 211 participants in the confidence, pre-expectation, post-expectation, pre-reward, and post-reward conditions, respectively. Notably, we found qualitatively similar results when applying Hautus’s edge correction and not using the modified formulas to compute criterion shift and $d\hbox{'}$ drop (Fig. S4).

As preregistered, we conducted one-sided one-sample t-tests to determine whether the average criterion shift and $d{\prime}$ drop in each condition were significantly greater than zero. To account for the missing values (i.e., the NaN values) across conditions, we further employed mixed-effects linear models to perform pairwise comparisons of criterion shift and $d{\prime}$ drop. We did not apply corrections for multiple comparisons to the planned pairwise contrasts. This approach was chosen because our study was designed to test specific, pre-defined hypotheses guided by the existing literature rather than to conduct a broad set of exploratory analyses. The *d’* drop and criterion shift data generally satisfied the normality assumption of our parametric tests, except for a slight deviation for the criterion shift values in the pre-reward condition. However, for consistency, we conducted parametric testing for all conditions. Parametric tests are highly robust to small deviations from normality at large sample sizes (*N* = 210 in this case) without inflating type I error rates.

We note that we pre-registered analyses investigating whether the degree of criterion shift and $d\hbox{'}$ drop correlated across the five conditions. However, we found that the $d\hbox{'}$ drop produced very low split-half reliability (Fig. S5). Low split-half reliabilities force low values for between-condition correlation, which makes between-condition correlations uninterpretable. Therefore, we provide the pre-registered correlation results in Fig. S6 but do not discuss them further in the main paper. Further, we pre-registered that we would perform Bayesian correlation equality tests for criterion shift and $d\hbox{'}$ drop between different conditions. However, it turned out that the BFpack package in R that we planned to use does not support such analyses, and we were unable to find any software to allow us to perform this analysis.

To quantify the relative evidence for each between-condition comparison, we computed Bayes factors (BF). In the case of analyses involving linear mixed models, BFs were computed as the ratio of the estimated marginal likelihoods between the alternative (H₁) and null (H₀) Bayesian multilevel models using bridge sampling within the “brms” R package. For one-sample and paired t-tests, the BFs were calculated using the “BayesFactor” R package.

Finally, to evaluate the optimality of decision-making and assess the degree of conservatism, we also calculated a participant’s theoretical optimal criterion (${c}_{{\mathrm{opt}}}$) that maximizes expected gain for each cue type under each condition (again confidence condition was recoded as a cue condition). The ${c}_{{\mathrm{opt}}}$ was computed as $\frac{{\mathrm{ln}}({\beta }_{{\mathrm{opt}}})}{d{\prime} }$, where $d{\prime}$ represents the sensitivity associated with the left-biased or right-biased criterion (${c}_{1}$ or ${c}_{-1}$), and ${\beta }_{{\mathrm{opt}}}$ represents the optimal expected payoff ratio of a biased instruction cue, which equals three for all cue conditions (i.e., ${\beta }_{{\mathrm{opt}}}=3$). To note, for the right-biased instruction cue type, we took the negative value of the ${c}_{{\mathrm{opt}}}$. The degree of conservatism was defined as the tendency for the observer’s actual criterion (${c}_{1}$ or ${c}_{-1}$) to be placed closer to the neutral point than the calculated optimal criterion (${c}_{{\mathrm{opt}}}$). The distributions of ${c}_{{\mathrm{opt}}}$ were skewed due to small *d’* values, therefore, to statistically compare the observed biased criteria against their corresponding optimal criteria, we conducted nonparametric Wilcoxon signed-rank tests for each cue type in each condition.

### Computational model

The model is adapted from the lognormal meta-noise model^5^. This model builds upon standard SDT, with an additional assumption that computational noise drawn from a lognormal distribution is added to confidence criteria or decision criteria influenced by expectation or reward cues. This added computational noise results in variability of criterion placement across trials.

#### Evidence distributions

Following standard SDT assumptions, we assume that each stimulus category generates noisy sensory evidence, x, drawn from Gaussian distributions. For stimuli from category *S*_1_ (i.e., the left square containing more dots in this case), the evidence is drawn from $x \sim N\left(\right.-\frac{{\mu }_{{\mathrm{sens}}}}{2}$, ${\sigma }_{{\mathrm{sens}}}^{2}$) and for stimuli from category *S*_2_ (i.e., the right square containing more dots), evidence is drawn from $x \sim N\left(\right.\frac{{\mu }_{{\mathrm{sens}}}}{2}$, ${\sigma }_{{\mathrm{sens}}}^{2}$) where ${\mu }_{{\mathrm{sens}}}$ represents the distance between the two evidence distributions and ${\sigma }_{{\mathrm{sens}}}$ is the standard deviation of the distributions (set to 1 without loss of generality).

#### Decision generation

The primary perceptual decision is generated by comparing the sensory evidence x with a decision criterion *c*_0_, such that $x < {c}_{0}$ leads to response “*S*_1_“ and $x\ge {c}_{0}$ leads to response “*S*_2_“. The decision criterion *c*_0_ captures any response bias, with ${c}_{0}=0$ indicating no bias.

#### Lognormal computational noise

Confidence judgments in the current study were generated using a set of confidence criteria [*c*_−__1_,*c*_1_]. The key assumption of the lognormal meta noise model^5^ is that these confidence criteria are not fixed but vary from trial to trial according to a lognormal distribution. This variability represents metacognitive noise: sources of uncertainty that selectively affect confidence but not the primary decision. The lognormal meta noise model can be extended to the type-1 criteria biased by expectation and reward instruction cues. The formulas below apply to ${c}_{-1}$ and ${c}_{1}$ regardless of condition (confidence vs. cue conditions).

The criteria ${c}_{i}$ follow a lognormal probability distribution, g, though the formulas for *c*_−1_ and ${c}_{1}$ are slightly different since they fall on opposite sides of the decision criterion. Specifically, ${c}_{1}$ follows the following distribution:

5 $${c}_{1} \sim g\left({y}|{\mu }_{1},{\sigma }_{{\mathrm{comp}}}^{2}\right)=\frac{1}{\left.(y-{c}_{0})\right) \sqrt{2\pi {\sigma }_{{\mathrm{comp}}}^{2}}}\\ \exp \left(-\frac{({\mathrm{ln}}{(y-{c}_{0})+{\mu }_{1}})^{2}}{2{\sigma }_{{\mathrm{comp}}}^{2}}\right),y\in \left({c}_{0},\infty \right)$$

and ${c}_{-1}$ follows the following distribution:

6 $${c}_{-1} \sim g\left({{y|}\mu }_{-1},{\sigma }_{{\mathrm{comp}}}^{2}\right)=\frac{1}{\left({c}_{0}-y\right)\sqrt{2\pi {\sigma }_{{\mathrm{comp}}}^{2}}} \\ \exp \left(-\frac{({\mathrm{ln}}{({c}_{0}-y)+{\mu }_{-1}})^{2}}{2{\sigma }_{{\mathrm{comp}}}^{2}}\right),y\in \left(-\infty ,{c}_{0}\right)$$

where ${\mu }_{-1}$ and ${\mu }_{1}$ are the means of the Gaussian random variable obtained by taking log of ${c}_{-1}$ and ${c}_{1}$ (with the constraint that ${\mu }_{-1} < {c}_{0} < {\mu }_{1}$), and ${\sigma }_{{\mathrm{comp}}}$ is the standard deviation of this random variable, which serves as a measure of the amount of computational noise. Note that ${\sigma }_{{\mathrm{comp}}}$ quantifies the amount of computational noise in the log domain, not in the raw evidence domain.

#### Response probability calculation

To compute the log-likelihood of the data, the model must determine the probability of a participant’s “left” or “right” response given a specific stimulus (*S*_*1*_ or *S*_2_) and criterion (${{c}_{-1},c}_{0},{c}_{1}$). The formulas vary based on the criterion, but are the same regardless of whether stimulus *S*_*1*_ or *S*_*2*_ was presented. Below, we give the probabilities of providing the “left” response; the probability of responding “right“ is simply 1 minus the probability of responding “left”.

For the fixed neutral criterion ${c}_{0}$, the probability of a “left” response given stimulus *S* is equal to the probability that the sensory evidence x is less than ${c}_{0}$:

7 $$P(x < {c}_{0}{|S})=\varPhi \left({c}_{0}\big|{\mu }_{s},{\sigma }_{{\mathrm{sens}}}^{2}\right)$$

where Φ is the cumulative normal distribution, ${\mu }_{s}$ is the mean of the stimulus S distribution (${\mu }_{s}=-\frac{{\mu }_{{\mathrm{sens}}}}{2}$ if $S={S}_{1}$ and ${\mu }_{s}=\frac{{\mu }_{{\mathrm{sens}}}}{2}$ if $S={S}_{2}$), and ${\sigma }_{{\mathrm{sens}}}=1$ is the standard deviation of this distribution.

The formulas are more complex for the noisy criteria ${c}_{-1}$ and ${c}_{1}$. In these cases, the probability of a “left” response equals the probability that the sensory evidence x is less these criteria, which are themselves described by the distributions $y \sim g({\mu }_{-1},{\sigma }_{{comp}}^{2})$ and $y \sim g({\mu }_{1},{\sigma }_{{\mathrm{comp}}}^{2})$. Thus, the formula for the probability of a ‘Left’ response for criterion ${c}_{-1}$ becomes:

8 $$P(x < g(y| {\mu }_{-1},{\sigma }_{{\mathrm{comp}}}^{2}) | {S})={\int }_{x=-\infty }^{{c}_{0}}{\int }_{y=x}^{{c}_{0}}\varPhi \left(x|{\mu }_{s},{\sigma }_{{\mathrm{sens}}}^{2}\right)g \left(y|{\mu }_{-1},{\sigma }_{{\mathrm{comp}}}^{2}\right){dy}\,{dx}$$

In the case of criterion ${c}_{1}$, it is simpler to give the formula for the probability of a “right” response (the probability of responding “left” is simply 1 minus the probability of responding “right”), which is:

9 $$P(x > g(y| {\mu }_{1},{\sigma }_{{\mathrm{comp}}}^{2}) | {S})={\int }_{x={c}_{0}}^{\infty }{\int }_{y={c}_{0}}^{x}\varPhi \left(x|{\mu }_{s},{\sigma }_{{\mathrm{sens}}}^{2}\right) g \left(y|{\mu }_{1},{\sigma }_{{\mathrm{comp}}}^{2}\right){dydx}$$

#### Model fitting

The predicted probabilities for “left” and “right” responses for each stimulus and cue condition were compared to the observed trial counts to compute the log-likelihood for a given set of parameters. To fit the model, we employed the Bayesian adaptive direct search optimization algorithm^52^ to jointly fit all 5 free parameters: sensitivity, ${\mu }_{{\mathrm{sens}}}$; the neutral criterion, ${{{\rm{c}}}}_{0}$; the means of the criteria ${c}_{-1}$ and ${c}_{1}$ (i.e., parameters ${\mu }_{-1}$ and ${\mu }_{1}$); and the computational noise, ${\sigma }_{{\mathrm{comp}}}$. Fitting was performed by minimizing the negative log-likelihood of the data. To ensure a robust fitting process, the algorithm was initialized with parameter values of ${\mu }_{{\mathrm{sens}}}$, ${{{\rm{c}}}}_{0}$, ${\mu }_{-1}$, and ${\mu }_{1}$ derived from standard SDT calculations, while ${\sigma }_{{\mathrm{comp}}}$ was initialized to the value of 0.05. To ensure reliable and meaningful model fitting estimates, we only fit participants who exhibited positive $d{\prime}$ values for each of the three criteria (${c}_{-1},{c}_{0},{c}_{1}$) within a given cue condition, resulting in valid data from 155, 126, 123, 151, and 155 participants for confidence, pre-expectation, post-expectation, pre-reward, and post-reward conditions, respectively. Note that this smaller set of participants still showed the same pattern of results for the criterion shift and $d\hbox{'}$ drop effects as the full sample (Fig. S7).

#### Statistical analysis of model fits

Because the distributions of estimated noise values were highly skewed, we used permutation tests (randomly shuffling data 10,000 times) to compare the differences in noise magnitude across conditions. We additionally confirmed all results by also conducting bootstrapping analyses (resampling 10,000 times). Because the BayesFactor R package currently is only able to compute BF values for parametric tests, we did not report BF values here for these pairwise comparisons. We performed all analyses on both the original and the log-transformed noise values.

### Sensitivity power analysis and null result interpretation

To rigorously interpret nonsignificant findings, we conducted a sensitivity power analysis based on the Smallest Effect Size of Interest (SESOI), following the framework outlined by Lakens^53^. We defined our SESOI as Cohen’s *d* = 0.40, which is the median expected effect in within-subjects experimental psychology^54,55^. Across our analyses, the smallest valid sample size occurred in the pairwise linear mixed-effects models comparing computational noise in the post-expectation condition (*N* = 123). A sensitivity power analysis indicated that with *N* = 123, our study achieved 80% power (α = 0.05, two-tailed) to detect a standardized mean difference of *d* = 0.25. Because our minimum detectable effect (0.25) is substantially smaller than our theoretically justified SESOI (0.40), our analyses were highly powered to detect any theoretically meaningful effects. Consequently, we can confidently interpret nonsignificant (null) statistical outcomes in our models as evidence for the absence of a pragmatically meaningful effect, rather than a lack of statistical power.

## Results

We aimed to compare the magnitude of computational type-1 and type-2 decisions. To do so, we analyzed how confidence and biased instruction cues affected the (1) criterion shift, (2) decision sensitivity (d’) drop for confidence and biased criteria, and (3) estimated magnitude of computational noise in each condition. Within each type of analysis, we first compare type-2 confidence judgments to all other type-1 judgments with instruction cues (main analyses), then compare the pre- to post-stimulus cues (to examine predictions from evidence accumulation models), and finally compare expectations and reward cues (to test for differences among different ways of biasing type-1 decisions). For brevity, we often report multiple statistical tests together; exact *p*-values, effect size measures, 95% confidence intervals, and Bayes Factors (when applicable) for all tests are reported in Tables S1–S6.

### Criterion shift

According to SDT, perceptual decisions with confidence, expectation, and reward involve a similar kind of criterion placement (Fig. 1). Specifically, the decision criteria biased by expectation or reward, as well as the confidence criteria for distinguishing between low and high confidence, are all assumed to be shifted compared to the unbiased decision criterion. Therefore, as preregistered, we first tested whether such criterion shift occurred in each condition (a form of manipulation check). In the four cue conditions, we computed criterion shift as the difference in the values of the decision criteria for trials with biased instruction cues favoring the left vs. right options (i.e., ${c}_{1}-{c}_{-1}$). Similarly, in the confidence condition, we computed criterion shift as the difference in the values of the confidence criteria separating low and high confidence for left vs. right decisions. As preregistered, we used one-sided one-sample t-tests to examine whether the degree of criterion shift was positive in each condition.

#### Overall pattern of criterion shifts

**Fig. 3:**
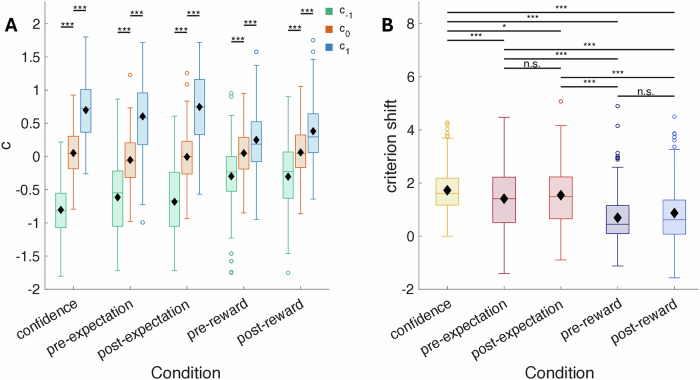
Criterion placements across conditions. **A** Criterion location in each condition for the three criterion locations (${c}_{-1},{c}_{0},{c}_{1}$). In the confidence condition, the left (right) criterion separates high vs. low confidence for right (left) responses, while the middle criterion corresponds to the decision criterion. In all conditions, the value for biased criteria was significantly different than the value for the unbiased criterion. **B** The degree of criterion shift, measured as ${c}_{1}-{c}_{-1}$, for each condition. In all conditions, the degree of criterion shift was significantly above zero. Additionally, the degree of shift varied across conditions. The analyses for confidence, pre-expectation, post-expectation, pre-reward, and post-reward were based on *N* = 229, 207, 197, 210, and 211, respectively. Box plots show 25th, 50th, and 75th percentiles (box lines), as well as minimum and maximum excluding outliers (whiskers). Individual dots show outliers. Diamonds show means. ***, *p* < .001; **, p < .01; and *, p < .05; n.s. not significant.

We found that the degree of criterion shift was significantly greater than 0 in all conditions (Fig. 3A and Table 1). In addition, each of the biased criteria was significantly different than the unbiased criterion for each of the five conditions (Table 1). We further tested whether criterion placement was conservative compared to the reward-maximizing ideal as found in prior studies^25,36,56^. We found that all biased criteria (${c}_{-1}$ and ${c}_{1}$) were placed conservatively (i.e., not as extreme as the optimal) in all conditions (Table 2).

**Table 1:** Criterion comparisons and criterion shift

Condition	Comparison	*t*	d*f*	*p*	Cohen’s *d*	95% CI	BF_10_
Confidence	*c*_−1_ vs. *c*_0_	−32.08	191	3.81 × 10^−79^	−2.48	[-Inf, −0.87]	1.45 × 10^75^
Confidence	*c*_1_ vs. *c*_0_	24.78	195	1.84 × 10^−62^	1.84	[0.66, Inf]	4.05 × 10^58^
Confidence	Shift vs. 0	32.82	228	1.20 × 10^−88^	2.16	[1.62, 1.83]	4.16 × 10^84^
Pre-expectation	*c*_−1_ vs. *c*_0_	−14.30	169	3.33 × 10^−31^	−1.22	[-Inf, −0.52]	4.71 × 10^27^
Pre-expectation	*c*_1_ vs. *c*_0_	14.57	175	2.63 × 10^−32^	1.46	[0.61, Inf]	5.74 × 10^28^
Pre-expectation	Shift vs. 0	18.22	206	4.10 × 10^−45^	1.26	[1.26, 1.56]	2.61 × 10^41^
Post-expectation	*c*_−1_ vs. *c*_0_	−14.88	168	8.88 × 10^−33^	−1.52	[-Inf, −0.61]	1.69 × 10^29^
Post-expectation	*c*_1_ vs. *c*_0_	17.12	170	3.90 × 10^−39^	1.67	[0.69, Inf]	3.18 × 10^35^
Post-expectation	Shift vs. 0	19.95	196	2.20 × 10^−49^	1.42	[1.39, 1.69]	4.40 × 10^45^
Pre-reward	*c*_−1_ vs. *c*_0_	−9.78	193	6.48 × 10^−19^	−0.86	[-Inf, −0.29]	3.82 × 10^15^
Pre-reward	*c*_1_ vs. *c*_0_	6.82	168	8.01 × 10^−11^	0.55	[0.17, Inf]	5.12 × 10^7^
Pre-reward	Shift vs 0	10.90	209	1.75 × 10^−22^	0.75	[0.57, 0.82]	1.20 × 10^19^
Post-reward	*c*_−1_ vs. *c*_0_	−9.81	189	6.27 × 10^−19^	−0.91	[-Inf, −0.32]	3.96 × 10^15^
Post-reward	*c*_1_ vs. *c*_0_	9.58	179	4.08 × 10^−18^	0.85	[0.29, Inf]	6.38 × 10^14^
Post-reward	Shift vs. 0	12.01	210	6.29 × 10^−26^	0.82	[0.73, 1.01]	2.92 × 10^22^

**Table 2:** Conservatism analysis: actual vs. optimal criterion

Condition	Side	Median actual *c*	Median optimal *c*	*Z*-val	*N*	*p*	Effect size *r*
Confidence	*c*_1_	0.73	2.00	−12.14	196	6.50 × 10^−34^	0.87
Confidence	*c*_−1_	−0.82	−2.22	12.02	192	2.94 × 10^−33^	0.87
Pre-expectation	*c*_1_	0.58	0.76	−8.58	176	9.70 × 10^−18^	0.65
Pre-expectation	*c*_−1_	−0.55	−0.79	8.76	170	1.99 × 10^−18^	0.67
Post-expectation	*c*_1_	0.75	0.95	−7.53	171	5.25 × 10^−14^	0.58
Post-expectation	*c*_−1_	−0.67	−0.91	8.71	169	2.91 × 10^−18^	0.67
Pre-reward	*c*_1_	0.19	0.69	−9.94	169	2.90 × 10^−23^	0.76
Pre-reward	*c*_−1_	−0.23	−0.67	10.83	194	2.62 × 10^−27^	0.78
Post-reward	*c*_1_	0.30	0.73	−10.09	180	6.14 × 10^−24^	0.75
Post-reward	*c*_−1_	−0.23	−0.74	10.57	190	4.23 × 10^−^^26^	0.77

#### Type-2 vs. type-1 judgments

The criterion shift was significantly larger in the confidence condition than each of the four cue conditions (Fig. 3B). This difference was the smallest for the post-expectation condition, with the Bayes Factor indicating lack of strong evidence (Table 3). In contrast, the differences between confidence and the other three cue conditions were statistically much stronger (Table 3). These results show that criterion placement was generally more extreme for type-2 compared to biased type-1 decisions.

**Table 3:** Criterion shift pairwise comparisons

Comparison	*t*	d*f*	*p*	Cohen’s *d*	95% CI	BF_10_	BF_01_
Confidence vs. pre-expectation	3.47	244.18	6.16 × 10^−4^	0.22	[0.14, 0.50]	58.587	0.017
Confidence vs. post-expectation	2.15	251.00	0.033	0.14	[0.02, 0.36]	1.589	0.629
Confidence vs. pre-reward	13.59	238.87	1.51 × 10^−31^	0.88	[0.89, 1.19]	7.01 × 10^28^	1.43 × 10^−^^29^
Confidence vs. post-reward	10.22	243.59	1.23 × 10^−20^	0.65	[0.69, 1.02]	9.33 × 10^17^	1.07 × 10^−^^18^
Pre-expectation vs. post-expectation	−1.78	184.27	0.077	−0.13	[−0.29, 0.01]	0.701	1.427
Pre-expectation vs. pre-reward	8.17	199.06	3.54 × 10^−14^	0.58	[0.52, 0.86]	3.98 × 10^11^	2.51 × 10^−12^
Pre-expectation vs. post-reward	5.56	215.35	8.06 × 10^−8^	0.38	[0.34, 0.71]	2.20 × 10^5^	4.54 × 10^−^^6^
Post-expectation vs. pre-reward	9.56	202.01	4.21 × 10^−18^	0.67	[0.68, 1.03]	6.25 × 10^14^	1.60 × 10^−^^15^
Post-expectation vs. post-reward	6.72	212.19	1.64 × 10^−10^	0.46	[0.47, 0.86]	7.75 × 10^7^	1.29 × 10^−^^8^
Pre-reward vs. post-reward	−1.89	189.84	0.060	−0.14	[−0.34, 0.01]	0.978	1.023

#### Pre-cues vs. post-cues

Prior research found that post-cues tend to result in larger biasing effects^29^, which would mean larger criterion shifts in the current study. However, while our data showed a similar trend with post-cues producing numerically higher criterion shifts, there was little credible evidence that the criterion shift value differed between when the cue was presented before vs. after the stimulus in either the reward conditions or the expectation conditions (Table 3).

#### Expectation vs. reward

Criterion shift in each of the two expectation conditions was greater than in each of the two reward conditions (Table 3). This result replicates many previous studies showing that participants tend to shift their criteria less when given reward compared to expectation cues^25,36^.

### $d\hbox{'}$ drop for biased criteria

Having established that all five conditions led to substantial criterion shifts, we next examined whether the decision sensitivity (*d’*) associated with biased criteria and confidence criteria was lower than the $d\hbox{'}$ for the unbiased criterion in each condition. To note, a standard assumption in the field is that biased expectation and reward cues do not change the estimated $d\hbox{'}$ value^15,17,36^. However, several studies have found that computational noise for the biased criteria results in a drop in estimated stimulus sensitivity ($d{\prime}$) compared to the $d\hbox{'}$ for the unbiased criterion^57–59^. Therefore, as preregistered, we examined the degree of $d\hbox{'}$ drop across conditions.

#### Overall pattern

**Fig. 4:**
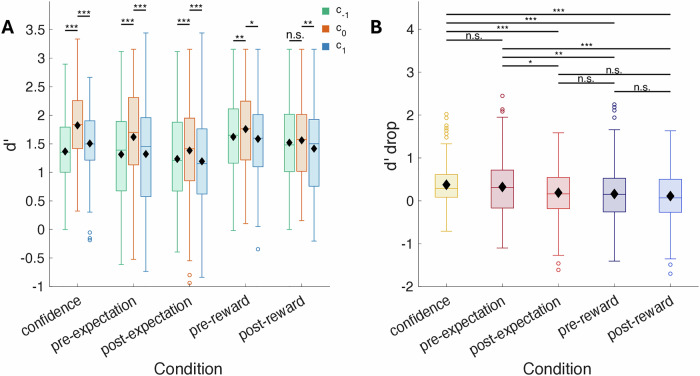
Decision sensitivity (*d’*) drop in each condition. **A** $d\hbox{'}$ values associated with left-biased (*c*_1_), right-biased (*c*_−1_), and unbiased instruction cues (*c*_0_). In the confidence condition, the left (right) $d\hbox{'}$ values correspond to the criterion separating high vs. low confidence for right (left) responses, while the middle $d\hbox{'}$ value corresponds to the decision criterion. In all conditions, the $d\hbox{'}$ for biased criteria was significantly lower than the $d\hbox{'}$ for the corresponding unbiased criterion. **B** The degree of $d\hbox{'}$ drop for each condition. In all conditions, the $d\hbox{'}$ drop was significantly above zero. Additionally, the degree of $d\hbox{'}$ drop varied across conditions. The analyses for confidence, pre-expectation, post-expectation, pre-reward, and post-reward were based on *N* = 229, 207, 197, 210, and 211, respectively. Box plots show 25th, 50th, and 75th percentiles (box lines), as well as minimum and maximum excluding outliers (whiskers). Individual dots show outliers. Diamonds show means. ***, *p* < 0.001; **, p < 0.01; and *, p < 0.05; n.s. not significant.

We found clear evidence for computational noise in the form of a $d{\prime}$ drop in all conditions (Fig. 4 and Table 4). Comparing each biased criterion to the unbiased criterion from the same condition, we found evidence for significant $d\hbox{'}$ drop in nine out of the 10 pairwise comparisons except for post-reward right vs. mid criteria (Fig. 4A and Table 4). The results for the confidence condition replicate previous findings about the presence of metacognitive noise in confidence judgments^5,60^, while the results for the expectation cues replicate previous such findings from the literature^57–59^. These findings indicate the existence of substantial computational noise in both type-1 and type-2 decisions.

**Table 4:** *d’* comparisons and *d’* drop

Condition	Comparison	*t*	d*f*	*p*	Cohen’s *d*	95% CI	BF_10_
Confidence	*c*_−1_ vs. *c*_0_	−11.13	191	8.17 × 10^−23^	−0.65	[-Inf, −0.33]	2.56 × 10^19^
Confidence	*c*_1_ vs. *c*_0_	−9.20	195	2.87 × 10^−17^	−0.49	[-Inf, −0.23]	9.29 × 10^13^
Confidence	Drop vs. 0	12.74	228	9.97 × 10^−29^	0.84	[0.32, 0.43]	1.66 × 10^25^
Pre-expectation	*c*_−1_ vs. *c*_0_	−6.23	169	1.77 × 10^−9^	−0.41	[-Inf, −0.25]	2.57 × 10^6^
Pre-expectation	*c*_1_ vs. *c*_0_	−5.13	175	3.79 × 10^−7^	−0.30	[-Inf, −0.17]	1.49 × 10^4^
Pre-expectation	Drop vs. 0	7.16	206	7.07 × 10^−12^	0.50	[0.23, 0.41]	5.07 × 10^8^
Post-expectation	*c*_−1_ vs. *c*_0_	−3.45	168	3.58 × 10^−4^	−0.20	[-Inf, −0.09]	24.344
Post-expectation	*c*_1_ vs. *c*_0_	−4.23	170	1.91 × 10^−5^	−0.25	[-Inf, −0.12]	369.743
Post-expectation	Drop vs. 0	4.69	196	2.57 × 10^−6^	0.33	[0.11, 0.27]	2310.871
Pre-reward	*c*_−1_ vs. *c*_0_	−2.85	193	0.002	−0.18	[-Inf, −0.05]	4.037
Pre-reward	*c*_1_ vs. *c*_0_	−2.12	168	0.018	−0.15	[-Inf, −0.02]	7.570
Pre-reward	Drop vs. 0	3.81	209	9.16 × 10^−5^	0.26	[0.08, 0.24]	78.380
Post-reward	*c*_−1_ vs. *c*_0_	−0.86	189	0.195	−0.06	[-Inf, 0.04]	0.117
Post-reward	*c*_1_ vs. *c*_0_	−2.73	179	0.004	−0.19	[-Inf, −0.05]	2.999
Post-reward	Drop vs. 0	2.66	210	0.004	0.18	[0.03, 0.19]	2.399

#### Type-2 vs. type-1 judgments

The degree of $d\hbox{'}$ drop in the confidence condition was significantly larger than in three of the four cue conditions, but was not significantly different compared to the pre-expectation condition (Fig. 4B and Table 5). This pattern closely mirrored the trend observed in criterion shifts (Fig. 3B), suggesting that the type-2 confidence judgments might be subject to higher level of computational noise due to the fact that they involve greater criterion shift.

**Table 5:** *d’* drop pairwise comparisons

Comparison	*t*	d*f*	*p*	Cohen’s *d*	95% CI	BF_10_	BF_01_
Confidence vs. pre-expectation	0.98	247.25	0.329	0.06	[−0.05, 0.16]	0.163	6.133
Confidence vs. post-expectation	3.85	246.19	1.53 × 10^−4^	0.25	[0.09, 0.28]	136.191	0.007
Confidence vs. pre-reward	4.34	250.95	2.03 × 10^−5^	0.27	[0.12, 0.31]	908.732	0.001
Confidence vs. post-reward	5.25	438.00	2.36 × 10^−7^	0.25	[0.17, 0.36]	7.16 × 10^4^	1.40 × 10^−^^5^
Pre-expectation vs. post-expectation	2.41	231.07	0.017	0.16	[0.03, 0.25]	1.827	0.547
Pre-expectation vs. pre-reward	2.74	214.91	0.007	0.19	[0.05, 0.28]	4.638	0.216
Pre-expectation vs. post-reward	3.47	416.00	5.85 × 10^−4^	0.17	[0.09, 0.33]	48.644	0.021
Post-expectation vs. pre-reward	0.49	405.00	0.624	0.02	[-0.09, 0.14]	0.136	7.367
Post-expectation vs. post-reward	1.35	406.00	0.179	0.07	[-0.04, 0.19]	0.303	3.301
Pre-reward vs. post-reward	0.92	208.93	0.360	0.06	[-0.06, 0.16]	0.162	6.175

#### Pre-cues vs. post-cues

We found a small but significant effect where the degree of *d’* drop in the pre-expectation condition was larger than in the post-expectation, but no such difference emerged when comparing the pre-reward and post-reward conditions (Fig. 4B and Table 5). Note that the pattern for $d\hbox{'}$ drop is in the opposite direction than the pattern for criterion shift (Fig. 3B), suggesting that the $d\hbox{'}$ drop results are not driven by the criterion shift differences. Thus, presenting the cues before versus after the sensory evidence appears to induce higher computational noise in decision making, though this effect may depend on the type of cue.

#### Expectation vs. reward

Finally, the degree of $d\hbox{'}$ drop in the pre-expectation condition was larger than the one in the pre-reward condition, but there was no credible evidence for a difference between the post-expectation and post-reward conditions (Table 5). This pattern resembles the criterion shift patterns (Fig. 3B), suggesting that the differences in $d\hbox{'}$ drop between expectation and reward cues might be driven by the differences in criterion shift.

### Magnitude of computational noise

**Fig. 5:**
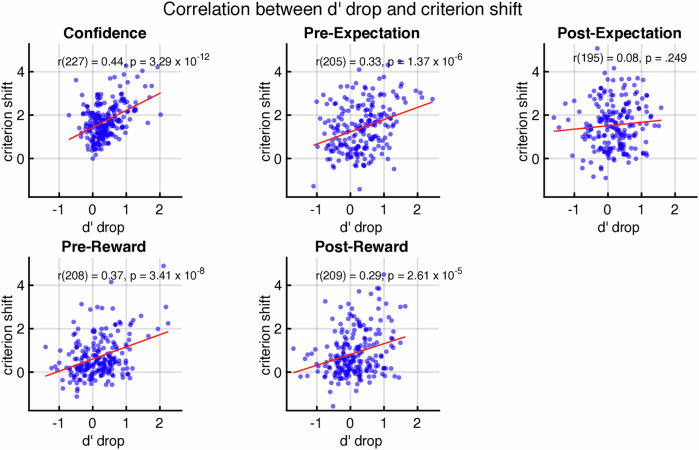
Correlations between criterion shift and *d’* drop. Each panel displays the relationship between the degree of criterion shift and $d\hbox{'}$ drop for a specific experimental condition. Each dot represents an individual subject. The red line indicates the linear regression fit, with Pearson’s *r* and *p*-values inset. As indicated by the *r* and *p*-values, significant positive relationships were observed between the degree of criterion shift and $d\hbox{'}$ drop in all conditions except for the post-expectation condition. The analyses for confidence, pre-expectation, post-expectation, pre-reward, and post-reward were based on *N* = 229, 207, 197, 210, and 211, respectively.

So far, we found substantial criterion shift and decision sensitivity ($d{\prime}$) drop in all conditions, confirming the presence of computational noise in different types of perceptual decision-making. Importantly, we also observed that the degree of $d\hbox{'}$ drop was approximately in proportion to the degree of criterion shift across many of the conditions. To further examine whether $d\hbox{'}$ drop and criterion shift are related, we correlated the degree of criterion shift and $d\hbox{'}$ drop across participants in each condition. We found significant positive correlations in four out of five conditions (Fig. 5 and Table 6). This finding aligns with predictions from the lognormal meta noise model proposed by Shekhar and Rahnev^5^, which posits that criterion placement noise follows a lognormal distribution and its variability increases with greater separation between confidence and decision criteria, consequently causing a larger drop in *d’* These empirical results and model considerations demonstrate that $d\hbox{'}$ drop is affected by the degree of criterion shift, leading to the conclusion that $d\hbox{'}$ drop is an imperfect measure of computational noise. To overcome this issue, we directly fitted our data with the lognormal meta noise model to estimate the magnitude of computational noise in criterion placement in each of the five conditions.

**Table 6:** *d’* drop vs. criterion shift correlations

Condition	*r*	d*f*	*p*	95% CI
Confidence	0.44	227	3.29 × 10^−12^	[0.33, 0.54]
Pre-expectation	0.33	205	1.37 × 10^−6^	[0.20, 0.44]
Post-expectation	0.08	195	0.249	[−0.06, 0.22]
Pre-reward	0.37	208	3.41 × 10^−8^	[0.25, 0.48]
Post-reward	0.29	209	2.61 × 10^−5^	[0.16, 0.40]

**Fig. 6:**
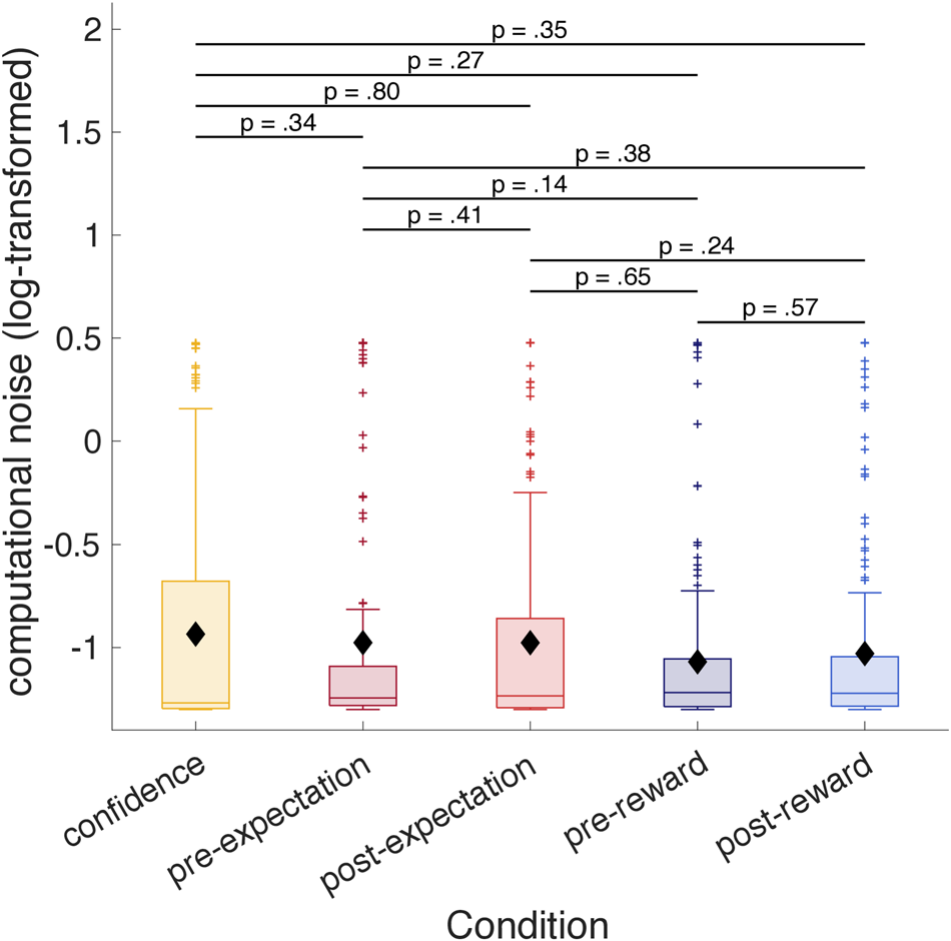
The magnitude of estimated computational noise for each condition. The computational noise was of similar magnitude across conditions. The figure shows log-transformed noise values for better visualization, but the statistical tests show equivalent results regardless of whether raw or log-transformed values are used (Tables S7 and S8). The analyses for confidence, pre-expectation, post-expectation, pre-reward, and post-reward were based on *N* = 155, 126, 123, 151, and 155, respectively. Box plots show 25th, 50th, and 75th percentiles (box lines), as well as minimum and maximum excluding outliers (whiskers). Individual dots show outliers. Diamonds show means. n.s. not significant.

We found no significant differences in computational noise between any pair of conditions (Table 7). Since the data were not normally distributed, these results were obtained using permutation tests. We further confirmed these results using bootstrapping (Table 7). In addition, we repeated all analyses after log-transforming the computational noise values and still obtained the same results (Fig. 6 and Table S1). Thus, although the numerical differences between conditions were consistent with the $d\hbox{'}$ drop results in Fig. 4B, our modeling results suggest that there are no significant differences in computational noise among any of the five conditions.

**Table 7:** Pairwise comparisons for raw computational noise values

Comparison	*N* pairs	*p* (permutation)	Boot 95% CI	*p* (bootstrap)
Confidence vs. pre-expectation	70	0.303	[−0.424, 0.129]	0.310
Confidence vs. post-expectation	68	0.682	[−0.153, 0.243]	0.680
Confidence vs. pre-reward	86	0.168	[−0.046, 0.299]	0.152
Confidence vs. post-reward	89	0.144	[−0.055, 0.337]	0.147
Pre-expectation vs. post-expectation	75	0.203	[−0.069, 0.370]	0.191
Pre-expectation vs. pre-reward	84	0.103	[−0.028, 0.431]	0.087
Pre-expectation vs. post-reward	88	0.086	[−0.022, 0.437]	0.075
Post-expectation vs. pre-reward	79	0.689	[−0.247, 0.148]	0.683
Post-expectation vs. post-reward	78	0.119	[−0.023, 0.283]	0.101
Pre-reward vs. post-reward	98	0.608	[−0.177, 0.104]	0.596

## Discussion

Type-2 confidence judgments have been found to be subject to metacognitive noise beyond the computational noise associated with type-1 perceptual decision making. However, compared to standard type-1 decisions, type-2 decisions require a more fine-grained evaluation of whether the strength of sensory evidence justifies a high confidence rating. It remains unclear whether type-1 decisions are subject to a similar level of computational noise as type-2 decisions when the two types of judgments require fine-grained evaluation of sensory evidence strength. Our study aimed to address this question by asking participants to perform a perceptual decision-making task, wherein, under varying conditions, participants either reported their type-2 confidence in the perceptual decisions or considered expectation or reward information to maximize payoffs in type-1 decisions. In both cases, the judgment requires a finer-grained evaluation of the strength of sensory evidence beyond a simple categorical choice. We found that confidence and expectation biased the criterion placement by a similar degree, though the reward cues led to a smaller criterion shift. Critically, computational modeling demonstrated that the magnitude of computational noise was similar between all conditions. These results suggest that when both type-1 and type-2 judgments require similar fine-grained evaluation of sensory evidence strength, the magnitude of type-2 computational noise is similar to the magnitude of type-1 computational noise.

### Relationship to previous research

Our study confirms and extends three findings from the literature. First, several previous studies have reported that $d\hbox{'}$ associated with expectation cues is lower than $d\hbox{'}$ associated with a neutral cue (what we call “$d\hbox{'}$ drop”)^29,57,58^. Nevertheless, to the best of our knowledge, this $d\hbox{'}$ drop has never been explained in the context of a computational model or linked to the equivalent $d\hbox{'}$ drop seen for confidence ratings^5^. Second, we also confirmed prior findings that reward cues have a smaller effect on criterion shift than expectation cues^36,56,61–63^. This finding is typically interpreted to imply that people prioritize accuracy over potential gains, hence showing greater reluctance to adjust their criteria in the presence of reward cues^25,36,56^. Third, we also found evidence that participants shifted their decision criterion less than what is optimal, replicating previous findings of conservative criterion placement^25,36^.

### Criterion shift is greatest for confidence

One surprising finding was that people generally shifted their criteria more in the confidence than in the expectation and reward conditions. This is despite the fact that the optimal criterion shift was identical across all conditions. Note, however, that the difference between the confidence and expectation conditions was much smaller (though significant) than the difference between the confidence and the reward conditions. As we reviewed above, the smaller criterion shift in the reward condition is likely due to participants prioritizing accuracy over the pure gain-maximizing strategy. It is possible that a similar, but smaller, effect leads to the difference between confidence and expectation: namely, participants may be prioritizing the sensory information over the probabilistic information from the expectation cue, whereas the confidence condition removes the need to prioritize one source of information over another. Nevertheless, the possibility is currently speculative and would need to be directly tested in future research.

### Extending the lognormal meta noise model to type-1 judgments

An important feature of our results is that the level of $d\hbox{'}$ drop is approximately proportional to the level of criterion shift. Indeed, we observed that the level of criterion shift was significantly correlated with the level of $d\hbox{'}$ drop across participants in four out of the five conditions. Furthermore, the $d\hbox{'}$ drop and criterion shift were largest in the confidence condition and smallest in the reward conditions. The association between these two variables is predicted by the lognormal meta noise model that assumes lognormal distributions of criterion noise^5,64^. Indeed, the existence of lognormal computational noise means that criteria placed further from the main decision criterion (${c}_{0}$) have greater variance, which in turn manifests as a more pronounced $d{\prime}$ drop. The modeling framework here extends the lognormal meta noise model to type-1 judgments with biased criteria and can capture this criterion shift and $d\hbox{'}$ drop correspondence across conditions.

### Relationship between pre and post-cues

Additionally, our study revealed largely similar patterns in type-1 decisions when expectation or reward cues were presented before versus after the sensory stimuli. Bang and Rahnev^29^ previously reported a larger criterion shift when expectation cues appeared after, compared to before, the sensory stimuli. In our study, we observed the same directional pattern. However, the differences were not statistically significant, though the Bayes factors indicated lack of clear evidence for either the null or the alternative hypotheses. More importantly, there was no difference in the estimated computational noise between the pre- and post-cue conditions (for either expectation or reward), though examining $d\hbox{'}$ drop did reveal a tendency for the pre-cue conditions to show larger $d\hbox{'}$ drop than post-cue conditions. Overall, these results suggest that pre- and post-cues are not equivalent, but that the differences between them are subtle.

### Implications for evidence accumulation models

The similarity between pre- and post-cues has implications about how evidence accumulation models, such as the drift diffusion model (DDM), conceptualize the influences of cues in perceptual decision making. The DDM posits that perceptual decision making is a process of accumulation terminated when the evidence reaches the same stereotyped boundary^65–68^. Within this framework, pre-cues are typically modeled as a change in the starting point or drift rate bias of the accumulation^30,31,41,42^, which means that noise in the pre-cue conditions should be modeled as noise in the starting point or drift rate bias adjustment. However, noise in the post-cue conditions cannot be modeled in the same way since post-cues are presented too late to meaningfully influence either the starting point or drift rate of the accumulation. Instead, noise in the post-cue conditions may be modeled as noise in the decision threshold. Since these are completely separate mechanisms, one may expect larger differences between pre- and post-cues in both criterion shift and computational noise than what was found in our data. Our results suggest that standard pre-cues may act not only by biasing the starting point or drift rate, but also by biasing late decisional processes. This conclusion mirrors the one by Sun and Landy^69^ who proposed that perceptual decisions may involve a 2-stage “estimate-then-decide” model. Within this framework, both pre- and post-cues may have minimal influence on the estimation stage and primarily affect the decision stage, which would explain why there are no large behavioral differences between pre- and post-cues. Our results thus point to a need of further research on the topic of how pre-cues affects decision making within evidence accumulation models.

### Implications to the cognitive architecture of metacognition

Our study also has implications for the debate about whether type-1 and type-2 decisions are made by the same system or different systems. The similar magnitude of the computational noise between type-1 perceptual and type-2 metacognitive decisions might challenge the dominant view that type-1 and type-2 judgments are operated by two distinct (though interrelated) systems^2,10,12^. This two-system perspective has been largely supported by the existence of confidence-accuracy dissociations, where various manipulations have been shown to affect type-2 confidence levels without affecting type-1 performance^70–76^. Nevertheless, such confidence-accuracy dissociations can often be modeled without assuming distinct systems^73,74,76–79^. Conversely, a few recent studies suggest that type-1 and type-2 judgments may be produced by a unified system. For instance, Gao et al.^80^ found that the way by which people use the uncertainty of sensory evidence to integrate multisensory signals into decisions is identical to how they use such uncertainty in confidence judgments. Moreover, Zheng et al^60^. provided evidence that a unified system might generate both metacognitive and meta-metacognitive judgments, a conclusion also consistent with the findings of Recht et al.^81^. By demonstrating the existence of similar levels of computational noise in type-1 and type-2 decisions, the current study further questions the necessity of postulating the presence of two separate systems for type-1 and type-2 judgments.

### Limitations

A central limitation in the current study is that it focuses solely on quantifying the magnitude of computational noise in type-1 and type-2 decisions but leaves open the question of whether the underlying sources of noise are identical. On one hand, the fact that the magnitude of computational noise is similar suggests the possibility that both types of judgments are corrupted by similar mechanisms. These mechanisms may include serial effects^82–84^, fluctuations in arousal or other internal states^85,86^, and random fluctuations in criterion placement^6,25,87^. On the other hand, the fact that two processes feature computational noise of similar magnitude does not necessitate the existence of common mechanisms. For example, inattention to the cue information might induce noise in the criterion placement in the cue conditions, while some other mechanisms might contribute to the noise in the confidence condition. Another limitation of our study is that the number of trials per participant in each condition was relatively small, reducing the reliability of each participant’s estimated noise magnitude^88^. Future research should use a larger number of trials and estimate the magnitude of computational noise for type-1 and type-2 decisions under different experimental paradigms to better establish the mechanisms controlling noise in type-1 vs. type-2 judgments.

## Supplementary information

Transparent Peer Review File

Supplementary Materials

## Data Availability

Data and materials are available at https://osf.io/3cm8s/.

## Code availability

The code for statistical analysis and model fitting is available at https://osf.io/3cm8s/.
