# Supplementary material for: Type-1 and type-2 decisions feature computational noise of similar magnitude
Source: Commun Psychol. 2026 Apr 16;4:113. doi: 10.1038/s44271-026-00454-3 (PMC13462944; doi:10.1038/s44271-026-00454-3)
Supplement: Supplementary file 1 [file 44271_2026_454_MOESM1_ESM.pdf]

nature portfolio

Peer Review File

Type-1 and Type-2 decisions feature computational noise of similar magnitude

Corresponding Author: Mr Yunxuan Zheng

This file contains all editorial decision letters in order by version, followed by all author rebuttals in order by version.

Version 0:

Decision Letter:

** Please ensure you delete the link to your author homepage in this e-mail if you wish to forward it to your coauthors **

Dear Mr Zheng,

Thank you for submitting your manuscript titled "Type-1 and Type-2 decisions feature computational noise of similar magnitude" to Communications Psychology. We have given the paper our careful consideration and find it of potential interest. However, due to certain shortcomings we are concerned that sending the current manuscript out to review could lead to unnecessary delays and quite possibly an undesirable outcome of the review process.

In particular, For manuscripts that interpret null results, we require Bayes Factors or equivalence tests to interpret the null results. As the central conclusion of the manuscript is based on the absence of the significant difference between the estimated computational noise between type 1 and type 2 decisions, please include appropriate statistics to support your interpretation of the absence of evidence. Please refer to our statistical guidelines for further information (https://www.nature.com/commspsychol/submit/submission-guidelines#statistical-guidelines).

Please also ensure to use appropriate language to describe the results. Statements such as 'There is no difference between x and y.' or 'X does not affect Y.' must be revised to read 'We found [no/little] credible evidence of a difference between x and y.' or 'We found [no/little] credible evidence that X affects Y.'

We would therefore like to invite you to revise your manuscript to address these concerns before we make a final determination on whether to send your manuscript for external review.

We shall hope to receive your revised version as soon as you are able to complete the suggested revisions. If something similar is published in the interim we will have to consider the impact it has on the novelty of a revised manuscript.

If you anticipate a delay of more than four weeks, please let us know. Should your manuscript be substantially delayed without notifying us in advance and your article is eventually published, the received date may be that of the revised, not the original, version.

We also ask that you ensure your manuscript complies with our editorial policies and reporting requirements.

To that end, we require revised manuscripts to be accompanied by a completed item: a reporting summary that collects information on study design and procedure.

- Nature Research Reporting Summary

Your revised manuscript can only be sent to referees if the checklist is completed and uploaded with the revision.

If you are not interested in submitting a suitably revised manuscript in the future please let me know immediately so we can close your file. If you have any questions, please contact me.

Please use the link below when you are prepared to resubmit.
Link Redacted

Thank you for your interest in Communications Psychology.

Best regards,
Troby Lui

Troby Lui, PhD
Associate Editor
Communications Psychology

Version 1:

Decision Letter:

** Please ensure you delete the link to your author homepage in this e-mail if you wish to forward it to your coauthors **

Dear Mr Zheng,

Thank you for your patience during the peer-review process. Your manuscript titled "Type-1 and Type-2 decisions feature computational noise of similar magnitude" has now been seen by 3 reviewers, and I include their comments at the end of this message. They find your work of interest but raised some important points. We are interested in the possibility of publishing your study in Communications Psychology, but would like to consider your responses to these concerns and assess a revised manuscript before we make a final decision on publication.

We therefore invite you to revise and resubmit your manuscript, along with a point-by-point response to the reviewers. Please highlight all changes in the manuscript text file.

Editorially, we consider it important that the revised manuscript addresses all conceptual (e.g., complexity) and methodological concerns raised by the reviewers. Please conduct additional robustness tests and correct for multiple comparisons.

We are committed to providing a fair and constructive peer-review process. Please don't hesitate to contact us if you wish to discuss the revision in more detail.

I am attaching an Editorial Requests Table that details critical reporting requirements for the revised manuscript. Please attend to each item and ensure your manuscript is fully compliant. If your revised manuscript is not aligned with these requests on major issues, such as those concerning statistics, it may be returned to you for further revisions without re-review.

Please submit the following items:

- Revised manuscript
- Point-by-point response to the referees' comments
- Cover letter (as a separate document)
- Nature Research Reporting Summary
- Completed Editorial Request Table (attached).

via this link: Link Redacted .

** This url links to your confidential home page and associated information about manuscripts you may have submitted or be reviewing for us. If you wish to forward this email to co-authors, please delete the link to your homepage first **

Additional guidance is available in our style and formatting guide Communications Psychology formatting guide.

We hope to receive your revised paper within 8 weeks; please let us know if you aren't able to submit it within this time so that we can discuss how best to proceed. If we don't hear from you, and the revision process takes significantly longer, we

may close your file. In this event, we will still be happy to reconsider your paper at a later date, provided it still presents a significant contribution to the literature at that stage.

We would appreciate it if you could keep us informed about an estimated timescale for resubmission, to facilitate our planning.

We look forward to seeing the revised manuscript and thank you for the opportunity to review your work.

Best regards,

Jennifer Bellingtier

Jennifer Bellingtier, PhD
Senior Editor
Communications Psychology

Troby Lui, PhD
Associate Editor
Communications Psychology

REVIEWER EXPERTISE:
Reviewer #1 metacognition/confidence, signal detection theory
Reviewer #2 metacognition/confidence, signal detection theory
Reviewer #3 metacognition/confidence, signal detection theory

REVIEWER REPORTS:

Reviewer #1 (Remarks to the Author):

Review of "Type-1 and Type-2 decisions feature computational noise of similar magnitude"
Reviewer: Michael Landy

This paper does a large-scale online perceptual decision-making experiment featuring both confidence and basic judgments and, for the latter, shifted criteria due to changing priors vs. payoffs. An interesting twist is that the prior/payoff is either stated prior to or after the stimulus display. They find shifted criteria (no surprise there), reduction in d' "due to" shifted criteria (apparently this is old news, but although sensible it contradicts my own paper that they cite, Locke et al. (2020), which assumes d' is defined by the stimuli, not the criterion, as in standard SDT). They fit a model (more on this below) that effectively treats criteria as noisy and the noise follows Weber's Law (the more you shift the criterion away from neutral, the noisier it gets) and finds somewhat comparable values of the Weber fraction (the log-normal sigma) for type-1 vs. type-2 judgments.

The paper is done well and I have no problem with the analysis (well, maybe some, but that's perhaps about clarity, see below). My overall complaints are (1) language and (2) scientific approach. By language I mean the use of the term "complexity", which shows up many times in the Introduction and Discussion, but is never defined and seems to have nothing to do with "complexity" in the sense of information (bits), minimal description length, or any other thing for which I might use that term. Here, a decision is "complex" to the extent that it has a larger criterion shift from neutral. Huh? Why is that "complex"? And why is a similar amount of shift for a first-order criterion and a 2nd-order criterion equally "complex"? As to scientific approach, the paper includes long stretches of reporting p-values and whatever for many pairs of conditions (for criterion shift, d' drop. criterion noise) without first stating a hypothesis and what they are looking for? It makes the results sections feel more like a data dump than a search for an answer to a hypothesis. The paper has the interesting manipulation of presenting the priors or payoffs before or after stimulus presentation. I can imagine a lot of reasons for trying this (and one, the DDM is discussed later on), but in the Introduction and Methods, there is no motivation given at all for doing this, and in Results, the differences or lack thereof are merely reported, rather than put in any context for why we'd want to know this or expect it to have (or not have) an effect. I can imagine that constructing a criterion AFTER you have the evidence might be noisier, for example, but you never say that.

Other comments (mostly by line number):

92: or the motivate -> or to motivate

109: "Biased cue" is not a good term because cue is easily associated with sensory evidence. “Biased context” might be better referred to as a condition with asymmetric prior or payoff.

Figure 1 and text nearby: Again, you should motivate why moving one type-I criterion to position c away from neutral is equally "complex" as moving TWO type-2 criteria the same distance. Sure, in your analysis you merge 3 bins in the confidence tasks to act as it there's only one type-2 criterion, but that's not what the observers are doing. Rather, I would drop the word "complex" entirely and state from the outset what you really mean. Going back to Green and Swets it's been

assumed that noisy evidence and a noisy criterion are equivalent (in the predicted data). However, you are working from the assumption that noisy criteria follow a kind of Weber's Law, where the noise is proportional to the "signal", i.e., to the amount of shift from neutral. So why not state this clearly in the Introduction and say that your question is whether that Weber's Law is evident in your data, and whether the Weber fraction (sigma_meta, basically) is the same for type-1 vs. type-2? That would make the paper much easier to read, IMHO.

You do cite my paper on this stuff (Locke et al., 2020, I mean), but you don't engage at all with some of the questions it raises. Your design and ours are nearly identical and the research question is similar, and yet you barely discuss it in Discussion and don't mention it in Intro as connected to your study, motivation for your study, or how your study differs. Are your criterion shifts by and large conservative (less than optimal shift), even for changed priors? You don't mention or deal with the issue that priors SHOULD change confidence criteria but that rewards shouldn't (even though, in our data, they do). There's no mention of conservatism vs. optimal criterion placement at all here, which seems odd. I expected contrasts between your results and ours (and others') in the Criterion Shift section of Results.

151: I expected detailed counts of numbers of subjects excluded based on each exclusion criterion here. The only exclusion counts show up in Results, and the number excluded (for negative d' values) seemed pretty enormous.

155-161. Did you tell participants where to look during stimulus presentation? Is 300 ms enough for a saccade, potentially, and is that an issue? You should note explicitly that you are using a 2IFC rather than a yes-no task, so the usual generalization of SDT to 2IFC applies.

173: Were these ranges of probability correct explicitly told to the subjets for making their binary confidence decisions?

189: Why would you tell them it's 3:1 and then have them experience 2:1? Don't you think the experienced prior will have an effect on their criterion and, in fact, possibly make that criterion shift across the block once they learn the true prior?

183 et seq: I felt that some details were left our. What was the text that indicated the cue on each trial (for priors or for rewards)? There are 50 trials in a block and 3 kinds of reward/prior setting. How many of each were run, since 50 is not multiple of 3 (or of 6, for that matter, including the actual stimulus)? Why was the confidence condition only 100 trials, when the type-1 conditions each got 150? Was feedback given on every trial, so the points were made explicit per trial?

202: Why at the end of the block instead of randomly placed catch trials, so that they wouldn't be somewhat predictable?

235: There is not Figure 1D.

257: I haven't heard the term "edge corrections", but folks lately mostly seem to use the Hautus (1995) correction, which is to add 0.5 trials to all 4 bins (CR, FA, Hit, Miss), which is biased, but not by much, rather than tossing the data, which doesn't seem reasonable.

292: Gussian -> Gaussian

294: This is really confusing. Why are you putting "c" into \mu? This is pretty non-standard (rather than acting like the means are symmetric around 0 for the 2IFC task) and you don't signal that you are going to do it nor why. Why shift the distributions of evidence rather than shift the criteria? It's equivalent, but trips up most readers.

296: distance BETWEEN THE distributions

307 et seq: This is hard to read. First, just clarify that you are assuming that criteria are noisy, that the noise never pushes the criterion to the other side of neutral, that this will be applied to both type-1 and type-2 criteria, and that it will be applied (mirror-flipped) to both positive (type-1 or type-2) criteria and to negative criteria. Just make it easy on your reader, rather than leaving all kinds of things tacit.

314-316: This is exceptionally hard to parse, especially "absolute value of the difference between confidence criteria or cue-biased decision criteria to the neutral...". I still can't parse it. Rather, log-normal is NOT applied to the difference between the right-hand and left-hand confidence criteria. It's applied separately to both (else there's a factor of 2 between the application to confidence vs. type-1, which would be problematic for your conclusion). CLARIFY. Note that sigma_meta is in the log domain, not in the raw evidence domain.

318: Once you add this log-normal noise, it means that the definitions of c and d' (lines 218 and 226) are now invalid estimators of d' and mean criterion. I can't tell from the manuscript, but I get the feeling that you DO define/estimate c and d' from those early, invalid definitions and then "fit" sigma_meta, leaving c and d' fixed. I'd think the only valid thing to do is use your full log-normal model (with parameters d', c, sigma_meta) and fit that to your data. Maybe that would be underconstrained (not enough data to fit the parameters well). But if you do the former, it's problematic, if not wrong. But, I can't tell from the manuscript what you actually did.

320: It's only here that I realized that given how you lumped the data for the confidence task, you are, in fact, treating confidence as involving only a single criterion just like the type-1 data. You might signal this more clearly earlier.

326: You say this is for the fixed "neutral" criterion, but your definition of mu_stim includes a non-neutral criterion, so that's

not true.

332: Here, I'm lost. Why are you subtracting q_1 from q_2 and why is that the right thing to do to predict your data? This all needs to be spelled out; I couldn't figure it out. We need more details about how the model is fit. Is \sigma_meta the only free parameter? Two stages fit together or separately? The original model paper Shekhar & Rahnev 2021 mentioned the issue of local minima that made them choose a nonconventional nested fitting procedure. Was that also the case?

394: It's not "much smaller" than pre-expectation

Figure 3B: y-axis should start at zero (or have a break if need be)

401: This says p=.06 but the graph says it's < .05 (an asterisk). But again, why are you making these comparisons? What's the hypothesis?

408: Again, it's worth pointing out that others (including Locke et al.) assume that d' is defined by the stimuli and NOT affected by rewards/priors. I presume you made that point in the earlier papers, but it can't hurt to make it again here.

410: the computational noise for the biased criteria manifests as: well, I'd say the noise is equivalent to a drop in d'. By the way why not scatterplot all the shifts vs. d' values to show the correlation?

Figure 4A: y-axis should start at zero (or at least show a break)

441: larger than the one -> larger than in 3 of the 4 cue conditions (or some such rephrase)

439-451: This is an example of a paragraph that feels like a data dump with lots of comparisons but no reference to impact on hypotheses you have nor what the results mean.

541: You might find our anti-DDM paper (Sun & Landy, 2016) relevant here. Or not. ;^)

Reviewer #2 (Remarks to the Author):

This is a very interesting article aiming to assess whether Type 1 and Type 2 decisions feature similar levels of computational noise once the complexity of the tasks is equated. I enjoyed reading the paper, particularly the clever design that manipulates complexity through confidence, cue, and reward conditions. The study addresses an important theoretical debate and is carefully executed.

I have the following comments and questions:

1) On the notion of complexity:
- Complexity is at the core of the paper’s argument, but it is not clearly defined. How can the complexity of a task be objectively assessed?
- Is complexity the only relevant construct? Alternative mechanisms (e.g., attention modulation by cues or rewards) could also explain the results.
- Why do reward manipulations not produce the same pattern as expectation cues, if the complexity is assumed to be equivalent across treatments?

2) Exclusion criteria could be better explained:
- A large number of exclusion criteria are applied. It would be helpful to know precisely how many participants were excluded under each criterion.
- Some participants are excluded from certain conditions but remain in others. Are the results robust if participants are excluded entirely once they fail in any one condition?

3) Some design choices should be explained / justified :
- Why was a 500 ms blank screen included in the confidence and post-cue conditions, while pre-cue conditions involved a 1500 ms cue?
- Why were there 100 confidence trials (2 blocks) but 150 cue/reward trials (3 blocks)?
- In post-cue conditions, why not require participants to view the cue/reward screen for 1500 ms after the stimulus presentation, matching the timing of pre-cue conditions?

4) It is not entirely clear how choices are reused to compute Type 1 SDT variables in the confidence condition. Are the same choices counted multiple times (e.g., once for “right,” once for “left,” and again for unbiased)? Clarification would help.

5) NaN values details are missing :
- How many subjects had NaN values for c and d’?
- How many required the modified formulas using only left or right data?
- Are results robust if these cases are excluded outright rather than adjusted?

6) Were any corrections applied for multiple comparisons in the pairwise contrasts between conditions? If not, this should be

acknowledged.

7) The criterion shift is consistently higher in the confidence condition compared to all others. I did not see an explicit explanation for this in the text.

8) The results sometimes show differences between pre- and post-cue manipulations (e.g., d’ drop for expectation). However, in the discussion, these manipulations are presented as similar. The discrepancy should be addressed more explicitly.

9) It would be useful to clarify what the computational model adds beyond the observed d’ drop. Much of the paper’s central claim relies on model-based estimates of noise, whereas the raw d’ drop results appear less clear.

10) The discussion of DDM implications may be too strong. Given that pre/post cue manipulations sometimes differ depending on the index used, the conclusions should be stated more cautiously.

11) The discussion on one vs. two systems and on potential common mechanisms could be further developed. Beyond the current findings, what empirical tests could distinguish these views? The section on DDM implications provides such concrete predictions, but the discussion of one vs. two systems is more general and could benefit from the same level of specificity.

Reviewer #3 (Remarks to the Author):

I co-reviewed this manuscript with one of the reviewers who provided the listed reports. This is part of the Communications Psychology initiative to facilitate training in peer review and to provide appropriate recognition for Early Career Researchers who co-review manuscripts.

* TRANSPARENT PEER REVIEW: Communications Psychology uses a transparent peer review system. This means that we publish the editorial decision letters including Reviewers' comments to the authors and the author rebuttal letters online as a supplementary peer review file. However, on author request, confidential information and data can be removed from the published reviewer reports and rebuttal letters prior to publication. If your manuscript has been previously reviewed at another journal, those Reviewers' comments would not form part of the published peer review file.

** Visit Nature Research's author and referees' website at www.nature.com/authors for information about policies, services and author benefits**

Communications Psychology is committed to improving transparency in authorship. As part of our efforts in this direction, we are now requesting that all authors identified as ‘corresponding author’ create and link their Open Researcher and Contributor Identifier (ORCID) with their account on the Manuscript Tracking System prior to acceptance. ORCID helps the scientific community achieve unambiguous attribution of all scholarly contributions. You can create and link your ORCID from the home page of the Manuscript Tracking System by clicking on ‘Modify my Springer Nature account’ and following the instructions in the link below. Please also inform all co-authors that they can add their ORCIDs to their accounts and that they must do so prior to acceptance.
https://www.springernature.com/gp/researchers/orcid/orcid-for-nature-research

For more information please visit http://www.springernature.com/orcid

If you experience problems in linking your ORCID, please contact the Platform Support Helpdesk.

Version 2:

Decision Letter:

** Please ensure you delete the link to your author homepage in this e-mail if you wish to forward it to your coauthors **

Dear Mr Zheng,

Your manuscript titled "Type-1 and Type-2 decisions feature computational noise of similar magnitude" has now been seen by our reviewers, whose comments appear below. In light of their advice I am delighted to say that we are happy, in principle, to publish a suitably revised version in Communications Psychology.

We therefore invite you to revise your paper one last time to address the remaining concerns of our reviewers and a list of editorial requests. At the same time we ask that you edit your manuscript to comply with our format requirements and to

maximise the accessibility and therefore the impact of your work.

EDITORIAL REQUESTS:

Please review our specific editorial comments and requests regarding your manuscript in the attached "Editorial Requests Table". Please outline your response to each request in the right hand column. Please upload the completed table with your manuscript files as a Related Manuscript file.

If you have any questions or concerns about any of our requests, please do not hesitate to contact me.

SUBMISSION INFORMATION:

In order to accept your paper, we require the files listed here https://www.nature.com/documents/commsj-file-checklist.pdf .

OPEN ACCESS:

Communications Psychology is a fully open access journal. Articles are made freely accessible on publication. For further information about article processing charges, open access funding, and advice and support from Nature Research, please visit https://www.nature.com/commspsychol/open-access

At acceptance, you will be provided with instructions for completing the open access licence agreement on behalf of all authors. This grants us the necessary permissions to publish your paper. Additionally, you will be asked to declare that all required third party permissions have been obtained, and to provide billing information in order to pay the article-processing charge (APC).

* TRANSPARENT PEER REVIEW: Communications Psychology uses a transparent peer review system. On author request, confidential information and data can be removed from the published reviewer reports and rebuttal letters prior to publication. If you are concerned about the release of confidential data, please let us know specifically what information you would like to have removed. Please note that we cannot incorporate redactions for any other reasons.

* CODE AVAILABILITY: All Communications Psychology manuscripts must include a section titled "Code Availability" at the end of the methods section. We require that the custom analysis code supporting your conclusions is made available in a publicly accessible repository at this stage; please choose a repository that generates a digital object identifier (DOI) for the code; the link to the repository and the DOI must be included in the Code Availability statement. Publication as Supplementary Information will not suffice.

* DATA AVAILABILITY:
All Communications Psychology manuscripts must include a section titled "Data Availability" at the end of the Methods section. More information on this policy, is available in the Editorial Requests Table and at http://www.nature.com/authors/policies/data/data-availability-statements-data-citations.pdf.

Please use the following link to submit the above items:
Link Redacted
** This url links to your confidential home page and associated information about manuscripts you may have submitted or be reviewing for us. If you wish to forward this email to co-authors, please delete the link to your homepage first **

We hope to hear from you within two weeks; please let us know if you need more time.

Best regards,

Troby Lui

Troby Lui, PhD
Associate Editor
Communications Psychology

REVIEWERS' COMMENTS:

Reviewer #1 (Remarks to the Author):

Re-review of "Type-1 and Type-2 decision feature computational noise of similar magnitude"
Reviewer: Michael Landy

This version is vastly clearer and more straightforward than the previous. I think my comments are few and all typos (page/para/line):

9/3/8: except exclusion criterion FOUR

Figure 2 legend: cues->cue (twice)

12/2/8: that -> than

13/1/3: conditions -> condition

21: first display equation has an extra ")" in the denominator

37/2/1: proportionate -> proportional

Reviewer #2 (Remarks to the Author):

The authors have addressed all of my queries, and I'm pleased with the revised manuscript.

Reviewer #3 (Remarks to the Author):

I co-reviewed this manuscript with one of the reviewers who provided the listed reports. This is part of the Communications Psychology initiative to facilitate training in peer review and to provide appropriate recognition for Early Career Researchers who co-review manuscripts.

** Visit Nature Research's author and referees' website at www.nature.com/authors for information about policies, services and author benefits**

Open Access This Peer Review File is licensed under a Creative Commons Attribution 4.0 International License, which permits use, sharing, adaptation, distribution and reproduction in any medium or format, as long as you give appropriate credit to the original author(s) and the source, provide a link to the Creative Commons license, and indicate if changes were made.
In cases where reviewers are anonymous, credit should be given to 'Anonymous Referee' and the source.

The images or other third party material in this Peer Review File are included in the article's Creative Commons license, unless indicated otherwise in a credit line to the material. If material is not included in the article's Creative Commons license and your intended use is not permitted by statutory regulation or exceeds the permitted use, you will need to obtain permission directly from the copyright holder.
To view a copy of this license, visit https://creativecommons.org/licenses/by/4.0/

Reviewer #1

Review of "Type-1 and Type-2 decisions feature computational noise of similar magnitude"
Reviewer: Michael Landy

This paper does a large-scale online perceptual decision-making experiment featuring both confidence and basic judgments and, for the latter, shifted criteria due to changing priors vs. payoffs. An interesting twist is that the prior/payoff is either stated prior to or after the stimulus display. They find shifted criteria (no surprise there), reduction in d' "due to" shifted criteria (apparently this is old news, but although sensible it contradicts my own paper that they cite, Locke et al. (2020), which assumes d' is defined by the stimuli, not the criterion, as in standard SDT). They fit a model (more on this below) that effectively treats criteria as noisy and the noise follows Weber's Law (the more you shift the criterion away from neutral, the noisier it gets) and finds somewhat comparable values of the Weber fraction (the log-normal sigma) for type-1 vs. type-2 judgments.

We thank the reviewer for the thoughtful and constructive comments. We appreciate your detailed summary of our design and findings, and we are glad that you found the paper well-executed overall.

The paper is done well and I have no problem with the analysis (well, maybe some, but that's perhaps about clarity, see below). My overall complaints are (1) language and (2) scientific approach. By language I mean the use of the term "complexity", which shows up many times in the Introduction and Discussion, but is never defined and seems to have nothing to do with "complexity" in the sense of information (bits), minimal description length, or any other thing for which I might use that term. Here, a decision is "complex" to the extent that it has a larger criterion shift from neutral. Huh? Why is that "complex"? And why is a similar amount of shift for a first-order criterion and a 2nd-order criterion equally "complex"?

We thank the reviewer for pointing this out. We agree. We meant the word “complexity” in a rather informal way but can see that this doesn’t work, especially when we say that decisions are matched on complexity. We have now removed the term complex/complexity and simply explain the issue as one of having to judge the strength of sensory evidence (in confidence judgments, as well as in the presence of priors or rewards) vs. having to make a simple categorization about which of two categories is associated with higher sensory evidence (in standard Type-1 judgments). We paste the updated Abstract below and have made equivalent changes throughout the paper:

Mounting evidence supports the existence of Type-2, metacognitive noise that affects human confidence judgments. The existence of this noise has led to the hypothesis that metacognitive judgments arise from a metacognitive system that is separate from the decision-making system responsible for Type-1 decisions. However, Type-2 decisions are different than standard Type-1 decisions in that they require evaluating the strength of sensory evidence rather than just making a simple categorical judgment. Here, we investigated whether Type-2 computational

noise still exceeds Type-1 computational noise when both judgments require evaluation of the strength of sensory evidence. Subjects (N=319) performed a perceptual discrimination task and in different conditions provided Type-2 confidence judgments or Type-1 judgments based on biased expectations or unequal rewards. All these judgments require a similar fine-grained evaluation of the strength of sensory evidence (e.g., is the evidence high enough to justify a high-confidence rating or choosing the alternative with smaller probability or reward). We first confirmed that the confidence and expectation conditions resulted in similarly biased criteria. Critically, both simple signal detection analyses and formal computational modeling confirmed that the confidence and expectation conditions exhibited comparable levels of computational noise. In contrast, the reward conditions exhibited both less biased criteria and smaller computational noise. These findings demonstrate that Type-1 and Type-2 decisions exhibit computational noise of similar magnitude. More generally, our results have implications for the debate on whether Type-1 and Type-2 decisions are made by separate systems.

As to scientific approach, the paper includes long stretches of reporting p-values and whatever for many pairs of conditions (for criterion shift, d' drop. criterion noise) without first stating a hypothesis and what they are looking for? It makes the results sections feel more like a data dump than a search for an answer to a hypothesis.

We also agree that many parts of the Results included long stretches of p-values. We have now thoroughly edited the Results section so that each type of analysis includes 3 distinct comparisons: Type-2 vs. Type-1 judgments, Pre-cues vs. post-cues, and Expectation vs. reward. Moreover, we introduce more clearly why these 3 specific comparisons are interesting in the Introduction section of the paper. We also no longer exhaustively report all pairwise comparisons (which led to the sense of data dump instead of a structured argument). Overall, this has made for a more structured Results section that should be easier to follow.

The paper has the interesting manipulation of presenting the priors or payoffs before or after stimulus presentation. I can imagine a lot of reasons for trying this (and one, the DDM is discussed later on), but in the Introduction and Methods, there is no motivation given at all for doing this, and in Results, the differences or lack thereof are merely reported, rather than put in any context for why we'd want to know this or expect it to have (or not have) an effect. I can imagine that constructing a criterion AFTER you have the evidence might be noisier, for example, but you never say that.

The reviewer is also correct that our manipulation of presenting cues before vs. after the stimulus was designed to allow inferences about the drift-diffusion model (DDM). In the experiment, we added a 500 ms blank screen after the stimulus in the pre-cue conditions to give participants time to integrate the cue, and for symmetry we also added a post-stimulus blank in the post-cue conditions. However, this design choice ultimately made reaction times during the decision phase inappropriate for fitting the DDM directly. For this reason, our main conclusions focused instead on computational noise estimates. We agree with the reviewer's point about clarifying this motivation, and we have now made it more explicit in the Introduction and Results sections. We paste the most relevant paragraphs below:

Introduction

One important consideration related to the computational noise in Type-1 judgments with expectation and reward cues is the timing of the cues. Specifically, such instruction cues are typically given before the sensory stimulus and are usually modeled as affecting either the starting point (Dekel & Sagi, 2020; Starns & Ratcliff, 2012; Summerfield & de Lange, 2014) or the drift rate (Afacan-Seref et al., 2018; Bottemanne & Dreher, 2019; Diaz et al., 2024; Sánchez-Fuenzalida et al., 2023; Summerfield & de Lange, 2014) of evidence accumulation. However, when a cue is presented after the sensory evidence (i.e., once evidence has already been accumulating for a while), it may have limited impact on the starting point or drift rate but instead affect the decision threshold or post-decisional evidence accumulation. In other words, because the cue's influence on evidence accumulation depends on its timing, the magnitude of computational noise may also vary with the temporal order between the cue and the sensory evidence. Type-2 confidence judgments are typically provided after the sensory decision and are sometimes modeled as being based on a post-decisional evidence accumulation (Calder-Travis et al., 2023; Desender et al., 2021; Pleskac & Busemeyer, 2010; van den Berg et al., 2016). Therefore, to comprehensively compare computational noise associated with expectation cues, reward cues, and confidence ratings, it is important to separately examine the effects of expectation and reward cues that appear before vs. after the stimulus (Bang & Rahnev, 2017).

Results

Criterion shift

Pre-cues vs post-cues

Prior research found that post-cues tend to result in larger biasing effects (Bang & Rahnev, 2017), which would mean larger criterion shifts in the current study. However, while our data showed a similar trend with post-cues producing numerically higher criterion shifts, there was little credible evidence that the criterion shift value differed between when the cue was presented before vs. after the stimulus in either the reward conditions ($t(189.84) = 1.89$, $p = .06$, $BF_{01} = 1.02$) or the expectation conditions ($t(184.27) = 1.78$, $p = .08$, $BF_{01} = 1.43$).

d' drop for biased criteria

Pre-cues vs post-cues

We found a small but significant effect where the degree of d' drop in the pre-expectation condition was larger than in the post-expectation ($t(231.07) = 2.41$, $p = .02$, $BF_{10} = 1.83$), but no such difference emerged when comparing the pre-reward and post-reward conditions ($t(208.93) = .92$, $p = .36$, $BF_{01} = 6.17$). Note that the pattern for d' drop is in the opposite direction than the pattern for criterion shift (Figure 3B), suggesting that the d' drop results are not driven by the criterion shift differences. Thus, presenting the cues before versus after the sensory evidence appears to induce higher computational noise in decision making, though this effect may depend on the type of cue.

Magnitude of computational noise

Pre-cues vs post-cues

There was no significant difference in the magnitude of computational noise between pre- and post-cues, with Bayes Factors indicating weak to moderate evidence for no difference (pre-expectation vs post-expectation: $t(151.13) = -1.21$, $p = 0.23$, $BF_{01} = 2.84$; pre-reward vs post-reward: $t(179.17) = 0.41$, $p = 0.68$, $BF_{01} = 7.36$). These results differ from the results in d' drop, which found a significant difference between the expectation conditions (though not between the reward conditions).

Other comments (mostly by line number):
92: or the motivate -> or to motivate

Corrected.

109: "Biased cue" is not a good term because cue is easily associated with sensory evidence. “Biased context” might be better referred to as a condition with asymmetric prior or payoff.

We agree with the reviewer that the word cue can be associated with the sensory evidence. We have therefore changed “overriding a biased cue” to “overriding the bias induced by the cue”.

Figure 1 and text nearby: Again, you should motivate why moving one type-I criterion to position c away from neutral is equally "complex" as moving TWO type-2 criteria the same distance. Sure, in your analysis you merge 3 bins in the confidence tasks to act as it there's only one type-2 criterion, but that's not what the observers are doing. Rather, I would drop the word "complex" entirely and state from the outset what you really mean. Going back to Green and Swets it's been assumed that noisy evidence and a noisy criterion are equivalent (in the predicted data). However, you are working from the assumption that noisy criteria follow a kind of Weber's Law, where the noise is proportional to the "signal", i.e., to the amount of shift from neutral. So why not state this clearly in the Introduction and say that your question is whether that Weber's Law is evident in your data, and whether the Weber fraction (sigma_meta, basically) is the same for type-1 vs. type-2? That would make the paper much easier to read, IMHO.

As mentioned above, we agree with the reviewer about the inappropriateness of the term “complexity” and have now removed it from the paper. We recognize that this framework resembles Weber’s law in that greater deviations from the neutral criterion are associated with greater variability in criterion placement. However, stating the issue in terms of Weber’s law is tricky in another sense. Specifically, we feel that talking about criterion noise in terms of Weber’s law could be seen as implying that even the noise for the standard Type-1 criterion (the natural criterion people use for discriminating between two stimulus categories) would differ depending on how close the criterion is to the place of intersection between the two Gaussian distributions in SDT. However, we don’t necessarily think that this is true. Rather, the intuition here is that *moving* the natural criterion is a noisy process. Because of this, we prefer not framing things in terms of Weber’s law. Instead, we frame the issue as one of having to judge the strength of sensory evidence (in confidence judgments, as well as in the presence of priors or rewards) vs. having to simply make a simple categorization about which of two

categories is associated with higher sensory evidence (in standard Type-1 judgments). We paste the updated text in and around Figure 1:

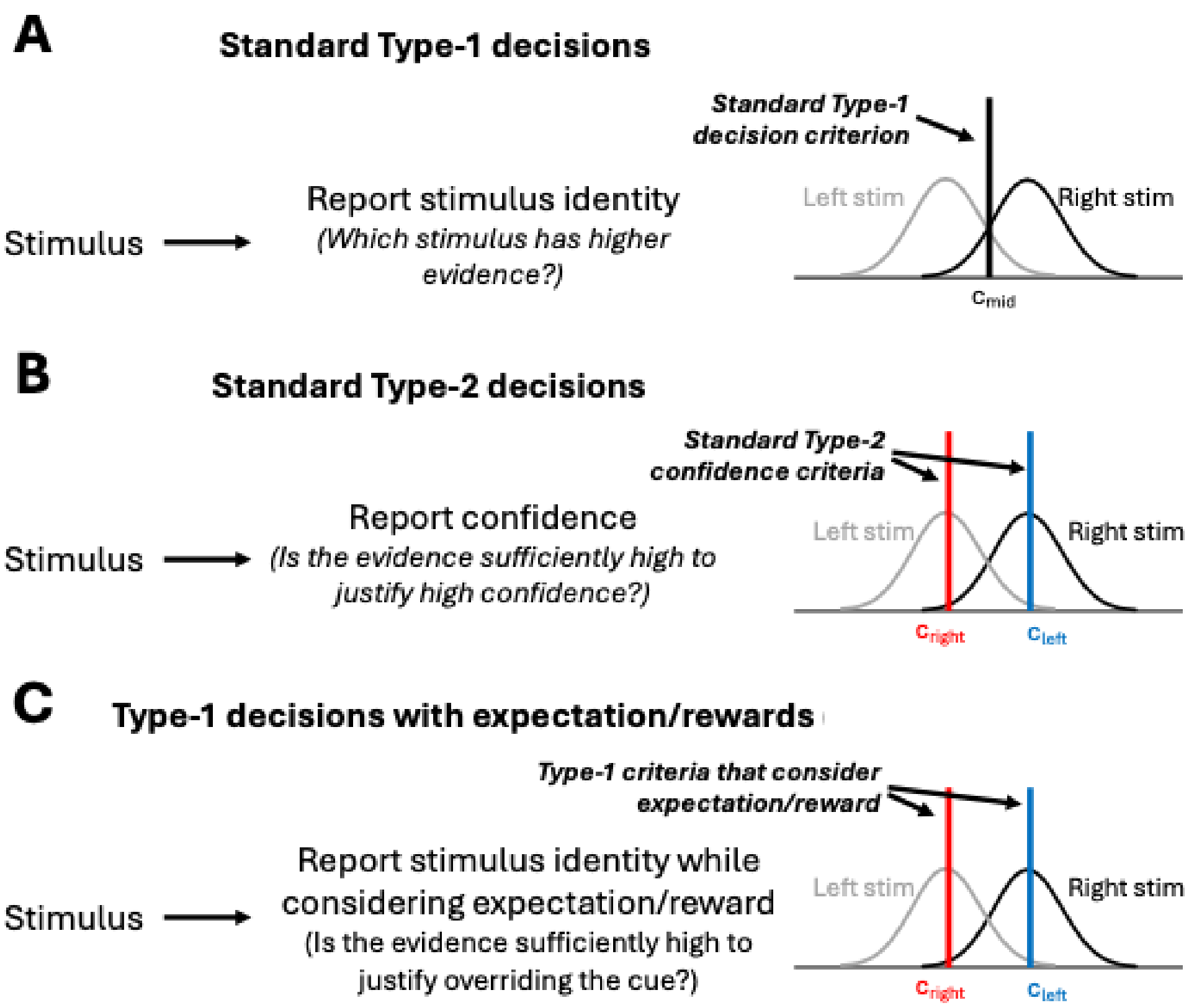

Figure 1. Evaluation of strength of sensory evidence in different kinds of Type-1 and Type-2 decisions*. A) Standard Type-1 decisions only require the individual to determine which category the stimulus belongs to. In practice, this usually means that a subject simply needs to select the category with higher evidence. Under the Signal Detection Theory (SDT) framework, this is equivalent to placing a decision criterion at the point where the two stimulus categories are equally likely (c_0). B) In contrast, Type-2 confidence judgments require a fine-grained evaluation of whether the strength of evidence is sufficient to justify high confidence. In the SDT framework, this is equivalent to placing confidence criteria (c_{-1} and c_1) in locations that are sufficiently shifted compared to c_0 as to justify giving a high confidence rating. C) Type-1 decisions with expectation or reward cues also involve fine-grained evaluation of the sensory evidence strength, as they require subjects to judge whether the strength of evidence is sufficient to justify overriding the bias induced by the expectation or reward cues. For example, overriding a cue that favors the "left" option is analogous to having high confidence that the "right" option is correct. In the SDT framework, this is equivalent to placing shifted decision criteria (c_{-1} and c_1) in potentially the same locations as the confidence criteria from panel B.*

You do cite my paper on this stuff (Locke et al., 2020, I mean), but you don't engage at all with some of the questions it raises. Your design and ours are nearly identical and the research question is similar, and yet you barely discuss it in Discussion and don't mention it in Intro as connected to your study, motivation for your study, or how your study differs. Are your

criterion shifts by and large conservative (less than optimal shift), even for changed priors? You don't mention or deal with the issue that priors SHOULD change confidence criteria but that rewards shouldn't (even though, in our data, they do). There's no mention of conservatism vs. optimal criterion placement at all here, which seems odd. I expected contrasts between your results and ours (and others') in the Criterion Shift section of Results.

We agree that Locke et al. (2020) is an important study in this space, and we appreciate you pushing us to engage with it more deeply. We initially didn't address the question of conservatism in criterion placement as we thought it was not directly relevant to the rest of our study, and we felt that it's a topic that has been investigated by a lot of previous papers. However, we can see that some engagement with this issue would be expected for a paper like this. So, now we discuss the issue of conservatism in criterion placement (which we replicate in our study). We show these results in the Criterion Shift section of Results and discuss how they relate to the findings in Locke et al. Additionally, we also revised the Discussion section to discuss the conservatism in both studies and link the findings in Locke et al. to explain why we found higher criterion shift in confidence condition than in other cue conditions. Further, we now engage more directly with Locke et al. (2020) in our Introduction. We paste the relevant sections from the Introduction, Results, and Discussion sections below:

Introduction

Several previous studies have examined how confidence is given in the presence of expectation or reward cues (Constant et al., 2023, 2025; Lebreton et al., 2019; Locke et al., 2020; Olawole-Scott & Yon, 2023; Sánchez-Fuenzalida et al., 2025; Sherman et al., 2016; West et al., 2025). Normatively, confidence should be influenced by expectation cues (which carry information about the stimulus) but not by reward cues (which do not)(Locke et al., 2020). Yet, several recent studies demonstrated that both types of cues influence confidence for most subjects (Lebreton et al., 2019; Locke et al., 2020; Sánchez-Fuenzalida et al., 2025). A related line of research has demonstrated that expectation cues have a larger effect on confidence than on the initial perceptual judgment (Constant et al., 2023, 2025). However, these studies examined how expectations and rewards influence confidence and did not explore whether computational noise is similar for Type-1 judgments biased by expectation or reward cues vs. standard Type-2 judgments (where confidence is given in the absence of biasing cues).

Results

We further tested whether criterion placement was conservative compared to the reward-maximizing ideal as found in prior studies (e.g., Locke et al., 2020; Maddox & Bohil, 2003; Rahnev & Denison, 2018). We found that all biased criteria (c_{-1} and c_1) were placed conservatively (i.e., not as extreme as the optimal) in all conditions (all p's < .028).

Discussion

Our study confirms and extends three findings from the literature. First, several previous studies have reported that d' associated with expectation cues is lower than d' associated with a neutral cue (what we call "d' drop"; Bang & Rahnev, 2017; de Lange et al., 2013; Haddara & Rahnev, 2024). Nevertheless, to the best of our knowledge, this d' drop has never been

explained in the context of a computational model or linked to the equivalent d' drop seen for confidence ratings (Shekhar & Rahnev, 2021b). Second, we also confirmed prior findings that reward cues have a smaller effect on criterion shift than expectation cues (Ackermann & Landy, 2015; Locke et al., 2020; Locke & Robinson, 2021; Maddox & Bohil, 1998, 2003). This finding is typically interpreted to imply that people prioritize accuracy over potential gains, hence showing greater reluctance to adjust their criteria in the presence of reward cues (Locke et al., 2020; Maddox & Bohil, 2003; Rahnev & Denison, 2018). Furthermore, we also found evidence that participants shifted their decision criterion not as extreme as the optimal, replicating previous findings of conservative criterion placement (e.g., Locke et al., 2020; Rahnev & Denison, 2018).

151: I expected detailed counts of numbers of subjects excluded based on each exclusion criterion here. The only exclusion counts show up in Results, and the number excluded (for negative d' values) seemed pretty enormous.

We thank the reviewer for highlighting this important point. We have now added a comprehensive list to the Participants section detailing the precise number of subjects excluded based on each criterion. The data of those subjects were used for the criterion shift and d' drop analyses. We paste the relevant section below:

After applying the exclusion criteria, the final number of subjects for each condition was: confidence (N=288), pre-expectation (N=301), post-expectation (N=296), pre-reward (N=273), and post-reward (N=257).

As noted, you correctly point out that a substantial number of participants were further excluded in the model-fitting stage because they exhibited at least one negative d' value under left-biased, right-biased, or neutral cue trials. These exclusions were necessary because negative d' values make the estimation of the noise magnitude unreliable. We realize that this leads to a large number of exclusions, but given the large initial sample size, we feel that the results are more meaningful if we can obtain reliable noise magnitude estimates even if that means excluding more subjects. We now clarify this and paste the relevant parts here:

To ensure reliable model fitting estimates, we further excluded subjects from the criterion shift and d' drop analysis and only conditions with a positive d' across all three cue types were included, resulting in valid data from 155, 126, 123, 151, and 155 subjects for confidence, pre-expectation, post-expectation, pre-reward, and post-reward conditions, respectively.

Finally, we also performed the criterion shift and d' drop analyses on the subset of subjects whose data were included in the model fitting. We found qualitatively similar results, and added the results in supplementary Figure S7, which is also pasted below:

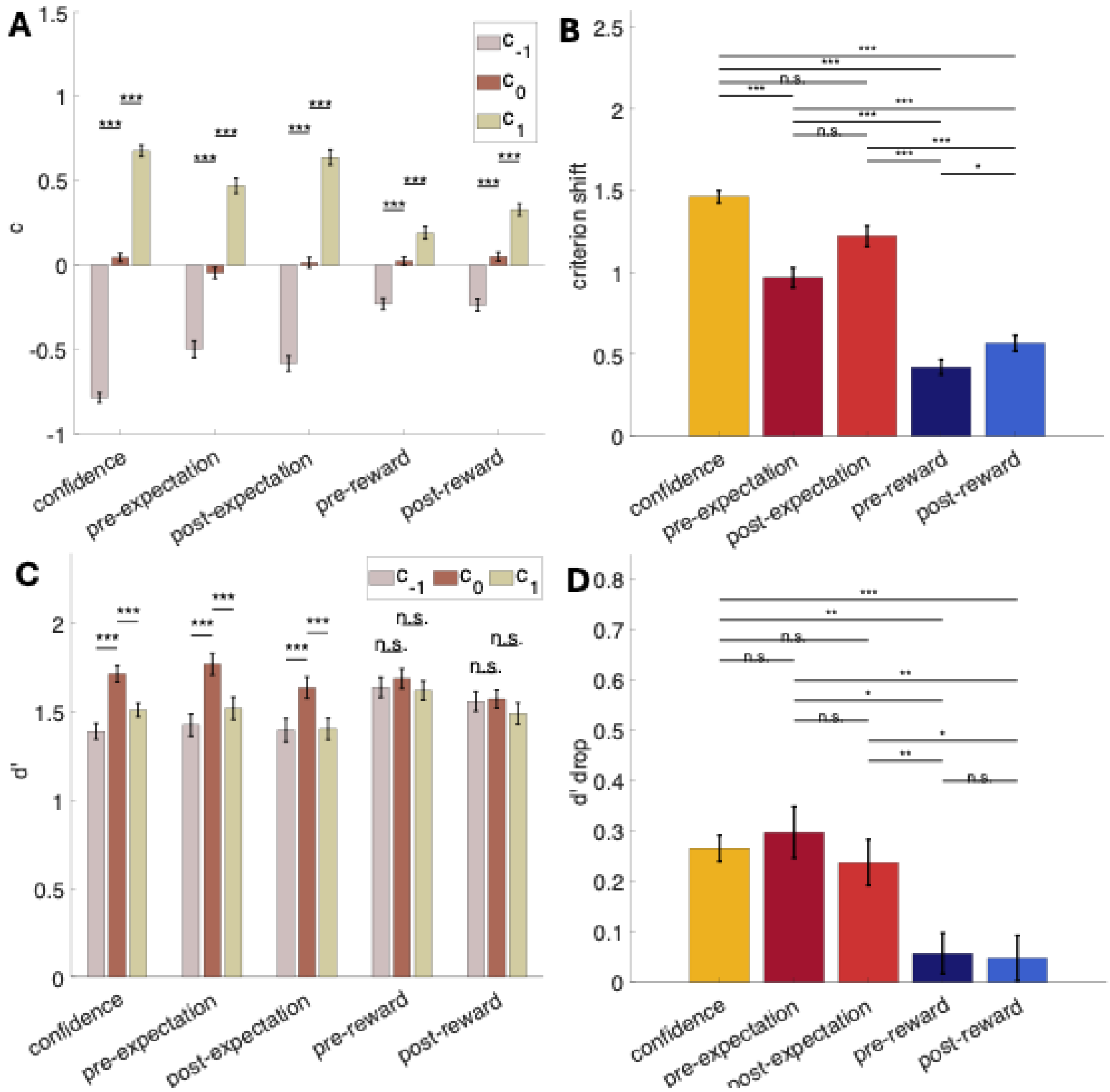

__Figure S7. Replication with the subset of subjects used in model fitting.__ In the model fitting procedure, we included a subset of 155, 126, 123, 151, and 155 subjects for confidence, pre-expectation, post-expectation, pre-reward, and post-reward conditions. Notably, these subjects were those whose data did not need the modified formulas when computing criterion shift and d' drop. Here we examined whether we could replicate the main findings on those subjects. A) Criterion location in each condition for the three criterion locations (c_{-1}, c_0, c_1). This pattern is identical to the main analyses (Figure 3A). B) The degree of criterion shift for each condition. The pattern roughly replicated the one in main analyses. However, there is no significant difference between confidence condition and post-expectation conditions ($p = 0.125$), while the difference between pre-reward and post-reward conditions was significant ($p = 0.04$). C) d' values associated with three criterion locations (c_{-1}, c_0, c_1) in each condition. The pattern replicated the one in main analyses, except that the d' for biased criteria was not significantly lower than the one for unbiased criteria in both reward conditions (all p's > 0.128). D) The degree of d' drop for each condition. The pattern was identical to that observed in the main analyses (Figure 4B), such that the d' drop in the confidence and expectation conditions was greater than that in the reward conditions. However, there was no significant difference between the confidence and post-expectation conditions ($p = 0.96$), nor between the pre-expectation and post-

*expectation conditions (p = 0.167). In addition, we observed a significant difference between the post-expectation and post-reward conditions (p = 0.012). Overall, these results were in line with the main manuscript's findings. Error bars show SEM. ***, p < .001; **, p < .01; *, p < .05; n.s., not significant.*

155-161. Did you tell participants where to look during stimulus presentation? Is 300 ms enough for a saccade, potentially, and is that an issue? You should note explicitly that you are using a 2IFC rather than a yes-no task, so the usual generalization of SDT to 2IFC applies.

We thank the reviewer for this comment. A fixation cross was presented at the center of the screen during both the fixation and stimulus phases to guide participants' gaze. We note that this was a comparison task where subjects had to determine which of two stimuli had more dots. Thus, making a saccade toward one of the targets would not be helpful as both targets have to be processed in order for subjects to complete the task. We have also clarified in the beginning of the Statistical analysis section that the design was a 2AFC comparison task, and that the standard SDT generalization to 2AFC therefore applies. We paste the relevant text below:

The experiment used a two-alternative forced-choice (2AFC) design. Signal Detection Theory (SDT) generalizes straightforwardly to 2AFC by modeling the decision as a comparison of two internal responses, with the higher response determining the choice.

173: Were these ranges of probability correct explicitly told to the subjets for making their binary confidence decisions?

Yes. Participants were explicitly instructed on the screen that their binary confidence responses should correspond to either a 50–75% or a 75–100% probability of being correct. We now further clarified this issue in the procedure section and pasted below too:

Immediately after, they reported their confidence level on a binary scale: low confidence (i.e., 50-75% probability of being correct) or high confidence (75-100% probability of being correct). These confidence ranges were displayed on the screen during the confidence report to remind participants of the intended mapping.

189: Why would you tell them it's 3:1 and then have them experience 2:1? Don't you think the experienced prior will have an effect on their criterion and, in fact, possibly make that criterion shift across the block once they learn the true prior?

We appreciate the reviewer's concern. Our design involved a tradeoff between experiment length and the number of trials with invalid expectation cues. To limit the session to about one hour and reduce fatigue in online participants, we set 50 trials for each type of cue (left, right, and neutral), which we found reasonable in pilot testing. Within those 50 trials, using a true 3:1 ratio would have yielded too few invalid-cue trials (13 trials), so we implemented a roughly 2:1

(33:17) ratio instead. We believe the impact of participants learning the experienced prior was minimal, as no Type-1 feedback was provided.

To further demonstrate the learning effect is minimal, we performed a split-half analysis on the expectation conditions. Paired t-tests compared the decision criterion for left-cued and right-cued trials between the first and second halves of the condition. These tests revealed no significant change in both right-biased and left-biased criterion in either the pre- or post-expectation conditions, suggesting that learning effects were minimal. We included this part of results in Figure S3, and pasted the relevant text and figure below:

Methods

In reality, the biased expectation cues were valid on 66% of the trials to ensure that we could collect sufficient number of trials with invalid cues. Subjects did not seem to be affected by this slight mismatch between the instruction and true validity rate as their criterion placement was identical between the first and second halves of the experiment (Figure S3).

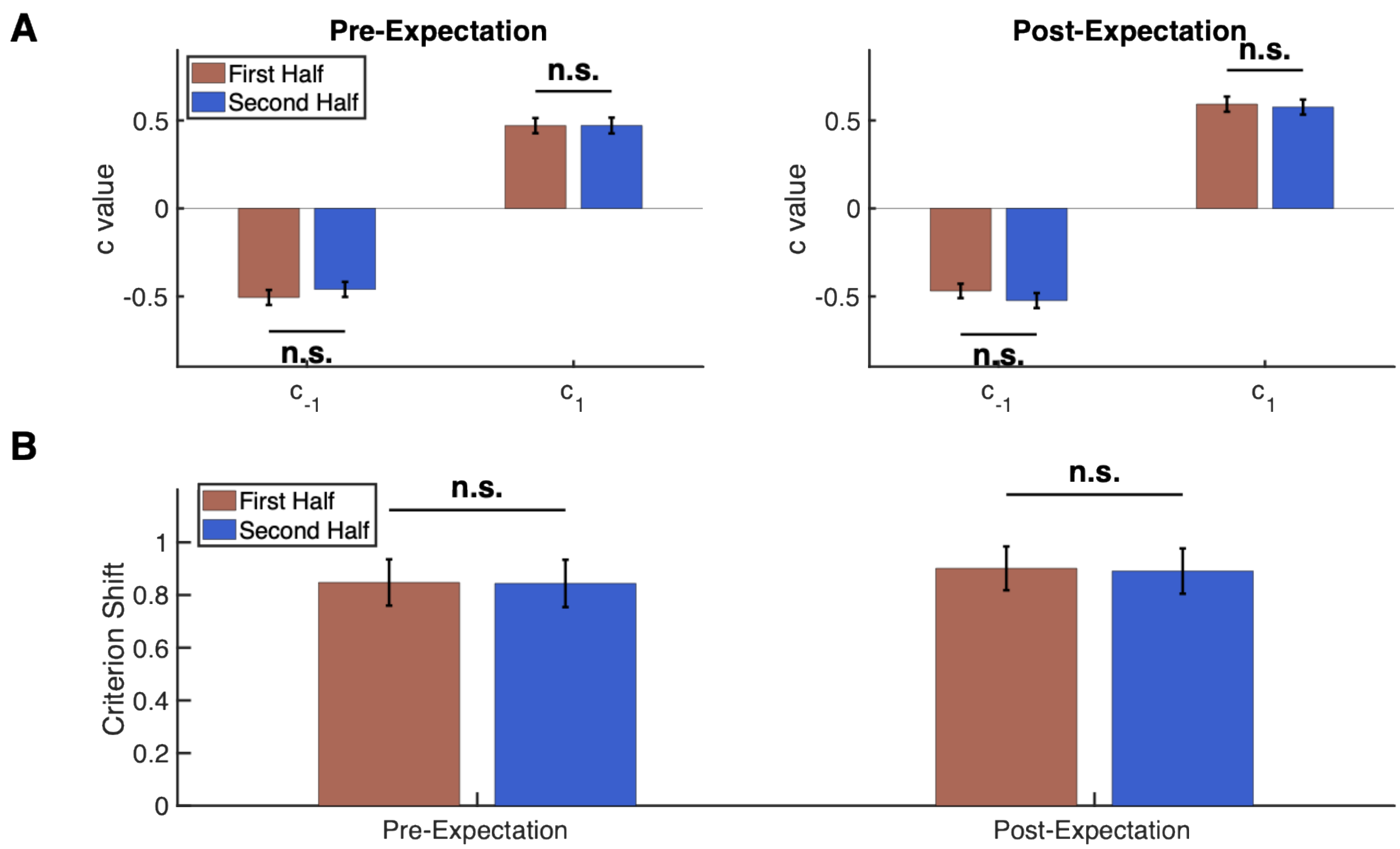

Figure S3. Criterion placement stays the same between first and second half in the expectation conditions*. The instructions regarding the expectation cues stated that one choice was three times more likely to be correct (i.e., implied cue validity was 75%). However, in reality, the true cue validity was less extreme (66%), which allowed for more trials with invalid cues. It might be possible that subjects learned the underlying probabilistic structure over time and subsequently reduced their reliance on the expectation cues. To test this hypothesis, we conducted paired t-tests comparing the decision criterion for left-cued and right-cued trials between the first and second halves of each expectation condition. A) We found no significant*

changes in either c_{-1} or c_1 across both the pre-expectation and post-expectation conditions (all p's > .18, all BF_{01} > 5.91). B) Further, the criterion shift value between first and second half of trials were also similar in the pre-expectation and post-expectation conditions (p's > .11, BF_{01} > 4.15). Taken together, these results indicate strong evidence against the notion that subjects learned the true cue validity and thereby shifted their decision criterion more conservatively.

183 et seq: I felt that some details were left our. What was the text that indicated the cue on each trial (for priors or for rewards)? There are 50 trials in a block and 3 kinds of reward/prior setting. How many of each were run, since 50 is not multiple of 3 (or of 6, for that matter, including the actual stimulus)? Why was the confidence condition only 100 trials, when the type-1 conditions each got 150? Was feedback given on every trial, so the points were made explicit per trial?

We thank the reviewer for pointing out the need to clarify these points. We explain each of these in turn.

<u>Specific text for each cue</u>
The reward cues specified either unbiased payoffs (1 point for left or right choices, if the choice was correct) or biased payoffs (3 points for one choice, 1 point for the other, if the choice was correct). The expectation cues specified either unbiased priors (equal probability for left and right choices) or biased priors (one option three times more likely than the other).

The reward conditions contained biased and unbiased reward cues. The unbiased reward cues indicated that correct left and right choices would both result in winning 1 point, whereas the biased reward cues indicated that one of the choices would win 3 points. The cue was presented by indicating the number of points associated with each stimulus (either "1 point" or "3 points") above each stimulus location. The expectation conditions similarly contained biased and unbiased expectation cues. The unbiased expectation cue indicated that the left and right options were equally likely ("two sides equally likely" on the screen), whereas the biased expectation cues indicated that one of the choices was 3 times more likely that the other (e.g., "Left is 3 times more likely" on the screen).

<u>Trial counts of 50 and 3 kinds of blocks</u>
Each cue condition (e.g., pre-expectation or post-reward) consisted of 150 trials (50 left-biased, 50 right-biased, 50 neutral). These 150 trials were shown in three blocks of 50 trials where the three types of cue (left-biased, right-biased, and neutral) were randomly interleaved.

Within these conditions, the 150 total trials per condition were evenly divided into left-biased, right-biased, and neutral cues, and were shown in three blocks of 50 trials where the three cue types were randomly interleaved.

<u>Confidence condition having only 100 trials</u>
The confidence condition included 100 trials in total. This is because the confidence condition is more efficient with all 100 trials contributing to the estimation of all 3 criteria (the decision and

the two confidence criteria). In contrast, each of the three cue types (left-biased, right-biased, and unbiased) in the cue conditions only contributed to the estimation of a single criterion. Thus, while the confidence condition had fewer total number of trials, it actually provided higher precision in the estimation of the criteria. We now clarify this in the Methods:

The confidence condition then consisted of two blocks of 50 trials, with a total of 100 trials. The trial number in the confidence condition was lower than each of the four cue conditions. This is because in the cue conditions, each type of cue only contributed to the estimation of a single criterion (the unbiased decision criterion and the two biased criteria), while 100 trials in the confidence condition contributed to the estimation of all 3 criteria.

<u>Feedback structure</u>
Feedback was not provided on each trial; instead, participants were only informed of their cumulative points at the end of the experiment. We have clarified this in the procedure and pasted below:

Notably, in all conditions, subjects were told that correct responses would allow them to accumulate points across the experiment; however, Type-1 decision feedback was not provided at the end of each trial in any of the conditions. In the end of the experiment, subjects were given a bonus based on the total number of points earned.

202: Why at the end of the block instead of randomly placed catch trials, so that they wouldn't be somewhat predictable?

We thank the reviewer for pointing this out. We have corrected the description to note that the attention check was placed at the end of each condition rather than each block. We agree that randomly placed attention checks would generally be more effective. The reason why we didn't want to give them in the middle of a block is that we were worried that serial dependence and other possible adjustments of strategy could affect the following trials. Further, given the number of trials per condition and the fact that participants were not informed when a condition would end before an attention check, inattentive participants making random responses would still be likely to miss the attention check. Thus, although somewhat predictable, our checks still provided a useful measure of participants' attentional state during the task. We have now briefly discussed this reasoning:

The attention checks were not randomly added in the middle of a block because we concerned that serial dependence or other possible adjustments of strategy might affect the following trials. Further, given the number of trials per condition and the fact that participants were not informed when a condition would end before an attention check, inattentive participants would still be likely to miss the attention check if they just made random responses.

235: There is not Figure 1D.

Corrected.

257: I haven't heard the term "edge corrections", but folks lately mostly seem to use the Hautus (1995) correction, which is to add 0.5 trials to all 4 bins (CR, FA, Hit, Miss), which is biased, but not by much, rather than tossing the data, which doesn't seem reasonable.

The “edge correction” we referred to follows the same logic as the Hautus (1995) procedure, namely adding 0.5 to all four cells (Hits, Misses, CRs, FAs) to prevent infinite z-scores when empty bins occur. However, as shown by Shekhar & Rahnev (2021), such corrections can introduce systematic biases. In particular, inflating false alarm rates leads to underestimation of d’. Because our analyses rely on accurately estimating d’ across trial types and using the d’ drop as a key measure of computational noise, we chose not to apply this correction, despite the reduction in the number of usable data points.

Nevertheless, we now repeated the analyses after applying the edge correction and obtained the same qualitative pattern. We now made the clarifications in the main text and report these additional analyses in Figure S4, which we paste below:

One common method of addressing this issue is by applying the Hautus’s correction (1995) to avoid the zero hit rare and false alarm rate (Macmillan & Creelman, 2004).

Notably, we found qualitatively similar results when applying the Hautus’s edge correction and not using the modified formulas to compute criterion shift and d’ drop (Figure S4).

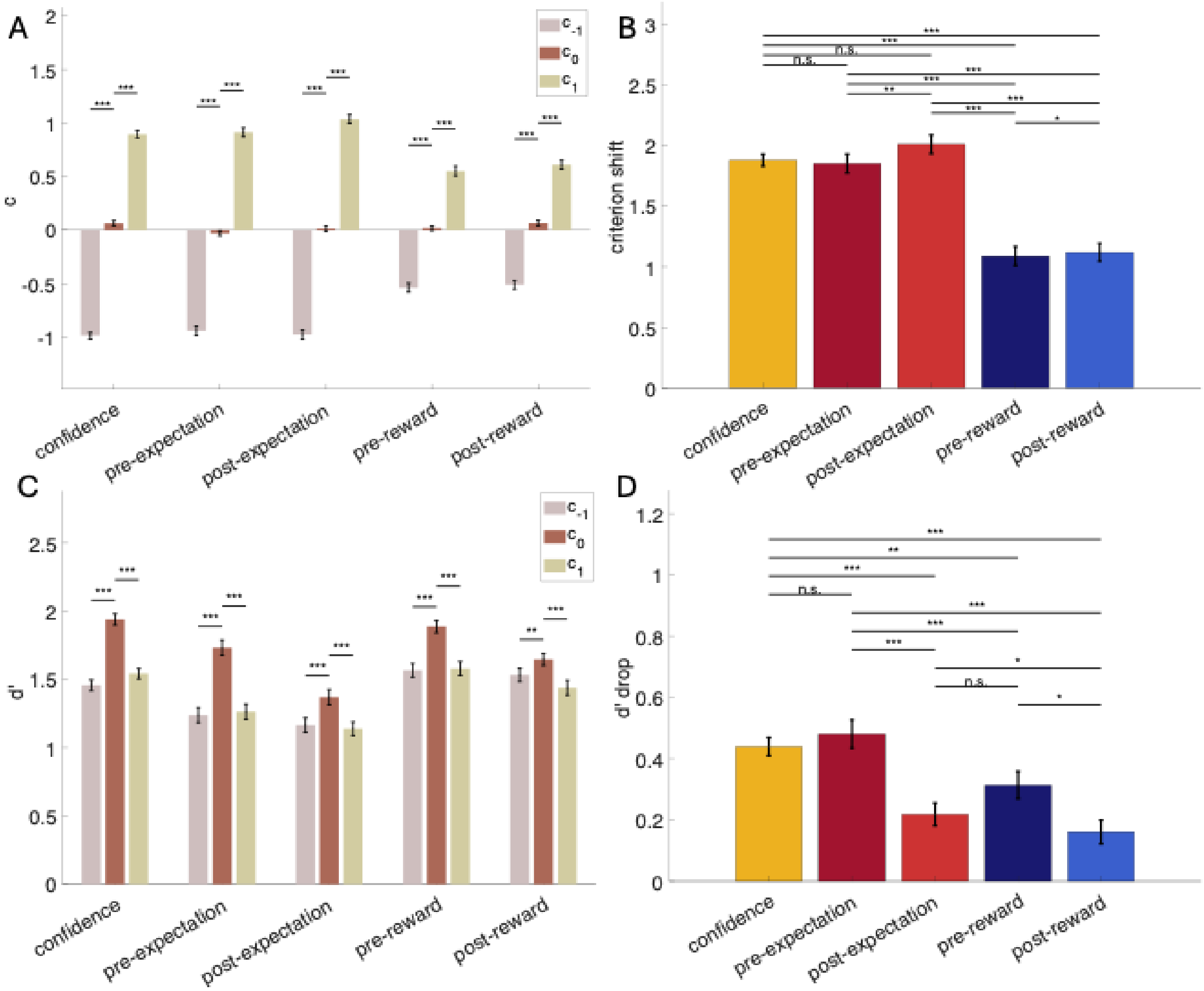

***Figure S4. Results with applying edge correction**. Here, we followed standard Signal Detection Theory (SDT) analysis and applied padding to zero response counts to avoid zero hit rates and false alarm rates when estimating c and d' values. The results were similar to those in the main manuscript (Figures 3 and 4). A) Criterion location in each condition for the three criterion locations (c_{-1}, c_0, c_1). This pattern is identical to the main analyses (Figure 3A). B) The degree of criterion shift for each condition. The pattern roughly replicated the one in main analyses, such that reward conditions had smaller criterion shift. However, there is no significant difference between confidence condition and two expectation conditions (p = 0.32 and 0.45 for pre-expectation and post-expectation conditions, respectively). Furthermore, post-cue conditions had significantly greater criterion shift than pre-cue conditions (pre-expectation vs. post-expectation, p = 0.005; pre-reward vs post-reward, p = 0.033). C) d' values associated with three criterion locations (c_{-1}, c_0, c_1) in each condition. The pattern is identical to the main analyses (Figure 4A), except that the d' for c_0 was significantly higher than c_{-1} (p = 0.007). D) The degree of d' drop for each condition. The pattern is identical to the main analyses (Figure 4B) so that confidence and pre-expectation conditions had similar degree of d' drop and they were higher than the ones in other conditions. However, the d' drop in the post-expectation condition was significantly greater than in the post-reward condition (p = 0.031), and the d' drop in the pre-*

*reward condition was also significantly greater than in the post-reward condition (p = .030). Overall, these results were in line with the main manuscript's findings. Error bars show SEM. ***, p < .001; **, p < .01; *, p < .05; n.s., not significant.*

292: Gussian -> Gaussian

Corrected.

294: This is really confusing. Why are you putting "c" into \mu? This is pretty non-standard (rather than acting like the means are symmetric around 0 for the 2IFC task) and you don't signal that you are going to do it nor why. Why shift the distributions of evidence rather than shift the criteria? It's equivalent, but trips up most readers.

Thank you for this comment. We wrote it this way to correspond to the way this was coded in our scripts, but we recognize that this is more complex and non-standard way to talk about it in the Methods. We have therefore removed the decision criterion from the \mu. We paste the updated section below:

Following standard SDT assumptions, we assume that each stimulus category generates noisy sensory evidence, x, drawn from Gaussian distributions. For stimuli from category S_1 (i.e., the left square containing more dots in this case), the evidence is drawn from $x \sim N(-\frac{\mu_{sens}}{2}, \sigma_{sens}^2)$ and for stimuli from category S_2 (i.e., the right square containing more dots), evidence is drawn from $x \sim N(\frac{\mu_{sens}}{2}, \sigma_{sens}^2)$ where μ_{sens} represents the distance between the two evidence distributions and σ_{sens} is the standard deviation of the distributions (set to 1 without loss of generality).

296: distance BETWEEN THE distributions

Corrected.

307 et seq: This is hard to read. First, just clarify that you are assuming that criteria are noisy, that the noise never pushes the criterion to the other side of neutral, that this will be applied to both type-1 and type-2 criteria, and that it will be applied (mirror-flipped) to both positive (type-1 or type-2) criteria and to negative criteria. Just make it easy on your reader, rather than leaving all kinds of things tacit.
314-316: This is exceptionally hard to parse, especially "absolute value of the difference between confidence criteria or cue-biased decision criteria to the neutral...". I still can't parse it. Rather, log-normal is NOT applied to the difference between the right-hand and left-hand confidence criteria. It's applied separately to both (else there's a factor of 2 between the application to confidence vs. type-1, which would be problematic for your conclusion). CLARIFY. Note that sigma_meta is in the log domain, not in the raw evidence domain.

We thank the reviewer for pointing out the lack of clarity in this section. To clarify, the lognormal noise is applied separately to each individual criterion, whether a type-1 criterion under biased cues or a type-2 confidence criterion, relative to the neutral decision criterion. It is not applied to the difference between left- and right-hand criteria. This avoids any asymmetry or scaling issues between type-1 and type-2 cases. Furthermore, we have clarified in the text that σ_{meta} (what we now call σ_{comp} to clarify that this doesn't apply just to confidence ratings but also to the biased Type-1 criteria) denotes variability in the log domain, as in Shekhar & Rahnev (2021b), and not in the raw evidence domain. We revised this section to state our model assumptions and the detailed methods more explicitly so that readers can follow the logic more easily:

Confidence judgements in the current study were generated using a set of confidence criteria $[c_{-1}, c_1]$. The key assumption of the lognormal meta noise model (Shekhar & Rahnev, 2021b) is that these confidence criteria are not fixed but vary from trial to trial according to a lognormal distribution. This variability represents metacognitive noise: sources of uncertainty that selectively affect confidence but not the primary decision. The lognormal meta noise model can be extended to the Type-1 criteria biased by expectation and reward instruction cues. The formulas below apply to c_{-1} and c_1 regardless of condition (confidence vs. cue conditions).

The criteria c_i follow a lognormal probability distribution, g, though the formulas for c_{-1} and c_1 are slightly different since they fall on opposite sides of the decision criterion. Specifically, c_1 follows the following distribution:

$$c_1 \sim g(y \mid \mu_1, \sigma_{comp}^2) = \frac{1}{(y - c_0))\sqrt{2\pi\sigma_{meta}^2}} exp(-\frac{(ln(y - c_0)) - \mu_1)^2}{2\sigma_{meta}^2}), y \in (c_0, \infty)$$

and c_{-1} follows the following distribution:

$$c_{-1} \sim g(y \mid \mu_{-1}, \sigma_{comp}^2) = \frac{1}{(c_0 - y)\sqrt{2\pi\sigma_{meta}^2}} exp(-\frac{(ln(c_0 - y) + \mu_{-1})^2}{2\sigma_{meta}^2}), y \in (-\infty, c_0)$$

where μ_{-1} and μ_1 are the means of the Gaussian random variable obtained by taking log of c_{-1} and c_1 (with the constraint that $\mu_{-1} < c_0 < \mu_1$), and σ_{comp} is the standard deviation of this random variable, which serves as a measure of the amount of computational noise. Note that σ_{comp} quantifies the amount of computational noise in the log domain, not in the raw evidence domain.

318: Once you add this log-normal noise, it means that the definitions of c and d' (lines 218 and 226) are now invalid estimators of d' and mean criterion. I can't tell from the manuscript, but I get the feeling that you DO define/estimate c and d' from those early, invalid definitions and then "fit" sigma_meta, leaving c and d' fixed. I'd think the only valid thing to do is use your full log-normal model (with parameters d', c, sigma_meta) and fit that to your data. Maybe that would be underconstrained (not enough data to fit the parameters well). But if you do the former, it's problematic, if not wrong. But, I can't tell from the manuscript what you actually did.

320: It's only here that I realized that given how you lumped the data for the confidence task, you are, in fact, treating confidence as involving only a single criterion just like the type-1 data. You might signal this more clearly earlier.
326: You say this is for the fixed "neutral" criterion, but your definition of mu_stim includes a non-neutral criterion, so that's not true.
332: Here, I'm lost. Why are you subtracting q_1 from q_2 and why is that the right thing to do to predict your data? This all needs to be spelled out; I couldn't figure it out. We need more details about how the model is fit. Is \sigma_meta the only free parameter? Two stages fit together or separately? The original model paper Shekhar & Rahnev 2021 mentioned the issue of local minima that made them choose a nonconventional nested fitting procedure. Was that also the case?

We thank the reviewer for the constructive feedback on our modeling methods. These comments have helped us realize that our description of the model fitting was confusing and incomplete. We have now substantially revised the Methods section to provide a clear, accurate, and detailed account of our procedure. As we mentioned above, to avoid confusion, we renamed the parameter σ_{meta} to σ_{comp}.

Specifically, we have made the following clarifications in response to your points:

1. On the fitting procedure (Line 318 & 332): You are correct that standard SDT formulas for d' and c are not unbiased estimators once trial-by-trial noise is introduced, and that fitting only the noise parameter would be problematic. We apologize for the lack of clarity that gave this impression. In fact, our procedure was to perform a single-stage, joint optimization of all model parameters: sensitivity (μ_{sens}), the neutral criterion (c), the confidence or biased criteria (c_{-1} and c_1), and the computational noise (σ_{comp}). The standard SDT-derived values were used solely as empirically-grounded starting points for the optimization algorithm (BADS). We didn't use the 2-stage procedure from Shekhar & Rahnev (2021) since BADS allowed good fitting by jointly optimizing all free parameters (in Shekhar & Rahnev we didn't use BADS, which is why we had to resort to the 2-stage procedure).

   To fit the model, we employed the Bayesian Adaptive Direct Search (BADS) optimization algorithm (Acerbi & Ma, 2017) to jointly fit all 5 free parameters: sensitivity, μ_{sens}; the neutral criterion, c_0; the means of the criteria c_{-1} and c_1 (i.e., parameters μ_{-1} and μ_1); and the computational noise, σ_{comp}. Fitting was performed by minimizing the negative log-likelihood of the data. To ensure a robust fitting process, the algorithm was initialized with parameter values of μ_{sens}, c_0, μ_{-1}, and μ_1 derived from standard SDT calculations, while σ_{comp} was initialized to the value of 0.05.

2. On calculating response probabilities (Line 332): You correctly noted that the phrase "subtracting the cumulative probabilities q1 and q2" was an inaccurate and confusing simplification of the full model. We have revised the incomplete descriptions with a clear explanation of the response probability calculation process (see pasted text under point 3).

3. On the μ_{stim} definition (Line 326): We appreciate you pointing out the confusing wording regarding the neutral criterion. Our model defines the sensory evidence mean (which we now call μ_s instead of μ_{stim}) relative to the decision axis's origin (zero), while the neutral criterion, c, is a free parameter on that axis. A response is made based on whether the evidence falls to one side of c.

   We paste the updated section on the response probability calculation below:

   To compute the log-likelihood of the data, the model must determine the probability of a subject's 'Left' or 'Right' response given a specific stimulus (S_1 or S_2) and criterion (c_{-1}, c_0, c_1). The formulas vary based on the criterion but are the same regardless of whether stimulus S_1 or S_2 was presented. Below, we give the probabilities of providing the 'Left' response; the probability of responding 'Right' is simply 1 minus the probability of responding 'Left'.

   For the fixed neutral criterion c_0, the probability of a 'Left' response given stimulus S is equal to the probability that the sensory evidence x is less than c_0:

   $$P(x < c_0 \mid S) = \Phi(c_0 \mid \mu_s, \sigma_{sens}^2)$$

   where Φ is the cumulative normal distribution, μ_s is the mean of the stimulus S distribution ($\mu_s = -\frac{\mu_{sens}}{2}$ if $S = S_1$ and $\mu_s = \frac{\mu_{sens}}{2}$ if $S = S_2$), and $\sigma_{sens} = 1$ is the standard deviation of this distribution.

   The formulas are more complex for the noisy criteria c_{-1} and c_1. In these cases, the probability of a 'Left' response equals probability that the sensory evidence r is less than these criteria, which are themselves described by the distributions $y \sim g(\mu_{-1}, \sigma_{comp}^2)$ and $y \sim g(\mu_1, \sigma_{comp}^2)$. Thus, the formula for the probability of a 'Left' response for criterion c_{-1} becomes:

   $$P(x < g(y \mid \mu_{-1}, \sigma_{comp}^2) \mid S) = \int_{x=-\infty}^{c_0} \int_{y=x}^{c_0} \Phi(x \mid \mu_s, \sigma_{sens}^2)\, g(y \mid \mu_{-1}, \sigma_{comp}^2)\, dy\, dx$$

   In the case of criterion c_1, it is simpler to give the formula for the probability of a 'Right' response (the probability of responding 'Left' is simply 1 minus the probability of responding 'Right'), which is:

   $$P(x > g(y \mid \mu_1, \sigma_{comp}^2) \mid S) = \int_{x=c_0}^{\infty} \int_{y=c_0}^{x} \Phi(x \mid \mu_s, \sigma_{sens}^2)\, g(y \mid \mu_1, \sigma_{comp}^2)\, dy\, dx$$

4. On signposting the confidence data analysis (Line 320): This is a helpful suggestion. To improve clarity, we have added a sentence to the "Statistical analysis" section to more

explicitly signal that the confidence data would be re-coded to match the structure of the Type-1 cued conditions for direct comparison.

To allow for a direct comparison between Type-1 and Type-2 decisions, data from the confidence condition were subsequently re-coded to match the structure of the cue conditions.

We believe the revised Methods section now provides the clarity and detail you rightly requested.

394: It's not "much smaller" than pre-expectation

We agree and revised this result part as "*The criterion shift was significantly larger in the confidence condition than each of the four cue conditions. This difference was the smallest for the post-expectation condition with the Bayes Factor indicating lack of strong evidence ($t(251) = 2.15$, $p = .03$, $BF_{10} = 1.59$).* "

Figure 3B: y-axis should start at zero (or have a break if need be)

We have extended the y-axis to zero.

401: This says p=.06 but the graph says it's < .05 (an asterisk). But again, why are you making these comparisons? What's the hypothesis?

We thank the reviewer for pointing out the mismatch and for the importance of clearly indicating why the analysis is reported in the first place. We realized that the asterisk in the figure corresponding to the p=.06 value was a mistake, and it has been removed.

To better organize the pairwise comparison results and reflect their rationales, we split the results into four parts: "Overall pattern", "Type-2 vs. Type-1 judgments", "Pre-cues vs post-cues", and "Expectation vs reward". As we mentioned in response to another comment above, we also added our rationales for these specific types of comparisons and related hypotheses in the Introduction, so that readers now can more easily to follow the logic.

408: Again, it's worth pointing out that others (including Locke et al.) assume that d' is defined by the stimuli and NOT affected by rewards/priors. I presume you made that point in the earlier papers, but it can't hurt to make it again here.

We thank the reviewer for this helpful suggestion. We fully agree that a standard assumption in the field is that priors and rewards don't change the estimated d' value (Green & Swets, 1966; Locke et al., 2020; Macmillan & Creelman, 2004). However, several other empirical papers have examined this issue and found evidence for a decrease in d' in the presence of predictive cues (de Lange et al., 2013; Haddara & Rahnev, 2024; Rahnev, Lau, et al., 2011). We agree it would

be helpful to further emphasize this point in the beginning of the d’ drop results. We edited the relevant text and paste it below:

To note, a standard assumption supported by certain empirical evidence in the field is that biased expectation and reward cues do not change the estimated d’ value (Green & Swets, 1966; Locke et al., 2020; Macmillan & Creelman, 2004). However, there is also empirical evidence demonstrating a decrease in d’ in the presence of predictive cues (de Lange et al., 2013; Haddara & Rahnev, 2024; Rahnev, Lau, et al., 2011). Therefore, we hypothesized that biased expectation and reward cues would induce additional computational noise when placing the decision criterion, and thereby lead to a decrease in d’.

410: the computational noise for the biased criteria manifests as: well, I'd say the noise is equivalent to a drop in d'. By the way why not scatterplot all the shifts vs. d' values to show the correlation?

Thank you for this note on the terminology. We feel that “equivalent” is a bit too strong as it could be seen as implying that there is a direct mapping between d’ drop and computational noise. However, the link between the two is somewhat indirect as it depends also on the size of the criterion shift. Therefore, we have edited the sentence to read: “*Previous research has indicated that computational noise for the biased criteria results in a drop in estimated stimulus sensitivity (d’) compared to the d’ for the unbiased criterion.*”

Thanks for this insightful suggestion about correlating the criterion shifts and d’ drops. We ran the correlation analyses between criterion shift and d’ drop in each condition and generated the corresponding scatterplots. The results demonstrated that criterion shift and d’ drop values are significantly positively correlated in all conditions except for post-expectation condition (where the correlation was still positive but not significant). We now include this result in the main paper and paste the corresponding figure below:

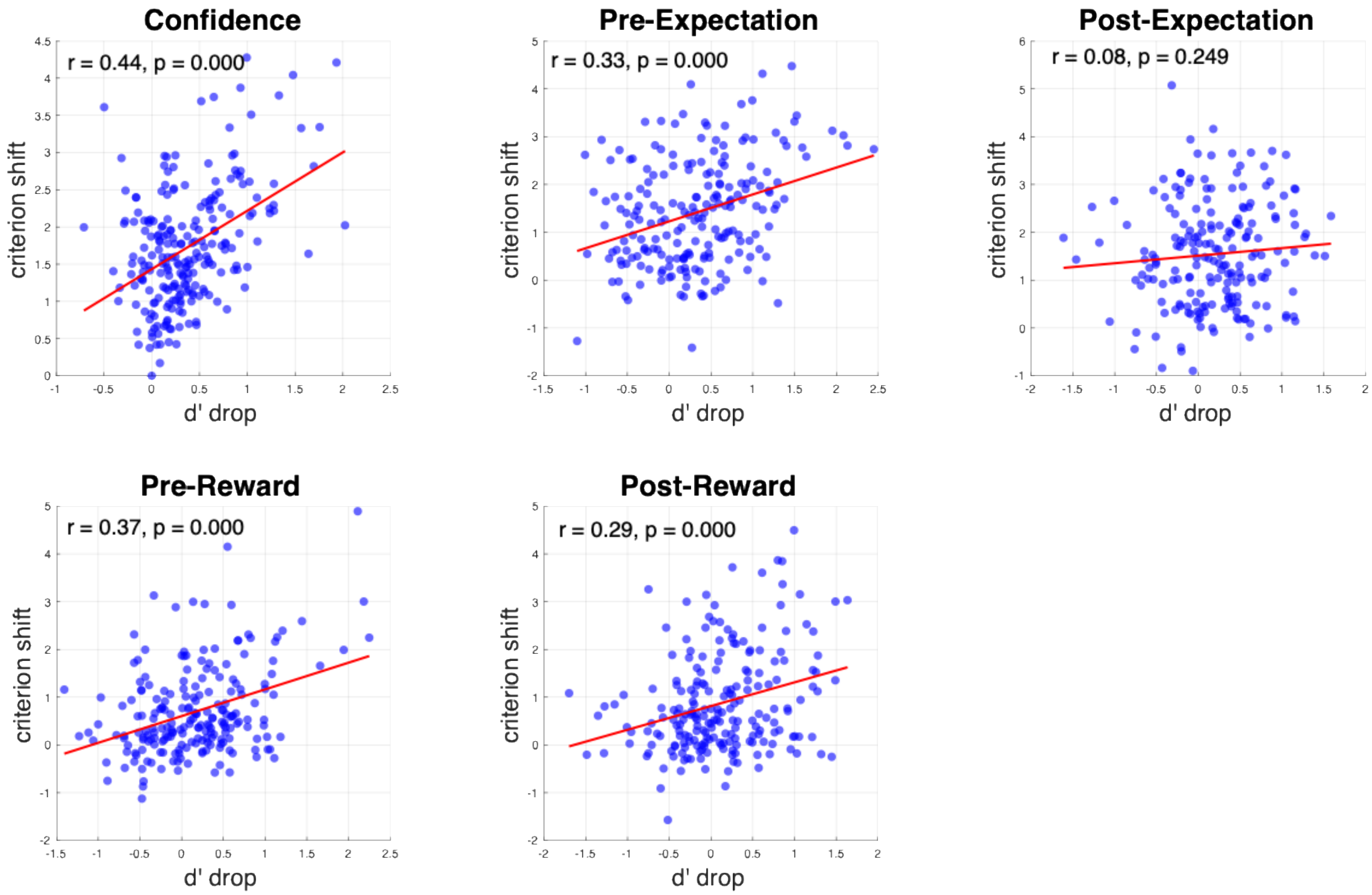

Figure 5. Correlations between criterion shift and d' drop*. Each panel displays the relationship between the degree of criterion shift and d' drop for a specific experimental condition. Each dot represents an individual subject. The red line indicates the linear regression fit, with Pearson's r and p-values inset. As indicated by the r and p-values, significant positive relationships were observed between the degree of criterion shift and d' drop in all conditions except for the post-expectation condition.*

Figure 4A: y-axis should start at zero (or at least show a break)

We extended the y-axis to zero.

441: larger than the one -> larger than in 3 of the 4 cue conditions (or some such rephrase)

Corrected.

439-451: This is an example of a paragraph that feels like a data dump with lots of comparisons but no reference to impact on hypotheses you have nor what the results mean.

We thank the reviewer for point this out. We agree that our original paragraph presented results without sufficient reference to their implications. As in the result section on the criterion shift, we have now revised the section to explicitly connect the findings to our hypotheses.

541: You might find our anti-DDM paper (Sun & Landy, 2016) relevant here. Or not. ;^)

We thank the reviewer for pointing us to this work. We agree that Sun & Landy (2016) is highly relevant, as it provides empirical evidence that perceptual decisions can deviate from the predictions of drift-diffusion models (DDM). We have revised the discussion to incorporate the findings from this paper.

This conclusion mirrors the one by Sun and Landy (2016) who proposed that perceptual decisions may involve a 2-stage "estimate-then-decide" model. Within this framework, both pre- and post-cues may have minimal influence on the estimation stage and primarily affect the decision stage, which would explain why there are no large behavioral differences between pre- and post-cues.

Reviewer #2

This is a very interesting article aiming to assess whether Type 1 and Type 2 decisions feature similar levels of computational noise once the complexity of the tasks is equated. I enjoyed reading the paper, particularly the clever design that manipulates complexity through confidence, cue, and reward conditions. The study addresses an important theoretical debate and is carefully executed.

We thank the reviewer for their positive feedback and insightful comments. We are pleased they found the article interesting and the design clever. Below, we address each of the points raised.

I have the following comments and questions:

1) On the notion of complexity:
- Complexity is at the core of the paper's argument, but it is not clearly defined. How can the complexity of a task be objectively assessed?

We agree that our initial use of "complexity" was not sufficiently defined. We have now removed the term complex/complexity throughout the manuscript and simply explain the issue as one of having to judge the strength of sensory evidence (in confidence judgments, as well as in the presence of priors or rewards) vs. having to make a simple categorization about which of two categories is associated with higher sensory evidence. We paste the updated Abstract below as an example of making such changes:

Mounting evidence supports the existence of Type-2, metacognitive noise that affects human confidence judgments. The existence of this noise has led to the hypothesis that metacognitive judgments arise from a metacognitive system that is separate from the decision-making system responsible for Type-1 decisions. However, Type-2 decisions are different than standard Type-1 decisions in that they require evaluating the strength of sensory evidence rather than just a

categorical judgment. Here, we investigated whether Type-2 computational noise still exceeds Type-1 computational noise when both judgments require evaluation of the strength of sensory evidence. Subjects (N=319) performed a simple perceptual discrimination task and in different conditions provided Type-2 confidence judgments or Type-1 judgments based on biased expectations or unequal rewards. All these judgments require a similar fine-grained evaluation of the strength of sensory evidence (e.g., is the evidence high enough to justify a high-confidence rating or choosing the alternative with smaller reward). We first confirmed that the confidence and expectation conditions resulted in similarly biased criteria. Critically, both simple signal detection analyses and formal computational modeling confirmed that the confidence and expectation conditions exhibited comparable levels of computational noise. In contrast, the reward conditions exhibited both less biased criteria and smaller computational noise. These findings demonstrate that Type-1 and Type-2 decisions exhibit computational noise of similar magnitude. More generally, our results have implications for the debate on whether Type-1 and Type-2 decisions are made by separate systems.

- Is complexity the only relevant construct? Alternative mechanisms (e.g., attention modulation by cues or rewards) could also explain the results.

We acknowledge that we cannot determine the exact sources of the computational noise, and an attention mechanism could be a factor that contributes to the noise. However, it should be noted that the attention might play a role in the Type-1 decision with expectation and reward cues, but it is unclear how it could relate to Type-2 confidence. We now use attention as a possible difference in the mechanisms underlying the effects for Type-1 vs. Type-2 judgments. We pasted the relevant text from the Discussion below:

However, on the other hand, the fact that two processes feature computational noise of similar magnitude does not necessitate the existence of common mechanisms. For example, inattention to the cue information might induce noise in the criterion placement in the cue conditions, while some other mechanisms might contribute to the noise in the confidence condition. Future research should estimate the magnitude of computational noise for Type-1 and Type-2 decisions under different experimental paradigms to more convincingly establish whether additional mechanisms contribute to noise in Type-1 vs. Type-2 judgements.

- Why do reward manipulations not produce the same pattern as expectation cues, if the complexity is assumed to be equivalent across treatments?

We thank the reviewer for pointing out this issue. The pattern for criterion shift (i.e., reward cues lead to smaller criterion shift than expectation cues) is actually well-noted in the existing literature. For the estimated computational noise difference between the two cue types, we think three issues should be noted here:

First, the BF values for the difference and similarity in the noise magnitude between expectation and reward conditions were very small. Therefore, it is hard to use the current data to draw a clear response to the question.

Second, it might be possible that the smaller criterion shift in the reward conditions leads to smaller d' drop and our model doesn't sufficiently account for this when estimating computational noise.

Third, there could be true computational/psychological differences between expectation and reward cues, but the current study doesn't allow us to speculate what those may be.

We now reorganized the results, and at the end of each "Expectation vs reward" section, we added an explanation to the observed pattern.

Criterion shift
This result replicates many previous studies showing that subjects tend to shift their criteria less when given reward compared to expectation cues (e.g., Locke et al., 2020; Rahnev & Denison, 2018).

d' drop for biased criteria
This pattern resembles the criterion shift patterns (Figure 3B), suggesting that the differences in d' drop between expectation and reward cues might be driven by the differences in criterion shift.

Magnitude of computational noise
These results may be due to computational or psychological differences between expectation and reward cues, or, alternatively, it may be that the lognormal meta noise model does not sufficiently correct for the large difference in criterion shift between the conditions when estimating the computational noise. However, given the inconclusive Bayes factors, it is premature to postulate mechanisms that would predict either clear differences or a clear lack of differences between the expectation and reward cues.

2) Exclusion criteria could be better explained:
- A large number of exclusion criteria are applied. It would be helpful to know precisely how many participants were excluded under each criterion.

We thank the reviewer for this suggestion. In the revised manuscript, we have added a detailed breakdown listing the precise number of subjects excluded based on each criterion:

After applying the exclusion criteria, the final number of subjects for each condition was: confidence (N=288), pre-expectation (N=301), post-expectation (N=296), pre-reward (N=273), and post-reward (N=257).

- Some participants are excluded from certain conditions but remain in others. Are the results robust if participants are excluded entirely once they fail in any one condition?

Yes, we applied the suggested exclusion and found the overall conclusion remain the same. We

demonstrate that in Figure S2, and also paste the figure below:

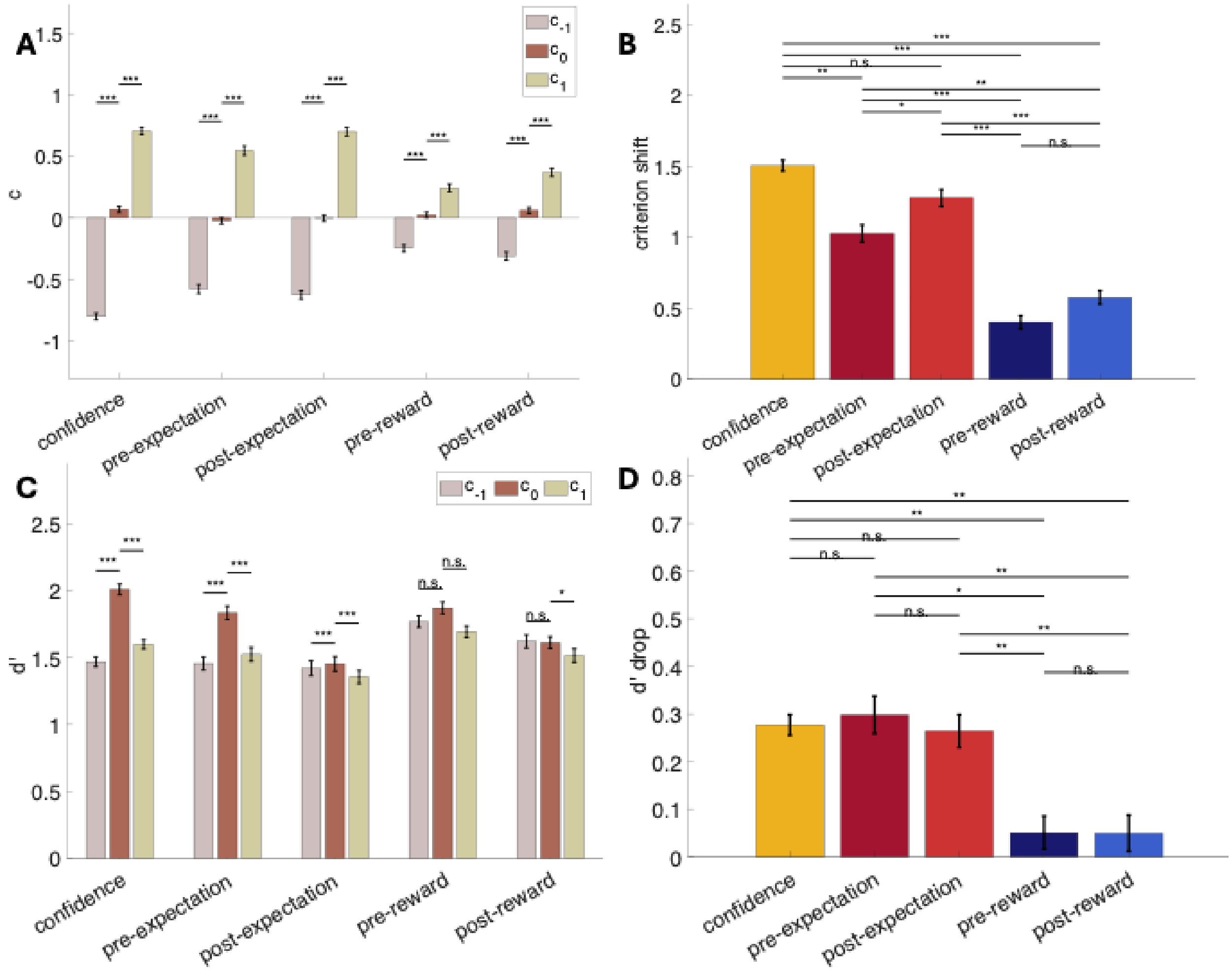

Figure S2. Replication with the subset of subjects who did not fail any exclusion criteria in any condition. *In this analysis, we used stricter exclusion criteria where a subject’s data was excluded from all conditions as long as it had to be excluded from even a single condition, resulting in 223 subjects who were taken into the main analysis. The number of subjects having valid estimated d’ drop and criterion shift was 127, 111, 112, 123, 133 for confidence, pre-expectation, post-expectation, pre-reward, and post-reward conditions. The results were again similar to those in the main manuscript (Figures 3 and 4). A) Criterion location in each condition for the three criterion locations (c_{-1}, c_0, c_1). This pattern is identical to the main analyses (Figure 3A). B) The degree of criterion shift for each condition. The pattern roughly replicated the one in main analyses. However, there was no significant difference between confidence condition and post-expectation condition (p = 0.106), while the difference between pre-expectation and post-expectation conditions was significant (p = 0.049). C) d’ values associated with three criterion locations (c_{-1}, c_0, c_1) in each condition. The pattern replicated the one in main analyses, except that the d’ for biased criteria was not significantly lower than the one for unbiased criteria in the pre-reward condition (both p’s > 0.829). D) The degree of d’ drop for each condition. The pattern was similar to that observed in the main analyses (Figure 4B), such that the d’ drop in the*

*confidence and expectation conditions was greater than that in the reward conditions. However, there was no significant difference between the confidence and post-expectation conditions ($p = 0.408$), nor between the pre-expectation and post-expectation conditions ($p = 0.163$). In addition, we observed a significant difference between the post-expectation and two reward conditions (p's < 0.007). Overall, these results were in line with the main manuscript's findings. Error bars show SEM. ***, $p < .001$; **, $p < .01$; *, $p < .05$; n.s., not significant.*

3) Some design choices should be explained / justified :
- Why was a 500 ms blank screen included in the confidence and post-cue conditions, while pre-cue conditions involved a 1500 ms cue?

A 1500-ms cue was presented in pre-cue conditions to allow subjects to process the information from the pre-cues. In pilot experiments, we tried having 500-ms blank screens before the stimulus, but we found that this was not enough for subjects to fully process the cues. We now justify this design choice in the Methods, and paste the relevant text below:

The cue presentation time is longer than the blank screen prior to the stimulus in the other conditions as this allows subjects to better process the cue information.

- Why were there 100 confidence trials (2 blocks) but 150 cue/reward trials (3 blocks)?

The confidence condition included 100 trials in total. This is because the confidence condition is more efficient with all 100 trials contributing to the estimation of all 3 criteria (the decision and the two confidence criteria). In contrast, each of the three cue types (left-biased, right-biased, and unbiased) in the cue conditions only contributed to the estimation of a single criterion. Thus, while the confidence condition had fewer total number of trials, it actually provided higher precision in the estimation of the criteria. We now clarify this in the Methods:

The confidence condition then consisted of two blocks of 50 trials, with a total of 100 trials. The trial number in the confidence condition was lower than each of the four cue conditions. This is because in the cue conditions, each type of cue only contributed to the estimation of a single criterion (the unbiased decision criterion and the two biased criteria), while 100 trials in the confidence condition contributed to the estimation of all 3 criteria.

- In post-cue conditions, why not require participants to view the cue/reward screen for 1500 ms after the stimulus presentation, matching the timing of pre-cue conditions?

In post-cue conditions, the cue was presented simultaneously with the response prompt to create a clean distinction where information was available either entirely before the stimulus or only at the moment of the decision. We developed this design from piloting results to ensure most subjects can process the expectation and reward information properly and maintain above chance-level performance while avoiding celling effect. We felt that forcing a longer break for some conditions may lead to working memory demands that differ across conditions. We now justify our design decision in the Methods:

We presented cues with response prompt simultaneously because presenting separate post-cue and decision screens may lead to the post-cue not getting properly processed (if the post-cue duration is too short) or unnecessarily slow down the decision (if the post-cue duration is too long).

4) It is not entirely clear how choices are reused to compute Type 1 SDT variables in the confidence condition. Are the same choices counted multiple times (e.g., once for “right,” once for “left,” and again for unbiased)? Clarification would help.

We thank the reviewer for this clarification question. Trials in confidence condition were used three times to calculate the right-biased, left-biased and unbiased criteria. We now made this recoding detail more explicit:

To allow for a direct comparison between Type-1 and Type-2 decisions, data from the confidence condition were subsequently re-coded to match the structure of the cue conditions. Specifically, in the confidence reports, the confidence criterion separating low and high confidence for right responses acts similarly to a biased decision criterion obtained when using a left-biased instruction cue (Figure 1B). Indeed, in both cases subjects report right (for the left-biased instruction cue) or right with high confidence (for confidence reports) only when there is very strong evidence for a right choice. Therefore, to match the left-biased instruction cues, “right” choices with high confidence were then treated as “right” choices, while all other responses were treated as “left” choices. Similarly, to match the right-biased instruction cues, “left” choices with high confidence were treated as “left” choices, while all other responses were treated as “right” choices (Figure 1B,C). Finally, to match the unbiased instruction cues, we retained the original choices disregarding the confidence ratings (Figure 1A,B). All trials in confidence condition were used for each recoding process.

5) NaN values details are missing :
- How many subjects had NaN values for c and d’?
- How many required the modified formulas using only left or right data?

The number of subjects taken into data analysis for each condition is: confidence (N=288), pre-expectation (N=301), post-expectation (N=296), pre-reward (N=273), and post-reward (N=257).

Among those subjects, the number of subjects that had at least one NaN value for c and d’ in each condition was: confidence (N=129), pre-expectation (N=162), post-expectation (N=153), pre-reward (N=120), and post-reward (N=98). Nevertheless, we did not exclude those subjects unless they had the NaN values for mid c and d’ or two NaN values for the left and right c and d’.

Subjects with only one missing value for a biased-cue trial who required the use of modified formulas, the counts were: confidence (N=70), pre-expectation (N=68), post-expectation (N=54), pre-reward (N=57), and post-reward (N=52). Therefore, in the analysis of criterion shift

and d’ drop, the number of subjects taken into the analysis in each condition was: confidence (N=229), pre-expectation (N=207), post-expectation (N=197), pre-reward (N=210), and post-reward (N=211).

We clarified the number of subjects taken into the criterion shift and d’ drop analyses in the manuscript and paste here:

We used the modified formulas for 70, 68, 54, 57, and 52 subjects in the confidence, pre-expectation, post-expectation, pre-reward, and post-reward conditions, respectively (note that if $c = NaN$, then $d' = NaN$, and vice versa, so the number of modified formulas is the same for c_{shift} and d'_{drop}). The final sample sizes for the analyses of criterion shift and d’ drop were 229, 207, 197, 210, and 211 subjects in the confidence, pre-expectation, post-expectation, pre-reward, and post-reward conditions, respectively.

We also applied the edge correction to estimate c and d’, resulting in no use of the modified formulas to compute criterion shift and d’ drop. We found similar results pattern without the modified formulas too. We paste the relevant text and figure below:

Notably, we found qualitatively similar results when applying the Hautus’s edge correction and not using the modified formulas to compute criterion shift and d’ drop (Figure S4).

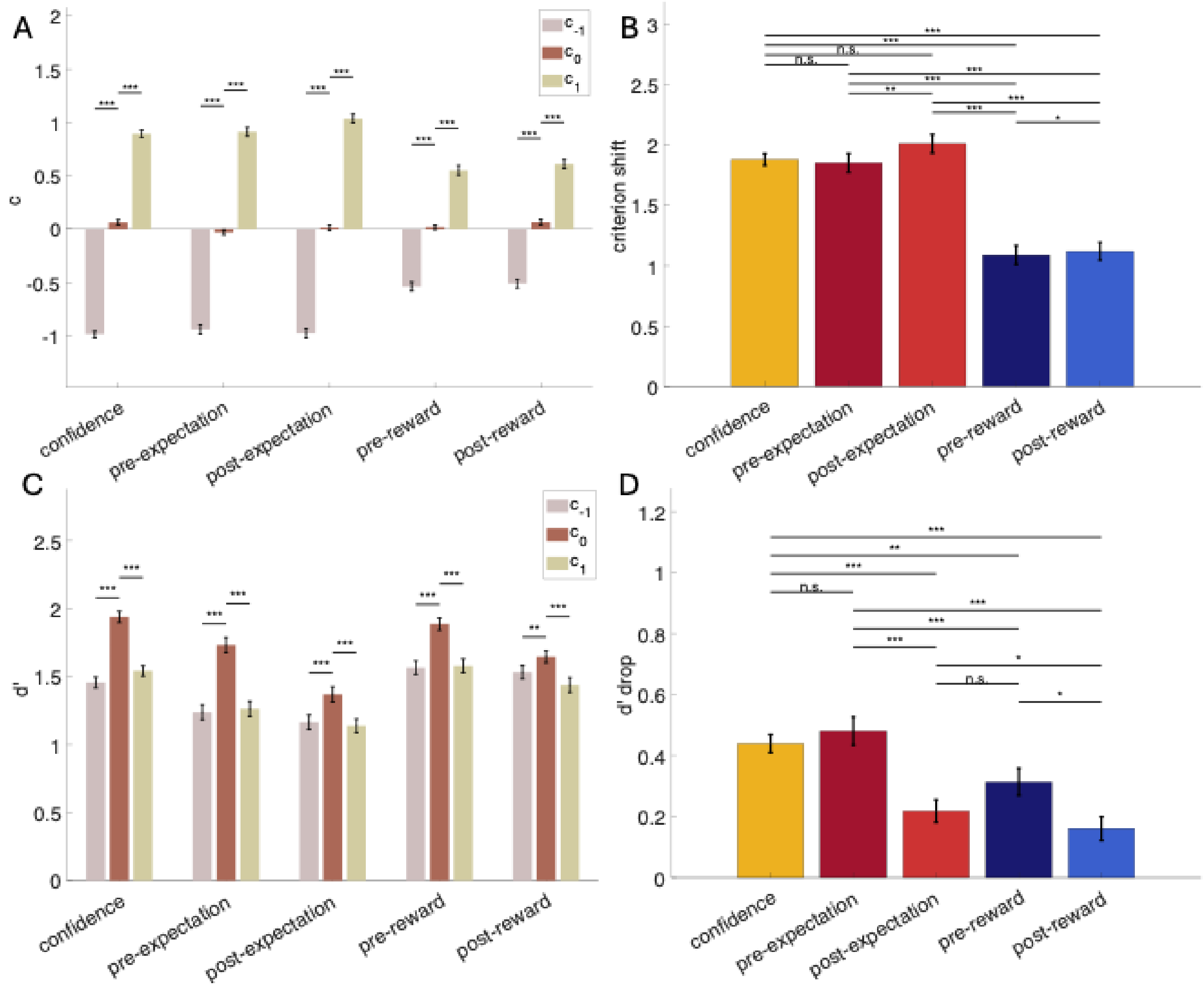

Figure S4. Results with applying edge correction. *Here, we followed standard Signal Detection Theory (SDT) analysis and applied padding to zero response counts to avoid zero hit rates and false alarm rates when estimating c and d' values. The results were similar to those in the main manuscript (Figures 3 and 4). A) Criterion location in each condition for the three criterion locations (c_{-1}, c_0, c_1). This pattern is identical to the main analyses (Figure 3A). B) The degree of criterion shift for each condition. The pattern roughly replicated the one in main analyses, such that reward conditions had smaller criterion shift. However, there is no significant difference between confidence condition and two expectation conditions (p = 0.32 and 0.45 for pre-expectation and post-expectation conditions, respectively). Furthermore, post-cue conditions had significantly greater criterion shift than pre-cue conditions (pre-expectation vs. post-expectation, p = 0.005; pre-reward vs post-reward, p = 0.033). C) d' values associated with three criterion locations (c_{-1}, c_0, c_1) in each condition. The pattern is identical to the main analyses (Figure 4A), except that the d' for c_0 was significantly higher than c_{-1} (p = 0.007). D) The degree of d' drop for each condition. The pattern is identical to the main analyses (Figure 4B) so that confidence and pre-expectation conditions had similar degree of d' drop and they were higher than the ones in other conditions. However, the d' drop in the post-expectation condition was significantly greater than in the post-reward condition (p = 0.031), and the d' drop in the pre-*

*reward condition was also significantly greater than in the post-reward condition (p = .030). Overall, these results were in line with the main manuscript's findings. Error bars show SEM. ***, p < .001; **, p < .01; *, p < .05; n.s., not significant.*

- Are results robust if these cases are excluded outright rather than adjusted?

This subset of subjects basically corresponded to those we included in the model fitting process, though in this analysis we further required a subject to have a positive d' across all three cue types in a condition. The sample size for the model fitting procedure was 155, 126, 123, 151, and 155 for confidence, pre-expectation, post-expectation, pre-reward, and post-reward conditions respectively. We ran the same analysis on those subjects, and found qualitatively similar results. We now include those results in the Figure S7, and paste it below:

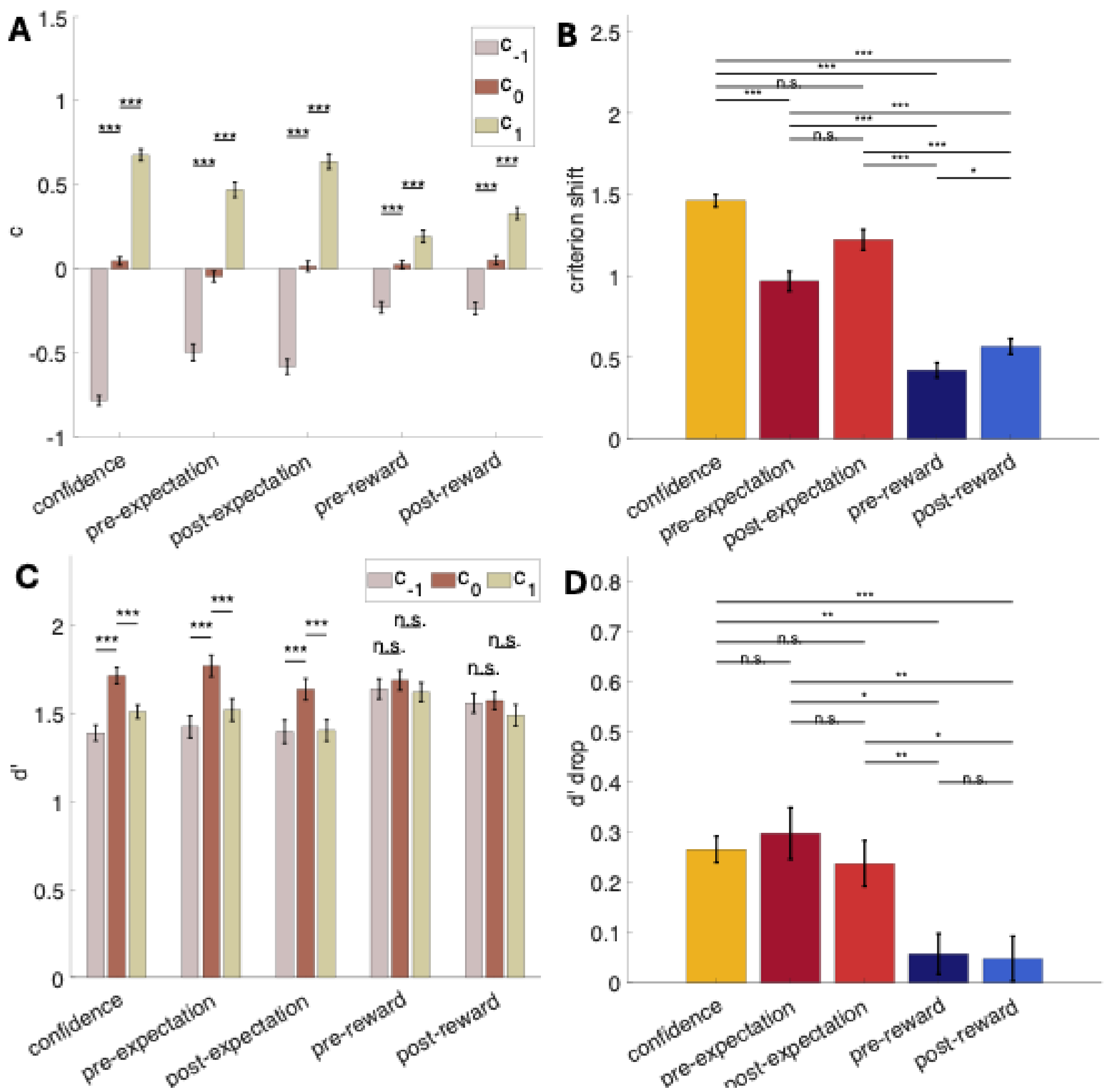

Figure S7. Replication with the subset of subjects used in model fitting. *In the model fitting procedure, we included a subset of 155, 126, 123, 151, and 155 subjects for confidence, pre-expectation, post-expectation, pre-reward, and post-reward conditions. Notably, these subjects were those whose data did not need the modified formulas when computing criterion shift and*

*d’ drop. Here we examined whether we could replicate the main findings on those subjects. A) Criterion location in each condition for the three criterion locations (c_{-1}, c_0, c_1). This pattern is identical to the main analyses (Figure 3A). B) The degree of criterion shift for each condition. The pattern roughly replicated the one in main analyses. However, there is no significant difference between confidence condition and post-expectation conditions (p = 0.125), while the difference between pre-reward and post-reward conditions was significant (p = 0.04). C) d’ values associated with three criterion locations (c_{-1}, c_0, c_1) in each condition. The pattern replicated the one in main analyses, except that the d’ for biased criteria was not significantly lower than the one for unbiased criteria in both reward conditions (all p’s > 0.128). D) The degree of d’ drop for each condition. The pattern was identical to that observed in the main analyses (Figure 4B), such that the d’ drop in the confidence and expectation conditions was greater than that in the reward conditions. However, there was no significant difference between the confidence and post-expectation conditions (p = 0.96), nor between the pre-expectation and post-expectation conditions (p = 0.167). In addition, we observed a significant difference between the post-expectation and post-reward conditions (p = 0.012). Overall, these results were in line with the main manuscript's findings. Error bars show SEM. ***, p < .001; **, p < .01; *, p < .05; n.s., not significant.*

6) Were any corrections applied for multiple comparisons in the pairwise contrasts between conditions? If not, this should be acknowledged.

We thank the reviewer for this important question. We did not apply corrections for multiple comparisons in our pairwise contrasts. This decision was based on the fact that our analyses were designed to test a limited set of specific, a priori hypotheses. To ensure this is clear to the reader, we have now added a sentence acknowledging this approach in the Statistical analysis section:

We did not apply corrections for multiple comparisons to the planned pairwise contrasts. This approach was chosen because our study was designed to test specific, pre-defined hypotheses guided by the existing literature rather than to conduct a broad set of exploratory analyses.

7) The criterion shift is consistently higher in the confidence condition compared to all others. I did not see an explicit explanation for this in the text.

We thank the reviewer for pointing out this finding. To our knowledge, this is the first study reporting such differences. Although we do not have a certain explanation to this phenomenon, we do have some speculative thoughts. We added a paragraph in Discussion regarding this issue, and paste it below:

One surprising finding was that people generally shifted their criteria more in the confidence than in the expectation and reward conditions. This is despite the fact that the optimal criterion shift was identical across all conditions. Note, however, that the difference between the confidence and expectation conditions was much smaller (though significant) than the difference between the confidence and the reward conditions. As we reviewed above, the

smaller criterion shift in the reward condition is likely due to subjects prioritizing accuracy over the pure gain-maximizing strategy. It is possible that a similar, but smaller, effect leads to the difference between confidence and expectation: namely, subjects may be prioritizing the sensory information over the probabilistic information from the expectation cue, whereas the confidence condition removes the need to prioritize one source of information over another. Nevertheless, the possibility is currently speculative and would need to be directly tested in future research.

8) The results sometimes show differences between pre- and post-cue manipulations (e.g., d' drop for expectation). However, in the discussion, these manipulations are presented as similar. The discrepancy should be addressed more explicitly.

We thank the reviewer for this suggestion. We made a couple of changes to address this issue. First, we now revised the Results section so that the comparison between pre- and post-cues always appeared in dedicated paragraphs, making it easier to present these results in more detail. Second, we included a more robust discussion in the Discussion section that discusses both similarities and differences in more detail, acknowledging that there are some differences found. We paste the relevant paragraph in the Discussion section below:

Additionally, our study revealed largely similar patterns in Type-1 decisions when expectation or reward cues were presented before versus after the sensory stimuli. Bang and Rahnev (2017) previously reported a larger criterion shift when expectation cues appeared after, compared to before, the sensory stimuli. In our study, we observed the same directional pattern. However, the differences were not statistically significant, though the Bayes factors indicated lack of clear evidence for either the null or the alternative hypotheses. More importantly, there was no difference in the estimated computational noise between the pre- and post-cue conditions (for either expectation or reward), though examining d' drop did reveal a tendency for the pre-cue conditions to show larger d' drop than post-cue conditions. Overall, these results suggest that pre- and post-cues are not equivalent, but that the differences between them are subtle.

9) It would be useful to clarify what the computational model adds beyond the observed d' drop. Much of the paper's central claim relies on model-based estimates of noise, whereas the raw d' drop results appear less clear.

We thank the reviewer for pointing out this crucial issue. We've shown empirically that metacognitive noise increases for criteria set further from the decision criterion. In addition, we conducted new correlation analyses and found that the degree of criterion shift was significantly correlated with the degree of d' drop (see the figure below). Therefore, d' drop is expected to be larger for criteria further from the decision criterion.

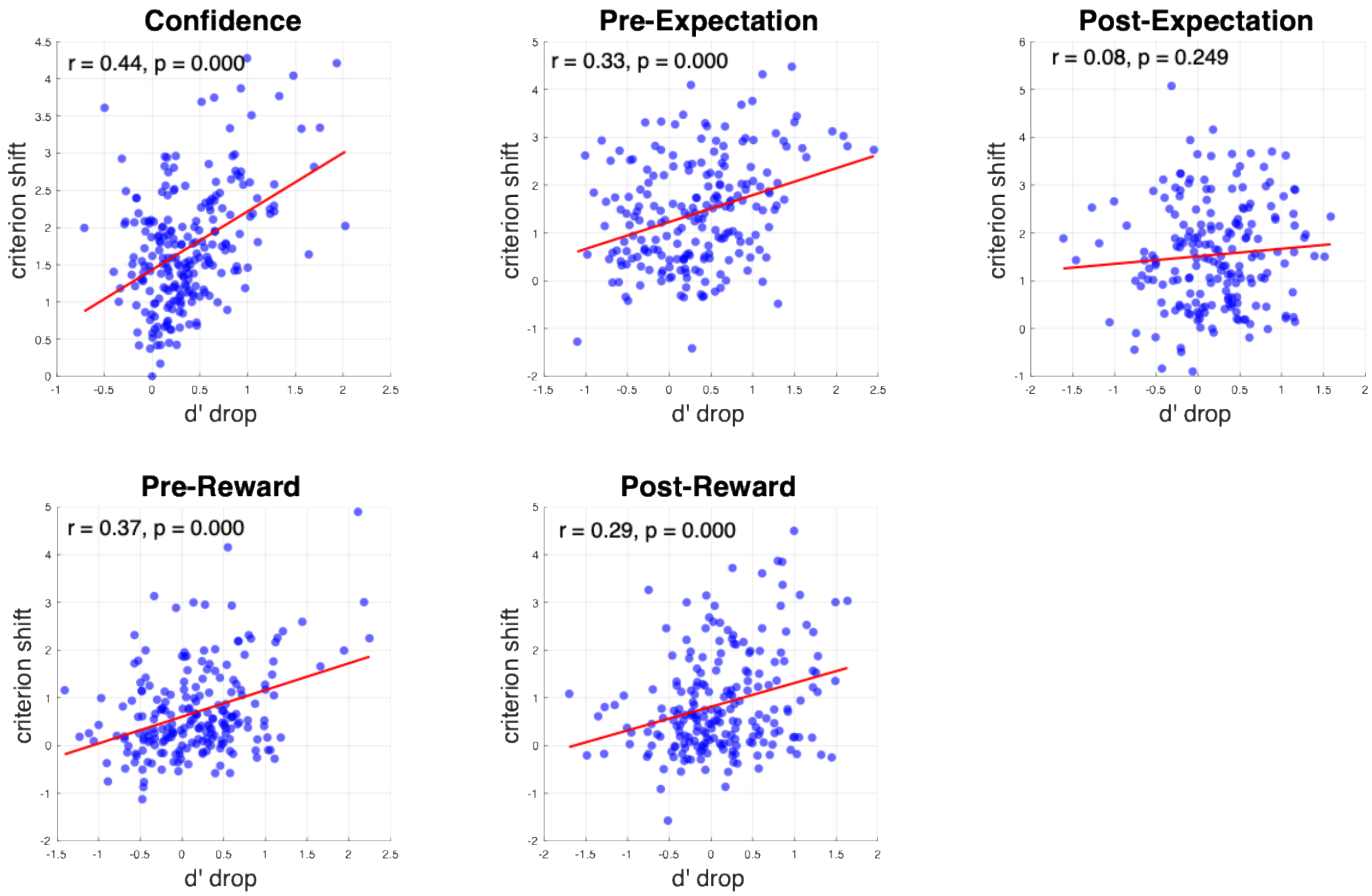

***Figure 5. Correlations between criterion shift and d' drop**. Each panel displays the relationship between the degree of criterion shift and d' drop for a specific experimental condition. Each dot represents an individual subject. The red line indicates the linear regression fit, with Pearson's r and p-values inset. As indicated by the r and p-values, significant positive relationships were observed between the degree of criterion shift and d' drop in all conditions except for the post-expectation condition.*

This finding suggests that d' drop would be affected by the degree of criterion shift, and therefore represents an imperfect measure of computational noise. Our computational model takes into account the size of the criterion shift when computing the noise. We further clarified the rationale of computational modeling approach in the manuscript and paste it below:

These empirical results and model considerations demonstrate that d' drop is affected by the degree of criterion shift, leading to the conclusion that d' drop is an imperfect measure of computational noise. To overcome this issue, we directly fitted our data with the lognormal meta noise model to estimate the magnitude of computational noise in criterion placement in each of the five conditions.

10) The discussion of DDM implications may be too strong. Given that pre/post cue manipulations sometimes differ depending on the index used, the conclusions should be stated more cautiously.

We agree that we worded the DDM implications using overly strong language. We have now edited the paragraph to discuss the implications more carefully. We paste the revised version of the paragraph below:

The similarity between pre- and post-cues has implications about how evidence accumulation models, such as the drift diffusion model (DDM), conceptualize the influences of cues in perceptual decision making. The DDM posits that perceptual decision making is a process of accumulation terminated when the evidence reaches the same stereotyped boundary (Gold & Shadlen, 2007; Ratcliff & McKoon, 2008; Ratcliff & Smith, 2004; Voss et al., 2008). Within this framework, pre-cues are typically modeled as a change in the starting point or drift rate bias of the accumulation (Dekel & Sagi, 2020; Sánchez-Fuenzalida et al., 2023; Starns & Ratcliff, 2012; Summerfield & de Lange, 2014), which means that noise in the pre-cue conditions should be modeled as noise in the starting point or drift rate bias adjustment. However, noise in the post-cue conditions cannot be modeled in the same way since post-cues are presented too late to meaningfully influence either the starting point or drift rate of the accumulation. Instead, noise in the post-cue conditions may be modeled as noise in decision threshold. Since these are completely separate mechanisms, one may expect larger differences between pre- and post-cues in both criterion shift and computational noise than what was found in our data. Our results suggest that standard pre-cues may act not only by biasing starting point or drift rate, but also by biasing late decisional processes. This conclusion mirrors the one by Sun and Landy (2016) who proposed that perceptual decisions may involve a 2-stage "estimate-then-decide" model. Within this framework, both pre- and post-cues may have minimal influence on the estimation stage and primarily affect the decision stage, which would explain why there are no large behavioral differences between pre- and post-cues. Our results thus point for a need of further research on the topic of how pre-cues affects decision making within evidence accumulation models.

11) The discussion on one vs. two systems and on potential common mechanisms could be further developed. Beyond the current findings, what empirical tests could distinguish these views? The section on DDM implications provides such concrete predictions, but the discussion of one vs. two systems is more general and could benefit from the same level of specificity.

We agree with the reviewer that it would be beneficial to add more specific hypothesis of how to further distinguish the one vs. two-system view. However, unlike for the DDM section, it's hard to come up with clear empirical tests that will neatly distinguish between the two possibilities (one vs. two systems). Nevertheless, an important step would be to identify in a more precise fashion the sources of noise for Type-2 vs. Type-1 judgments. We revised the corresponding paragraph in the Discussion section to address this issue, and paste it below:

Our study also has implications for the debate about whether Type-1 and Type-2 decisions are made by the same system or different systems. The similar magnitude of the computational noise between Type-1 perceptual and Type-2 metacognitive decisions might challenge the dominant view that Type-1 and Type-2 judgments are operated by two distinct (though interrelated) systems (Fleming & Daw, 2017; Mamassian, 2020; Nelson & Narens, 1990). This

two-system perspective has been largely supported by the existence of confidence-accuracy dissociations, where various manipulations have been shown to affect Type-2 confidence levels without affecting Type-1 performance (De Gardelle & Mamassian, 2015; Fleming et al., 2015; Rahnev, Bahdo, et al., 2012; Rahnev, Maniscalco, et al., 2011, 2012; Samaha et al., 2017; Zylberberg et al., 2012). Nevertheless, such confidence-accuracy dissociations can often be modeled without assuming distinct systems (Fetsch et al., 2014; Peters et al., 2017; Rahnev, Maniscalco, et al., 2011, 2012; Shekhar & Rahnev, 2024b; Zylberberg et al., 2012). Conversely, a few recent studies suggest that Type-1 and Type-2 judgments may be produced by a unified system. For instance, Gao et al. (2025) found that the way by which people use the uncertainty of sensory evidence to integrate multisensory signals into decisions is identical to how they use such uncertainty in confidence judgments. Moreover, Zheng et al. (2023) provided evidence that a unified system might generate both metacognitive and meta-metacognitive judgments, a conclusion also consistent with the findings of Recht et al. (2022). By demonstrating the existence of similar levels of computational noise in Type-1 and Type-2 decisions, the current study further questions the necessity of postulating the presence of two separate systems for Type-1 and Type-2 judgments. To further test whether Type-1 and Type-2 judgments are produced by the same system, future research should examine not just the magnitude of the noise but also identify and compare the precise sources of noise.

Reviewer #3

I co-reviewed this manuscript with one of the reviewers who provided the listed reports. This is part of the Communications Psychology initiative to facilitate training in peer review and to provide appropriate recognition for Early Career Researchers who co-review manuscripts.

We thank both reviewers for their joint and constructive feedback, which has been very helpful in improving the manuscript.

Response to Reviewers

Reviewer #1 (Remarks to the Author):

Re-review of "Type-1 and Type-2 decision feature computational noise of similar magnitude"
Reviewer: Michael Landy

This version is vastly clearer and more straightforward than the previous. I think my comments are few and all typos (page/para/line):

9/3/8: except exclusion criterion FOUR

Figure 2 legend: cues->cue (twice)

12/2/8: that -> than

13/1/3: conditions -> condition

21: first display equation has an extra ")" in the denominator

37/2/1: proportionate -> proportional

We thank the reviewer for the positive feedback and for another careful reading of our manuscript. We have corrected all of these grammatical and typographical errors in the revised version.

Reviewer #2 (Remarks to the Author):

The authors have addressed all of my queries, and I'm pleased with the revised manuscript.

We thank the reviewer for the positive feedback and are glad that our revisions have addressed all of their concerns.

Reviewer #3 (Remarks to the Author):

I co-reviewed this manuscript with one of the reviewers who provided the listed reports. This is part of the Communications Psychology initiative to facilitate training in peer review and to provide appropriate recognition for Early Career Researchers who co-review manuscripts.

We thank both reviewers again for their joint effort and constructive feedback throughout the review process. Their comments have been very helpful in improving the manuscript.
