# Supplementary material for: Type-1 and type-2 decisions feature computational noise of similar magnitude
Source: Commun Psychol. 2026 Apr 16;4:113. doi: 10.1038/s44271-026-00454-3 (PMC13462944; doi:10.1038/s44271-026-00454-3)
Supplement: Supplementary file 2 [file 44271_2026_454_MOESM2_ESM.docx]

**Supplementary Materials**

**Type-1 and Type-2 decisions feature computational noise of similar magnitude**

Yunxuan Zheng, Kai Xue, Medha Shekhar & Dobromir Rahnev

**Figure S1. Replication with preregistered data exclusion criteria.** Utilizing the preregistered data exclusion criteria detailed in the Methods section, we analyzed 271 subjects who had complete data across all five conditions. The results were similar to those in the main manuscript (Figures 3 and 4). A) Criterion location in each condition for the three criterion locations ($c_{-1},c_{0},c_{1}$). The pattern is identical to the main analyses (Figure 3A) B) The degree of criterion shift for each condition. The pattern is the same as in the main analyses (Figure 3B) except that there is no significant difference between the confidence and post-expectation conditions (*p* = 0.11). C) $d’$ values associated with three criterion locations ($c_{-1},c_{0},c_{1}$) in each condition. The pattern is identical to the main analyses (Figure 4A). D) The degree of $d’$ drop for each condition. The pattern is also identical to the main analyses (Figure 4B) except that the difference between post-expectation and post-reward conditions became significant (*p* = 0.004). Overall, these results were in line with the main manuscript's findings. Box plots show 25^th^, 50^th^, and 75^th^ percentiles (box lines), as well as minimum and maximum excluding outliers (whiskers). Individual dots show outliers. Diamonds show means.*****, *p* < .001; ***, p* < .01; **, p* < .05; *n.s.*, not significant.

**Figure S2. Replication with the subset of subjects who did not fail any exclusion criteria in any condition.** In this analysis, we used stricter exclusion criteria where a subject’s data was excluded from all conditions as long as it had to be excluded from even a single condition, resulting in 223 subjects who were taken into the main analysis. The number of subjects having valid estimated d’ drop and criterion shift was 127, 111, 112, 123, 133 for confidence, pre-expectation, post-expectation, pre-reward, and post-reward conditions. The results were again similar to those in the main manuscript (Figures 3 and 4). A) Criterion location in each condition for the three criterion locations ($c_{-1},c_{0},c_{1}$). This pattern is identical to the main analyses (Figure 3A). B) The degree of criterion shift for each condition. The pattern roughly replicated the one in main analyses. However, there was no significant difference between confidence condition and post-expectation condition (*p* = 0.106), while the difference between pre-expectation and post-expectation conditions was significant (*p* = 0.049). C) $d’$ values associated with three criterion locations ($c_{-1},c_{0},c_{1}$) in each condition. The pattern replicated the one in main analyses, except that the $d’$ for biased criteria was not significantly lower than the one for unbiased criteria in the pre-reward condition (both *p*’s > 0.829). D) The degree of $d’$ drop for each condition. The pattern was similar to that observed in the main analyses (Figure 4B), such that the d′ drop in the confidence and expectation conditions was greater than that in the reward conditions. However, there was no significant difference between the confidence and post-expectation conditions (*p* = 0.408), nor between the pre-expectation and post-expectation conditions (*p* = 0.163). In addition, we observed a significant difference between the post-expectation and two reward conditions (*p*’s < 0.007). Overall, these results were in line with the main manuscript's findings. Box plots show 25^th^, 50^th^, and 75^th^ percentiles (box lines), as well as minimum and maximum excluding outliers (whiskers). Individual dots show outliers. Diamonds show means.*****, *p* < .001; ***, p* < .01; **, p* < .05; *n.s.*, not significant.

**Figure S3. Criterion placement stays the same between first and second half in the expectation conditions**. The instructions regarding the expectation cues stated that one choice was three times more likely to be correct (i.e., implied cue validity was 75%). However, in reality, the true cue validity was less extreme (66%), which allowed for more trials with invalid cues. It might be possible that subjects learned the underlying probabilistic structure over time and subsequently reduced their reliance on the expectation cues. To test this hypothesis, we conducted paired t-tests comparing the decision criterion for left-cued and right-cued trials between the first and second halves of each expectation condition. A) We found no significant changes in either $c_{-1}$ or $c_{1}$ across both the pre-expectation and post-expectation conditions (all p’s > .18, all BF₀₁ > 5.91). B) Further, the criterion shift value between first and second half of trials were also similar in the pre-expectation and post-expectation conditions (p’s > .11, BF₀₁ > 4.15). Taken together, these results indicate strong evidence against the notion that subjects learned the true cue validity and thereby shifted their decision criterion more conservatively.

**Figure S4. Results with applying edge correction**. Here, we followed standard Signal Detection Theory (SDT) analysis and applied padding to zero response counts to avoid zero hit rates and false alarm rates when estimating $c$and $d’$values. The results were similar to those in the main manuscript (Figures 3 and 4). A) Criterion location in each condition for the three criterion locations ($c_{-1},c_{0},c_{1}$). This pattern is identical to the main analyses (Figure 3A). B) The degree of criterion shift for each condition. The pattern roughly replicated the one in main analyses, such that reward conditions had smaller criterion shift. However, there is no significant difference between confidence condition and two expectation conditions (*p* = 0.32 and 0.45 for pre-expectation and post-expectation conditions, respectively). Furthermore, post-cue conditions had significantly greater criterion shift than pre-cue conditions (pre-expectation vs. post-expectation, *p* = 0.005; pre-reward vs post-reward, *p* = 0.033). C) d’ values associated with three criterion locations ($c_{-1},c_{0},c_{1}$) in each condition. The pattern is identical to the main analyses (Figure 4A), except that the $d’$ for $c_{0}$ was significantly higher than $c_{-1}$ (*p* = 0.007). D) The degree of d’ drop for each condition. The pattern is identical to the main analyses (Figure 4B) so that confidence and pre-expectation conditions had similar degree of $d’$ drop and they were higher than the ones in other conditions. However, the d′ drop in the post-expectation condition was significantly greater than in the post-reward condition (*p* = 0.031), and the d′ drop in the pre-reward condition was also significantly greater than in the post-reward condition (*p* = .030). Overall, these results were in line with the main manuscript's findings. Box plots show 25^th^, 50^th^, and 75^th^ percentiles (box lines), as well as minimum and maximum excluding outliers (whiskers). Individual dots show outliers. Diamonds show means.***, p < .001; **, p < .01; *, p < .05; n.s., not significant.

**Figure S5. Split-half reliability results.** A) Across-subject correlations in criterion shift between odd and even trials in each condition. The results demonstrate a medium reliability (r-value between .48 and .75) suggesting that caution needs to be used when criterion shift is correlated with other variables. B) Across-subject correlations in $d’$ drop between odd and even trials in each condition. The results demonstrate a low reliability (r-value between -.10 and .47) suggesting that $d’$ drop cannot be computed with sufficient across-subject reliability to uncover meaningful correlations with other variables. In other words, conducting between-condition correlation analyses on $d’$ drop cannot yield meaningful results.

**Figure S6. Between-condition correlations**. Across-subject, one-sided Pearson’s correlation tests for all pairs of conditions. A) Criterion shift correlations across conditions. We found significant positive correlations within the four cues conditions, revealing that the degree of criterion shift within cues conditions significantly correlated with each other, while confidence condition only significantly correlated with reward conditions. Note that the low correlations are likely driven by the low reliability of the criterion shift measure (see Figure S5A). B) $d’$ drop correlations across conditions. We found that $d’$ drop was only significantly correlated within expectation conditions and within reward conditions. Nevertheless, given the extremely low reliability of the $d’$ drop measure (see Figure S5B), these results should be interpreted with extreme caution because low reliability can make even very high correlations between the underlying constructs disappear. *****, *p* < .001; ***, p* < .01; *n.s.*, not significant.

**Figure S7. Replication with the subset of subjects used in model fitting.** In the model fitting procedure, we included a subset of 155, 126, 123, 151, and 155 subjects for confidence, pre-expectation, post-expectation, pre-reward, and post-reward conditions. Notably, these subjects were those whose data did not need the modified formulas when computing criterion shift and $d’$ drop. Here we examined whether we could replicate the main findings on those subjects. A) Criterion location in each condition for the three criterion locations ($c_{-1},c_{0},c_{1}$). This pattern is identical to the main analyses (Figure 3A). B) The degree of criterion shift for each condition. The pattern roughly replicated the one in main analyses. However, there is no significant difference between confidence condition and post-expectation conditions (*p* = 0.125), while the difference between pre-reward and post-reward conditions was significant (*p* = 0.04). C) $d’$ values associated with three criterion locations ($c_{-1},c_{0},c_{1}$) in each condition. The pattern replicated the one in main analyses, except that the $d’$ for biased criteria was not significantly lower than the one for unbiased criteria in both reward conditions (all *p*’s > 0.128). D) The degree of $d’$ drop for each condition. The pattern was identical to that observed in the main analyses (Figure 4B), such that the d′ drop in the confidence and expectation conditions was greater than that in the reward conditions. However, there was no significant difference between the confidence and post-expectation conditions (*p* = 0.96), nor between the pre-expectation and post-expectation conditions (*p* = 0.167). In addition, we observed a significant difference between the post-expectation and two reward conditions (*p*’s < 0.012). Overall, these results were in line with the main manuscript's findings. Box plots show 25th, 50th, and 75th percentiles (box lines), as well as minimum and maximum excluding outliers (whiskers). Individual dots show outliers. Diamonds show means *****, *p* < .001; ***, p* < .01; **, p* < .05; *n.s.*, not significant.

**Table S1. Pairwise comparisons for log-transformed computational noise values.**

| **Comparison** | **N pairs** | **p (Permutation)** | **Boot 95% CI** | **p (Bootstrap)** |
| --- | --- | --- | --- | --- |
| Confidence vs Pre-Expectation | 70 | .335 | [-0.286, 0.100] | .333 |
| Confidence vs Post-Expectation | 68 | .796 | [-0.173, 0.130] | .779 |
| Confidence vs Pre-Reward | 86 | .267 | [-0.054, 0.211] | .251 |
| Confidence vs Post-Reward | 89 | .351 | [-0.073, 0.210] | .348 |
| Pre-Expectation vs Post-Expectation | 75 | .406 | [-0.086, 0.215] | .403 |
| Pre-Expectation vs Pre-Reward | 84 | .137 | [-0.028, 0.264] | .122 |
| Pre-Expectation vs Post-Reward | 88 | .375 | [-0.086, 0.236] | .381 |
| Post-Expectation vs Pre-Reward | 79 | .653 | [-0.113, 0.185] | .651 |
| Post-Expectation vs Post-Reward | 78 | .236 | [-0.051, 0.232] | .223 |
| Pre-Reward vs Post-Reward | 98 | .566 | [-0.142, 0.073] | .554 |
